# Genome-wide analysis of cotton C2H2-zinc finger transcription factor family and their expression analysis during fiber development

**DOI:** 10.1186/s12870-019-2003-8

**Published:** 2019-09-11

**Authors:** Haron Salih, Magwanga Richard Odongo, Wenfang Gong, Shoupu He, Xiongming Du

**Affiliations:** 1 College of life sciences, Huazhong Agricultural University, Wuhan, 430070 Hubei China; 2 State Key Laboratory of Cotton Biology/ Institute of Cotton Research, Chinese Academy of Agricultural Sciences, Anyang, 455000 Henan China; 3 Zalingei University, Central Darfur, Sudan

**Keywords:** Comparative genomics analysis, C2H2-zinc finger family, Cotton, Fiber development, Phylogenetic

## Abstract

**Background:**

C2H2-zinc finger protein family is commonly found in the plant, and it is known as the key actors in the regulation of transcription and vital component of chromatin structure. A large number of the C2H2-zinc finger gene members have not been well characterized based on their functions and structure in cotton. However, in other plants, only a few C2H2-zinc finger genes have been studied.

**Results:**

In this work, we performed a comprehensive analysis and identified 386, 196 and 195 C2H2-zinc finger genes in *Gossypium hirsutum* (upland cotton), *Gossypium arboreum* and *Gossypium raimondii*, respectively. Phylogenetic tree analysis of the C2H2-zinc finger proteins encoding the C2H2-zinc finger genes were classified into seven (7) subgroups. Moreover, the C2H2-zinc finger gene members were distributed in all cotton chromosomes though with asymmetrical distribution patterns. All the orthologous genes were detected between tetraploid and the diploid cotton, with 154 orthologous genes pair detected between upland cotton and *Gossypium arboreum* while 165 orthologous genes were found between upland cotton and *Gossypium raimondii*. Synonymous (Ks) and non-synonymous (Ka) nucleotide substitution rates (Ka/Ks) analysis indicated that the cotton C2H2-zinc finger genes were highly influenced mainly by negative selection, which maintained their protein levels after the duplication events. RNA-seq data and RT-qPCR validation of the RNA seq result revealed differential expression pattern of some the C2H2-zinc finger genes at different stages of cotton fiber development, an indication that the C2H2-zinc finger genes play an important role in initiating and regulating fiber development in cotton.

**Conclusions:**

This study provides a strong foundation for future practical genome research on C2H2-zinc finger genes in upland cotton. The expression levels of C2H2-zinc finger genes family is a pointer of their involvement in various biochemical and physiological functions which are directly related to cotton fiber development during initiation and elongation stages. This work not only provides a basis for determining the nominal role of the C2H2-zinc finger genes in fiber development but also provide valuable information for characterization of potential candidate genes involved in regulation of cotton fiber development.

**Electronic supplementary material:**

The online version of this article (10.1186/s12870-019-2003-8) contains supplementary material, which is available to authorized users.

## Background

Zinc finger protein family is one of the most abundant transcription factors found in higher plants [1]. Furthermore, the zinc finger protein is a unique type of protein domain in which a zinc ion is bounded by cysteine and histidine residues [2], and mainly categorized into different types, namely C_2_H_2_, C_2_HC, C_2_HC_5_, C_2_C_2_, CCCH, C_3_HC^4^, C_4_, C_4_HC_3_, C_6_, and C_8_ based on the position and number of histidine and cysteine residues [3]. C_2_H_2_-zinc finger proteins, also referred as TFIIIA-type finger proteins with the general formula of X_2_CX_2_–4CX_12_HX_2_–8H, where X represents the amino acid, C represents cysteine while H represents histidine, form one of zinc finger proteins family which has been well-characterized in various plants species [4], with a wide distribution within the plant kingdom [3, 5]. The C2H2-zinc finger proteins were first discovered in Petunia [6]. To date, there are 176, 189 and 124 C2H2-zinc finger genes so far identified in Arabidopsis, rice and foxtail millet, respectively [3], 109 in *Populus trichocarpa* [7] and 211 in maize [8]. In previous studies of the C2H2-zinc finger transcription factor proteins, they have been found to be vital in promoting plant growth and development [9]. C2H2-zinc finger proteins form a major portion of proteins in higher organism genomes [10]. They play different functions, which includes recognition of DNA, packaging of RNA, activation of transcriptional, apoptosis regulation, assembly and folding protein and also in binding of lipids [11]. In addition, C2H2-zinc finger transcription factor proteins are broadly involved in various processes such as biotic and abiotic stress [12], leaf trichome initiation [13], floral organelles [14], seed germination and primary microRNA biogenesis in Arabidopsis [15]. In rice, soybean and poplar, C2H2-zinc finger proteins do enhance adaptation to cold and drought stress [16–18]. It has been reported that C2H2-zinc finger gene do promoted pathogen defense in *Capsicum annuum* [19]. Moreover, a novel gene, *AtGIS* from arabidopsis, was found to promote trichome development in Transgenic tobacco [20]. In cotton, C2H2-zinc finger protein family was down-regulated at the fiber initiation stage in fuzz-less and lint-less (*fl*) mutant [21] and it was found that several C2H2-zinc finger genes were down-regulated in Ligon-lintless-1 and up-regulated in wild-type during cotton fiber elongation stage [22]. Recently, C2H2-zinc finger genes have been suggested as the candidate genes controlling cotton fiber development in the Ligon-lintless-2 mutant compared to the wild-type [23]. Cotton is largely planted for both natural fiber and seed oil production [24] in more than 80 countries throughout the world [25]. Advancement in spinning technology has created the demand for high fiber quality in terms of length and fineness, moreover, cotton fiber developmental process occurs through four overlapping stages, namely, initiation, elongation, secondary cell wall formation and maturation [25] Fiber initiation, elongation and secondary cell wall have a great impact on the number, length and fineness of fibers, which are the main factors determining lint quality and quantity of yield [25]. Elongation stage of cotton fiber starts immediately after initiation stage and continues for 3 weeks after which the fiber cell switch to intensive deposition of secondary cell wall [26] Cotton fiber is not only the natural resource of the textile industry in the world but also an excellent system to examine gene expression in cotton fiber development [26]. Therefore, deep knowledge of the molecular basis of cotton fiber development will provide needed information how to improve cotton fiber length, which is the main factor in determining fiber quality in the textile industry. Comprehensive analysis and characterization of the cotton C2H2-zinc finger proteins and their evolutionary time in allopolyploid cotton maybe beneficial to reveal critical genes or molecular mechanisms involved in cotton fiber development.

In addition, the current published *G. hirsutum* (upland cotton) genome sequence [27], *G. arboreum* [28] and *G.raimondii* [29], provide the valuable information needed to identify and characterize the whole C2H2-zinc finger proteins in cotton. Based on phylogenetic analysis, No orthologous genes were detected between upland cotton and other plant species such as mays, cacao, *V. vinifera*, Arabidopsis and *P. trichocarpa*. *G. raimondii and G. arboreum* underwent whole genome duplication events about 16.6 million years ago (Mya), and upland cotton (allotetraploid) emerged from hybridizations of A or D diploid ancestral species nearly 1.5 Mya, which produce high quality of fiber as compare to their diploid relatives. To understand if at all increase or decrease of the number of the Zinc figer genes could have resulted into any changes in the dynamics of cotton fiber development. Moreover, upland cotton is a polyploid species in which its genome contains both maternal genome (*G. arboreum* like A-genome) and paternal genome (*G. raimondii* like D genome) [30]. Minimal information is available on cotton in relation to C2H2-zinc finger proteins compared to other plant species [23]. In higher plants, C2H2-zinc finger proteins are one of the major transcription factor families, which could be playing a crucial role in regulating different pathways of fiber development in cotton [2]. Here we performed a comprehensive analysis of cotton C2H2-zinc finger proteins family and their expression analysis in cotton fiber development. As a result, a total of 386, 196 and 195 C2H2-zinc finger proteins were identified in *G. hirsutum*, *G. arboreum* and *G. raimondii,* and their chromosomal positions, duplicated gene events, phylogenetic relationships, gene structures, conserved motifs and expression profiles at different developmental stages were analyzed.

## Results

### Identification of C2H2-zinc finger protein family in cotton

In the identification of all the C2H2- zinc finger gene members, in the whole cotton genome, Hidden Markov Model (HMM) profile of C2H2-zinc finger protein domain (PF00096), obtained from Pfam database (http://pfam.sanger.ac.uk/) and used to query against entire cotton proteome sequence. We identified 420, 236 and 230 C2H2-zinc finger candidate genes in upland cotton, *G. arboreum* and *G. raimondii,* respectively. Additionally, the retrieved sequences were physically checked by SMART (http://smart.emblheidelberg.de/) to examine the presence or absence of the C2H2-zinc finger protein domain. Finally, 386, 196 and 195 C2H2-zinc finger genes were identified in upland cotton, *G. arboreum* and *G. raimondii,* respectively and contained one or more C2H2-zinc finger protein domains (Additional file 5: Table S1). The result was not in line with Plant Transcription Factor Database (http://planttfdb.cbi.edu.cn/) [31] in which only 318 members of the C2H2-zinc finger family genes were found in *G. hirsutum*. The difference could be due to improved gene annotation. The lengths of all upland cotton C2H2-zinc finger proteins had a range of 101 to 1614 amino acids with an average of 374 amino acids. In order to understand the possible function of the proteins encoded by the gene under investigation, understanding their physiochemical properties is inevitable, for example the proteins can be separated according to their molecular mass (size) and isoelectric point (charge) properties and their abundance then determined subsequently [32]. The isoelectric point property of the enzyme is of significance. Through the enzyme movement on the carrier, the bumper should have a pH value supporting electrostatic interactions with the surface of carrier [32]. Moreover, in estimating the any given protein family in plants, different physiological properties are examined, for example in sucrose synthase protein family in cotton, isoelectric points, molecular weights among others were factored [33]. The molecular mass was investigated by ExPaSy analysis to identify their molecular weights, which largely varied between 11,059.09 and 182,448.43 Da (Dalton) with an average of 41,473.5 Da. The prediction of the subcellular location of 386 C2H2-zinc finger proteins was carried out by WoLF PSORT analysis, and the result showed that 351 of C2H2-zinc finger proteins were localized in the nucleus, which could be in line with their functions of interaction with DNA [1]. However, only 35 C2H2-zinc finger proteins were located in different subcellular membranes, such as chloroplast, vacuolar, plastid cytosol and mitochondria. The detailed information of C2H2-zinc finger proteins is listed in (Additional file 6: Table S2), including protein domains, protein lengths, molecular weights and subcellular positions. Additionally, a greater portion of C2H2-zinc finger genes were identified in upland cotton than the diploid cotton genome, since *G. hirsutum* is a polyploid, having emerged through whole genome duplication (WGD) [34].

### Chromosomal distribution of cotton C2H2-zinc finger genes

**Fig. 1:**
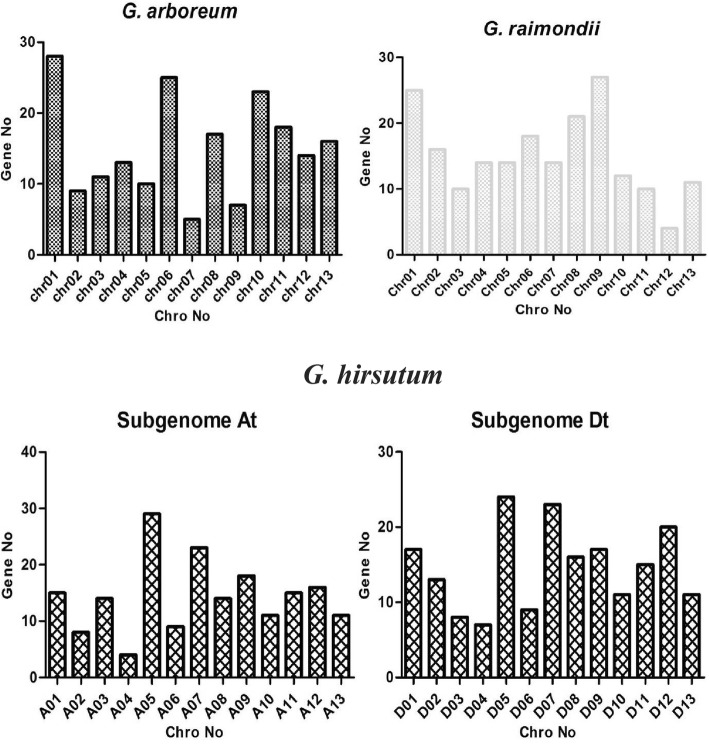
Chromosomal distribution of upland cotton C2H2-zinc finger family. The chromosome location is referred to the gff3 cotton genome dataset

To investigate the chromosomal locations of C2H2-zinc finger genes, based on their positions, datasets retrieved from the whole cotton genome sequence were used. Three hundred and seventy-eight (378) upland cotton C2H2-zinc finger genes were mapped across in all chromosomes and names assigned as per their chromosomal locations as *GhZF* 1 to *GhZF* 378, while only 8 *GhZF* genes were not mapped to any chromosome as referred to scaffolds and designated as *GhZF* 379 to *GhZF* 386. One hundred and ninety-six (196) *G. arboreum* (A_2_) and 195 *G. raimondii* (D_5_) C2H2-zinc finger genes were all mapped to chromosomes and named based on their chromosomal positions. The distribution of C2H2-zinc finger genes on different chromosomes was not uniform (Fig. 1); for instance; some chromosomes and loci, had a high density of C2H2-zinc finger genes while others do not (Fig. 1 and Additional file 5: Table S1). The highest density of C2H2-zinc finger genes was detected on chromosome A05 (At) and its homolog chromosome D05 (Dt) with 53 genes while the lowest density was detected on chromosome A04 and its homolog chromosome D04, with 11 genes (Additional file 1: Figure S1). Furthermore, relatively the great numbers of C2H2-zinc finger genes were located at the specific regions of some chromosomes, such as the upper and lower centromeric regions. In *G. arboreum,* the highest number of C2H2-zinc finger genes was identified on chromosome 1 with 28 and the lower density in chromosome 9 with 7 genes. Whereas in *G. raimondii,* chromosome 9 had the highest number of C2H2-zinc finger genes with 27 while chromosome 12 exhibited the least number of 4 genes (Fig. 1).

### Gene duplication events of upland cotton C2H2-zinc finger genes

Gene duplication, tandem and segmental duplication events have been demonstrated to be the two main mechanisms initiating gene family expression in plants [35]. The duplication events were investigated with the aim to explain the diverse mechanism of the upland cotton C2H2-zinc finger transcription factor family, which had arose during the process of whole genome evolution [36]. Based on the alignment sequence lengths and the similarity of the aligned regions, 37 gene pairs were identified to be involved in both tandem and segmental duplication events, with 36 gene pairs, At and Dt, exhibiting segmental duplication and only single pair, in chromosome A_5_, with tandem duplication (Table 1). To explore the mechanism of C2H2-zinc finger genes divergence after polyploidization duplication, the non-synonymous (Ka), synonymous (Ks) and Ka/Ks were estimated for the homologous gene pair in upland cotton *G. hirsutum*. If, the value of Ka/Ks more than 1 means positive selection or the evolution under advantageous selection and some of the mutation events most be profitable. If, Ka/Ks ratio = 1 means neutral selection. Ka/Ks ratio less than 1 means negative selection or the mutation restriction has the disadvantageous effect, in others literature, it is termed as purifying selection. Out of 37 duplicated gene pairs, 29 had a Ka/Ks ratio lower than 1, suggesting that C2H2-zinc finger genes have evolved mainly under the effect of negative selection or their mutations had the disadvantageous effect. Ka/Ks ratio less than 1 gives a strong indication of C2H2-zinc finger genes having undergone slow evolution and have conserved characteristics at the protein level after the duplication events. However, only 8 C2H2-zinc finger genes had a Ka/Ks ratio more than 1, suggesting that those genes have been evolved by positive selection. It is a remarkable evidence to notice the majorities of upland cotton C2H2-zinc finger genes were evolved under negative selection. Furthermore, we used Ks to calculate the time of duplication events during the evolutionary time of the upland cotton genome. The tandem and segmental repetition events in upland cotton occurred between 0.25 and 3.56 mya (million years ago) with an average of 1.05 mya which is consistent to whole genome duplication of upland cotton [27, 37]. The results suggest that the expansion of the C2H2-zinc finger genes in upland cotton, which originated from A_2_ and D_5_ genome, mostly arose from whole genome duplication events during their evolution.

**Table 1:** Synonymous (Ks) and non-synonymous (Ka) substitution rates are represented for each gene pairs and the estimated time for the tandem and segmental duplication events C2H2-zinc finger genes

Gene 1	Gene 2	Ka	Ks	Ka/Ks	Negative selection	Duplicated time (Mya)
Gh_A01G1610	Gh_D01G1850	0.0147	0.0446	0.329596	Yes	1.486667
Gh_A04G0724	Gh_D04G1191	0.0164	0.0404	0.405941	Yes	1.346667
Gh_A05G0618	Gh_D05G0749	0.0324	0.0331	0.978852	Yes	1.103333
Gh_A05G2644	Gh_D05G2946	0	0	0	Yes	0
Gh_A05G2922	Gh_A05G2923	0	0	0	Yes	0
Gh_A05G3205	Gh_D04G0401	0.0161	0.0546	0.294872	Yes	1.82
Gh_A06G0175	Gh_D06G0169	0.0042	0.0076	0.552632	Yes	0.253333
Gh_A06G1535	Gh_D06G1905	0.0374	0.0634	0.589905	Yes	2.113333
Gh_A09G0835	Gh_D09G0856	0.0127	0.031	0.409677	Yes	1.033333
Gh_A10G1009	Gh_D10G1529	0.0286	0.069	0.414493	Yes	2.3
Gh_A10G1921	Gh_D10G2215	0.0247	0	0	Yes	0
Gh_A13G0183	Gh_D13G0198	0.0192	0.0199	0.964824	Yes	0.663333
Gh_A13G2132	Gh_D13G0718	0.0028	0	0	Yes	0
Gh_D02G1695	Gh_A03G1255	0.0167	0.0102	1.637255	No	0.34
Gh_D02G2295	Gh_A03G1856	0.0204	0.028	0.728571	Yes	0.933333
Gh_D05G0854	Gh_A05G0718	0.0148	0.0154	0.961039	Yes	0.513333
Gh_D05G0878	Gh_A05G0747	0.0028	0	0	Yes	0
Gh_D05G1143	Gh_A05G1026	0.018	0	0	Yes	0
Gh_D05G1990	Gh_A05G1795	0.0379	0	0	Yes	0
Gh_D05G2011	Gh_A05G1815	0.0339	0.0126	2.690476	No	0.42
Gh_D06G0302	Gh_A06G0282	0.0104	0	0	Yes	0
Gh_D06G0903	Gh_A06G0780	0.0064	0.0443	0.14447	Yes	1.476667
Gh_D07G2141	Gh_A07G1918	0	0.0309	0	Yes	1.03
Gh_D09G1495	Gh_A09G1485	0.0166	0.0131	1.267176	No	0.436667
Gh_D09G1496	Gh_A09G1486	0.0167	0.0258	0.647287	Yes	0.86
Gh_D10G1973	Gh_A10G1704	0.0412	0.1067	0.386129	Yes	3.556667
Gh_D10G2214	Gh_A10G2356	0.0374	0	0	Yes	0
Gh_D10G2304	Gh_A10G1997	0.0275	0.01	2.75	No	0.333333
Gh_D11G0560	Gh_A11G0482	0.0217	0.0436	0.497706	Yes	1.453333
Gh_D11G2018	Gh_A11G1957	0.0455	0.0141	3.22695	No	0.47
Gh_D11G2046	Gh_A11G1928	0.0106	0.0149	0.711409	Yes	0.496667
Gh_D12G2156	Gh_A12G1978	0.0111	0.0092	1.206522	No	0.306667
Gh_D13G0287	Gh_A13G0268	0.023	0.032	0.71875	Yes	1.066667
Gh_D13G0838	Gh_A13G0716	0.0241	0.0149	1.61745	No	0.496667
Gh_D13G1149	Gh_A13G0909	0.0419	0.0318	1.31761	No	1.06
Gh_D13G1953	Gh_A13G1592	0	0	0	Yes	0
Gh_D13G2392	Gh_A13G1993	0	0.0277	0	Yes	0.923333

### Relation between C2H2-zinc finger proteins cotton with other plants

**Fig. 2:**
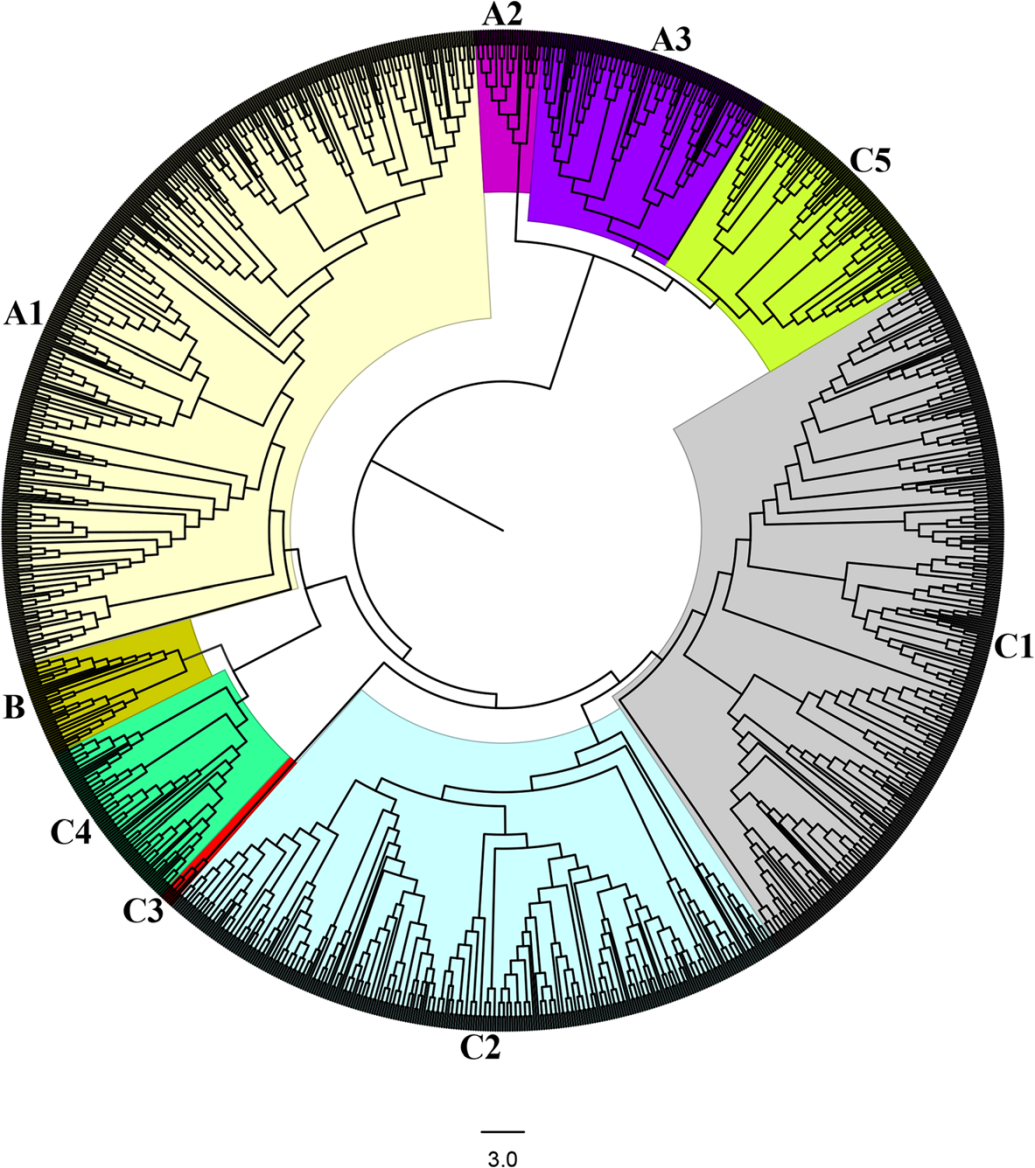
Phylogenetic tree relationships between 386 upland cotton, 196 *G. arboreum,* 195 *G. raimondii,* 97 *T. cacao*, 179 *Z. mays*, 64 *V. vinifera*, 118 *P. trichocarpa* and 60 *A. thaliana* C2H2-zinc finger proteins. The phylogenetic tree was constructed by MEGA 6.0 using the Neighbor-Joining method. The bootstrap test was performed with 1000 iterations. The nine subgroups are shown with different colors

Further analyze the evolutionary relationship of C2H2-zinc finger protein family in cotton and other plants 386 upland cotton, 196 *G. arboreum,* 195 *G. raimondii,* 97 *T. cacao*, 179 *Z. mays*, 64 *V. vinifera*, 118 *P. trichocarpa* and 60 *A. thaliana* were used to construct phylogenetic tree, by employing the neighbor-joining (NJ) method of the MEGA 6 software. Based on the sequence similarity and tree topology, the C2H2-zinc finger proteins were divided into 3 main groups, designated as A, B and C (Table 2). The numbers of C2H2-zinc finger proteins in three groups were different, group C contained the largest number, followed by group A, while group B had the least number of the C2H2-zinc finger proteins. The result is in agreement with earlier findings in other plants on quantification of these genes in various groupings [2]. Finally, three main groups (A, B and C) were further subdivided into different subgroups A1, A2, A3, B and C1 to C5 (Fig. 2 and Table 2). In this subdivision, we considered the previous annotation of groups in other plants where this family has been deeply characterized, like *Arabidopsis* [2], *Z. mays* [8] and *P. trichocarpa* [7]. In general, the highest numbers of these genes were detected in subgroup A1 with 366, closely followed by the subgroup C1 with 312 genes while subgroup C3 contained the least number of C2H2-zinc finger genes with only 6. The result showed that 8 plant species contained the 7 subgroups of C2H2-zinc finger genes, which provides strong evidence that the divergence of these plant species occurred after the extension of C2H2-zinc finger transcription factor family. The distribution of C2H2-zinc finger genes was much higher in the cotton genome than other plants, within the cotton genome, upland cotton contained highest numbers of C2H2-zinc finger genes in all subgroups except subgroups B and C3 (Table 2). Additionally, a unique observation was made, in which some clades were purely composed of members of genes derived from a specific plant species, these genes are referred as paralogous genes pairs, being paralog genes diverge from one another within a species [38]. A comparative analysis was done in order to identify orthologous C2H2-zinc finger genes among upland cotton, *G. arboreum, G. raimondii, Z. mays, T cacao*, *V. vinifera*, *A .thaliana* and *P. trichocarpa*. No orthologous genes were detected between upland cotton and other plant species, but a total of 154 orthologous gene pairs were found between upland cotton and *G. arboreum* and 165 between upland cotton *G. raimondii.* All of the orthologous genes mainly existed between upland cotton and diploid cotton*,* which explain the origin of upland cotton from *G. raimondii* and *G. arboreum*. This finding implies that the C2H2-zinc finger genes emerged mainly from the whole genome duplication events during their evolution period. The results provided an indication that cotton C2H2-zinc finger proteins were more closely related to C2H2-zinc finger proteins from cacao than any other plant species, thus consistent with the earlier report which indicated that cotton and cacao emerged from the same ancestor [28].

**Table 2:** Size of the C2H2-zinc finger genes subgroup in different plant species

Subgroup	Upland cotton	*G. raimomdii*	*G. arboreum*	*Arabidopsis*	*Zea mays*	*Theobroma cacao*	*Vitis vinifera*	*Populus trichocarpa*
A1	108	52	53	14	55	32	27	22
A2	8	4	4	1	7	3	1	0
A3	24	15	12	5	16	11	7	7
B	10	5	8	1	10	3	2	2
C1	95	48	48	17	36	21	9	38
C2	87	42	42	13	36	13	10	25
C3	2	1	2	1	0	0	0	0
C4	22	11	11	3	11	5	4	15
C5	30	17	16	5	8	9	4	9
Totals	386	195	196	60	179	97	64	118

In order to explore the mechanism of C2H2-zinc finger genes divergence after polyploidization duplication, the Ka, Ks and Ka/Ks values were estimated for the orthologous gene pairs between *G. arboreum (*A_2_) and *G. hirsutum* (At sub genome), as well as those between *G. raimondii* (D_5_) and *G. hirsutum* (Dt sub genome). The value of Ka/Ks ratio is an indicator of selective pressure acting on a protein coding gene during evolutionary time (Additional file 7: Table S3). Based on the result, we found that 152 C2H2-zinc finger gene pairs A2/At, 147 had a Ka/Ks ratio less than 1, indicating that C2H2-zinc finger genes have primarily evolved under the effect of stabilizing selection among three cotton species. However, only 5 C2H2-zinc finger genes had a Ka/Ks ratio more than 1, thus evolved through positive selection. Moreover, 165 C2H2-zinc finger gene pairs D5/Dt, 151 had a Ka/Ks less than 1 indicative of negative selection. While only, 13 gene pairs had a Ka/Ks ratio more than 1 suggested positive selection and 1 gene pair had a Ka/Ks ratio = 1 suggested neutral section (Additional file 7: Table S3). It is significant to note that the greater percentage of orthologous upland cotton C2H2-zinc finger genes with either *G. arboreum* or *G. raimondii* were evolved under the influence of stabilizing selection, thus these genes evolved slowly and have conserved characteristics at the protein level after the duplication events.

### Phylogenetic tree, gene structure and conserved motif analysis of the upland cotton C2H2-zinc finger gene family

The evolutionary relationships between members of the upland cotton C2H2-zinc finger protein family were further examined by carried out, a comprehensive phylogenetic tree analysis by building an unrooted phylogenetic tree using the neighbor-joining method, with 1000 bootstrap replicates. Based on the protein sequence similarity, the upland cotton C2H2-zinc finger proteins family was classified into seven subgroups (I to VII), which varied in number from 4 to 105 genes (Additional file 2: Figure S2A). The reliability of phylogenetic tree was tested by minimum evolution method. The trees created by the two methods mentioned above were identical, implying that the two methods were consistent with each other (Additional file 3: Figure S3).

Further investigation of the exon-intron structure in upland cotton C2H2-zinc finger genes, 51.6% (199) of C2H2-zinc finger transcription factors were intronless and 48.4% (187) of C2H2-zinc finger genes had introns, which varied from 1 to 10. Moreover, most C2H2-zinc finger genes with the intron, 101 contained either 1 or 2 introns while the rest were disrupted by more than two introns (Additional file 2: Figure S2B). However, the majority of the intronless (199) genes were clustered into subgroups I and II. Furthermore, the intron-exon similarities within C2H2-zinc finger gene subgroups were further evaluated through phylogenetic stress analysis. In addition, most C2H2-zinc finger genes which were clustered together showed high similarity in the exon-intron organization, both in intron lengths and exon numbers. For example, 95 genes clustered in subgroup I, greater numbers were intronless with the exception of only 6 genes which had a single intron. The C2H2-zinc finger genes in subgroups V and VI showed a wide divergence in exon length and intron numbers, which varies from 0 to 10 (Additional file 2: Figure S2B). The exon-intron structures of upland cotton C2H2-zinc finger genes were fundamentally consistent with the phylogenetic tree analysis.

Further analysis was done in order to determine the diversity of conserved motifs among 386 C2H2-zinc finger proteins from subgroups I to VII, MEME tool was used. Twenty conserved motifs were identified and designated as motif 1 to motif 20 in C2H2-zinc finger proteins (Additional file 2: Figure S2D). Most C2H2-zinc finger proteins within the same subgroups had a common motif in term of motif composition and distribution, which implied the C2H2-zinc finger protein members within a given subgroup, could be having similar functions. However, a great divergence was also exhibited between different subgroups. For example, all protein sequences in subgroup I had motifs 1 and 7, which contained QALGGH and WSKRKRSKRPR motifs, while subgroup II had motifs 1, 2 and 4 contain two domain of QALGGH motifs, and a single of LDLDL motif (Additional file 4: Figure S4), which were previously described in higher plants [3]. In subgroups, IV and V had motif 5 described as ALGGH (Additional file 2: Figure S2D). Conserved motifs 1 and 2 contained QALGGH and ALGGH motifs, which were previously reported to be involved in abiotic stress response during plant growth and development [3]. Moreover, some unique motifs were observed in a specific subgroup, which provided a stronger indication of involvement of these motifs in specific functions within the plants.

In order to determine the similarity among the upland cotton C2H2-zinc finger proteins, we aligned 386 upland cotton C2H2-zinc finger protein sequences for each subgroup, I to VII (Additional file 8: Table S4). Based on the result of multiple sequence alignment, the entire upland cotton C2H2-zinc finger protein we found harbor three major motif domains Q-type (QALGGH motifs), Z-type and C-type, 196, 105 and 85 upland cotton C2H2-zinc finger proteins respectively. Q-type domain contained two major motifs QALGGH and ALGGH, besides the two, some proteins sequences contained LDLDL, FDLDL and IDLDL motifs, which were previously reported to play a vital role in the defense system of plants [3]. Furthermore, Q-type containing zinc finger, though the cysteine residue at the second position changed by tyrosine and clustered in subgroups I, II, IV and V as shown in yellow color (Additional file 8: Table S4). Z-type with zinc fingers had extremely conserved motifs in finger and the flanking regions and previously annotated to be C-type [39]. All the upland cotton C2H2-zinc finger genes belong to Z-type was clustered in subgroup VII as shown in green color (Additional file 8: Table S4). C-type of zinc finger had no conserved motif in the zinc finger region compared to Q-type, Z-type and mainly clustered in subgroups III and VI. In summary, the distributions of motif between C2H2-zinc finger proteins and protein types, Q-type, Z-type and C-type, are strongly supported the evolutionary relationships and the reliability of the phylogenetic tree analysis.

### Expression profiles of upland cotton C2H2-zinc finger genes at different developmental stages

Gene expression levels and function are highly correlated and provide vital information on whether the C2H2-zinc finger genes are actually involved in the process under investigation. C2H2-zinc finger genes are largely involved in various mechanisms of plant cell differentiation and development such as trichome initiation [40], floral organelles [14] and root hair development [41]. The expression levels of all upland cotton C2H2-zinc finger genes was done on various plant tissues such as root, stem and leaves and fiber development at 0, 3, 5, 10, 20 and 25 DPA, using publicly available RNA-seq data (PRJNA248163) [27]. It was shown that 378 C2H2-zinc finger genes were expressed in at least one tissue or stage of cotton fiber development, while only 8 upland cotton C2H2-zinc finger genes their expression could not be detected by RNA-seq analysis (Additional file 2: Figure S2C and Additional file 9: Table S5). In addition, a greater percentage of the C2H2-zinc finger genes showed varying expression levels at different developmental stages of cotton fiber, while a few of them exhibited equal expression levels. Based on the phylogenetic tree analysis, the expression levels of C2H2-zinc finger gene family were divided into 7 subgroups. In subgroup I, contained only one QALGGH motif, a large number of these genes were expressed at lower levels in nearly all of the tissues tested except 4 genes, *Gh_A05G2741*, *Gh_D05G3769*, *Gh_A04G0449* and *Gh_Sca045498G01*, which are homologous to *AT2G41940* gene which encodes the Arabidopsis Zinc finger protein 8, were highly expressed at initiation stage of cotton fiber development at 0 and 3 DPA in wild-type. Two members of subgroup 1, *Gh_D13G0287* and *Gh_A13G0268* were highly expressed at later elongation stage of cotton fiber development at 25 DPA. In stem tissue, the highest expression level was observed in *Gh_D02G1695* and *Gh_A03G1255*, which are homologous to *AT1G10480*, a zinc figure gene type which encodes a zinc finger protein containing only a single zinc finger that acts downstream of ZFP6 in regulating trichome development by integrating gibberellin acid (GA) and cytokinin signaling. [13]. In fiber development stage, these two genes showed higher expression levels at 0 DPA but the significantly lower expression in other fiber development stages (Additional file 2: Figure S2C and Additional file 9: Table S5). This result indicates that these genes could be involved either directly or indirectly in cotton fiber development, mainly intense in initiation stages. In subgroup II, a number of genes, with two QALGGH motifs and those with LDLDL, FDLDL and IDLD motifs, exhibited significantly higher levels as compared to subgroup I. Subgroup II genes including Gh_D05G2011, *Gh_A05G1815*, *Gh_D06G2303*, *Gh_A01G0984*, *Gh_D01G1033*, *Gh_A02G0836*, *Gh_D13G0451*, *Gh_A13G2112* among others. Some of these genes had differential expression during fiber development, implying that they could be involved in the regulation mechanism at a different stage of fiber development in cotton. High numbers of genes containing ALGGH motifs were found to be members of subgroup IV, which includes, *Gh_A09G0743*, *Gh_D09G0744*, *Gh_D10G0401* and *Gh_Sca004883G01* (Additional file 2: Figure S2C). The members of subgroup IV showed similar expression in all the tissues tested but with lower expression levels. In subgroup V, contained 8 genes with ALGGH motifs, had very low expression levels, near zero marks in all cotton tissue tested except, *Gh_D01G1111* and *Gh_A01G1056* which had slightly higher but similar expression pattern across different tissues tested (Additional file 2: Figure S2C). This result gives an indication that these genes with QALGGH and ALGGH motifs might be involved in specific developmental stages under special conditions of cotton plant growth and development. Most of the genes in subgroup III exhibited differential expression in different cotton tissues but highly expressed at initiation and elongation stages of cotton fiber development as compared to the root, leaf and stem tissues. In subgroup VI, a number of genes had higher expression levels than the rest of the groups, genes *Gh_D05G0849*, *Gh_D02G2408*, *Gh_A03G0820*, *Gh_A05G0702*, *Gh_D01G2032*, *Gh_D05G0663*, *Gh_A09G0878* showed differential expression levels in root, stem, leaf and different stages of fiber development, suggesting that member of subgroup VI may be playing an important role in regulation of fiber development in cotton. In subgroup VII, few genes had lower expression levels in various tissues tested while large numbers of genes showed high expression levels in stem and cotton fiber development, including, *Gh_A02G0617*, *Gh_D02G0671* and others. In general, some homologous upland cotton C2H2-zinc finger gene pairs were equally expressed between Dt-sub genome and At-sub genome, for instance, *Gh_D02G1695* and *Gh_A03G1255* showed a similar expression level in stem and cotton fiber development at 5 and 20 DPA. *Gh_A10G1921* and its homologous *Gh_D10G2215* were equally expressed in root, leaf and fiber development at 20 DPA. Paralogous gene pairs with similar expression pattern have higher sequence similarity than the paralogous gene pairs with different expression levels [42]. This result implied that the duplicated expression level of one gene pair was sufficient for maintaining cotton fiber development, while the other might be involved in other regulatory processes.

### C2H2-zinc finger genes and their involvement in cotton fiber mutation

**Fig. 3:**
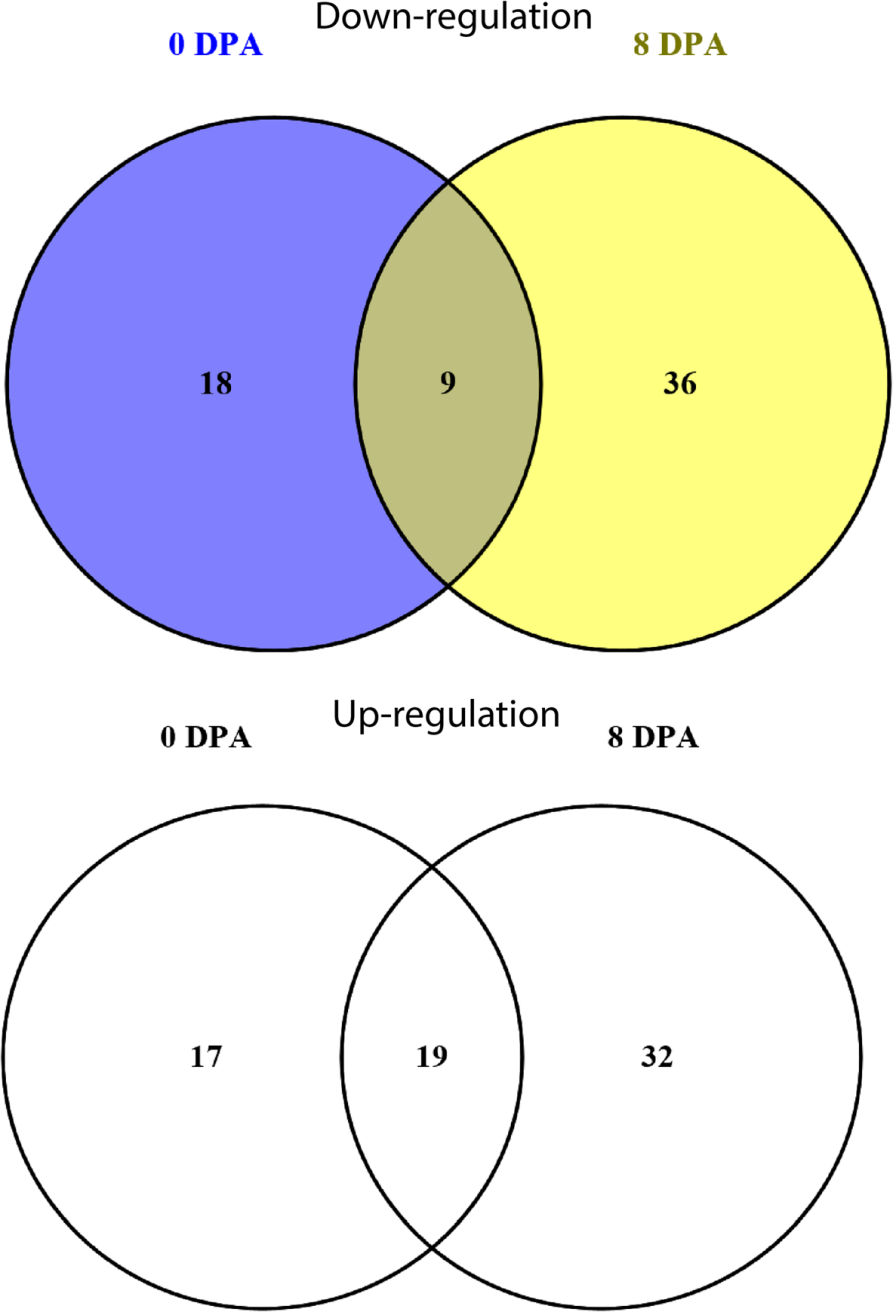
Differentially expressed C2H2-zinc finger genes in Ligon-lintless-1 and wild-type. (A) A Venn diagram showed C2H2-zinc finger genes that were up and down-regulated in Ligon-lintless-1 and wild-type

To get deep insight into the roles of C2H2-zinc finger genes in cotton fiber development, gene expression pattern was analyzed using transcriptome data of RNA-seq obtained from two genotypes, Ligon-lintless-1 mutant and wild-type during fiber development at 0 and 8 DPA. The expression analysis of C2H2-zinc finger genes during initiation and elongation stages of cotton fiber development was investigated. Several C2H2-zinc finger genes were significantly varied in expression levels (fold change ≥2 and *p*-value ≤0.05) between Li1 mutant as compared to the wild-type during initiation (0 DPA) and elongation (8 DPA) stages. The result showed that 63 and 96 C2H2-zinc finger genes were differentially expressed between Li1 mutant and wild-type at 0 and 8 DPA, respectively (Additional file 10: Table S6). A total of 27 and 45 genes exhibited significant down-regulation, while 36 and 51 genes exhibited significant up-regulation in cotton fiber development at 0 and 8 DPA, respectively (Fig. 3 and Additional file 10: Table S6) which provide a clue of being positively or negatively controlling fiber development during initiation and elongation stages of Ligon-lintless-1 and its wild-type. There were more up-regulated C2H2-zinc finger genes than the down-regulated ones in Li1 mutant as compared to wild-type this possibly means these genes could be involved in regulating cotton fiber development in mutation cotton. Moreover, the outcome of this study revealed the majority of the differentially expressed genes were specifically expressed at a specific stage of fiber development (8 DPA), which suggests that these C2H2-zinc finger genes play a key role in regulating various stages of cotton fiber development.

### Verification of C2H2-zinc finger gene expression by RT-qPCR

**Fig. 4:**
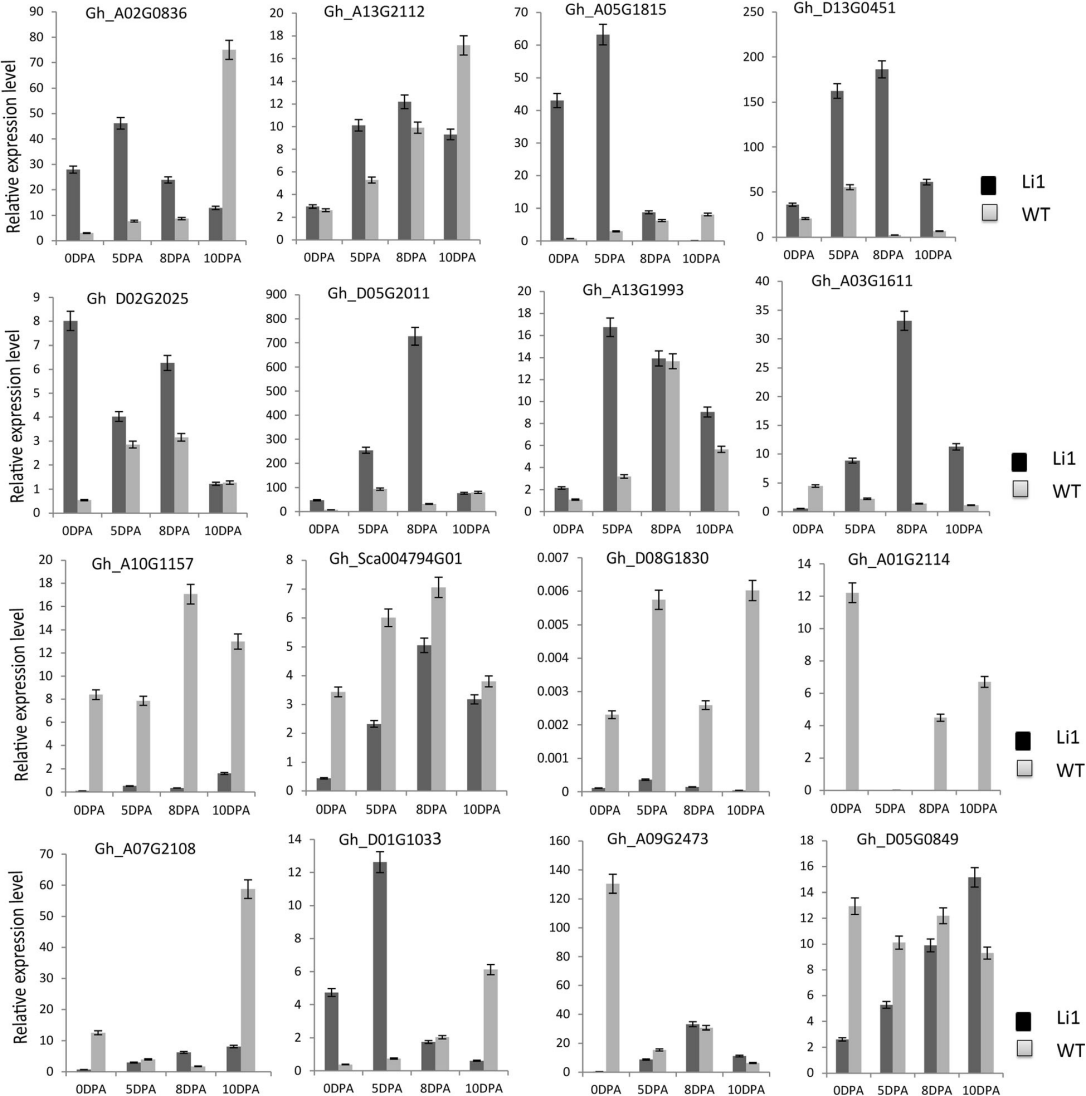
Expression levels of 16 C2H2-zinc finger genes measured by RT-qPCR analysis of Ligon-lintless-1 mutant and wild-type at different cotton fiber development. Black and grey represent the expression levels of Ligon-lintless-1 mutant (Li1) and wild-type (WT), respectively

To examine whether these differentially expressed C2H2-zinc finger genes had a role in cotton fiber development during initiation and elongation stages, 16 C2H2-zinc finger genes were selected based on their expression patterns at initiation and elongation stages of cotton fiber development, 8 up-regulated genes (*Gh_A02G0836*, *Gh_A13G2112*, *Gh_A13G1993*, *Gh_D13G0451*, *Gh_D05G2011*, *Gh_A03G1611*, *Gh_D02G2025* and *Gh_A05G1815*) and 8 down-regulated genes (*Gh_A07G2108*, *Gh_A10G1157*, *Gh_Sca004794G01*, *Gh_D05G0849*, *Gh_A09G2473*, *Gh_D01G1033*, *Gh_A01G2114* and *Gh_D08G1830*), which were differentially expressed at initiation and elongation stages of fiber development. RT-qPCR was used to validate the expression levels of the C2H2-zinc finger gene family in Li1 mutant and wild-type at different stages of cotton fiber, 0, 5, 8 and 10 DPA (Fig. 4). All of the C2H2-zinc finger genes exhibited diverse expression profiles between Li1 mutant and wild-type during cotton fiber development. *Gh_D02G0836*, *Gh_A13G2112*, *Gh_A05G1815*, *Gh_D02G2025* and *Gh_D02G2011* genes were highly expressed in the Li1 mutant than in wild-type at 0, 5 and 8, but not at 10 DPA. The result suggested that this group of genes have an inhibitory role in process fiber development. In addition, *Gh_A13G1993* and *Gh_D13G0451* genes were absolutely expressed in Ligon-lintless-1 with high expression levels across all fiber development (different times tested), but not in wild-type. *Gh_A03G1611* was expressed highly expressed in Ligon-lintless-1 at 5, 8 and 10 DPA but showed low expression level at 0 DPA in Li1 mutant as compared to wild-type. The outcome of this result was consistent with RNA-seq analysis which all these genes were up-regulated in Li1 mutant as compared to its wild-type, indicating these genes could be having role in inhibiting normal processes in fiber initiation and development. In contrast, 8 down-regulated C2H2-zinc finger genes such as *Gh_A10G1157*, *Gh_Sca004794G01*, *Gh_D08G1830* and *Gh_A01G2114* were expressed highly in wild-type had lower expression levels in the Ligon-lintless-1 mutant at various stages of cotton fiber development, which indicate that these are important for normal fiber development in mutant cotton. Other groups of C2H2-zinc finger genes such as *Gh_A07G2108*, *Gh_D01G1033*, *Gh_A09G2473* and *Gh_D05G0849* were expressed at different levels in the wild-type and Ligon-lintless-1 mutant, which could be an indication of functional divergence of C2H2-zinc finger genes during initiation and elongation stages of cotton fiber development.

## Discussion

C2H2-zinc finger proteins family has been identified in *Arabidopsis*, rice and *populus* [2, 7, 8]. Interestingly, no previous reports has been showed in cotton. In this work, we undertook a comprehensive analysis of cotton C2H2-zinc finger gene family and their involvement in cotton fiber development. We identified 386, 196 and 195 non-redundant C2H2-zinc finger genes in upland cotton, *G. arboreum* and *G. raimondii*, respectively (Additional file 5: Table S1). Upland cotton is derived from the natural hybridization between *G. arboreum* and *G. raimondii* and thus, it would be natural find both progeny copies of 196 *G. arboreum* and 195 *G. raimondii* C2H2-zinc finger genes in upland cotton. However, it was detected in the total number of upland cotton C2H2-zinc finger proteins, in which out of 386 in upland cotton, only 317 of those *Gh*C2H2-zinc finger proteins had corresponding protein sequences from *G. arboreum* and *G. raimondii* which are the diploid parental lines. Majority of orthologous genes were found between upland cotton and diploid cotton*,* which is due to the origin of upland cotton having evolved from *G. raimondii* and *G. arboreum*, through polyploidization of the whole genome in their course evolutions [43]. Our results suggested that there could be some aspect of gene lost after hybridization. Cotton C2H2-zinc finger proteins were more closely related to C2H2-zinc finger proteins from *T. cacao* than from any other plant species, cotton and cacao have a common ancestor [28].

The Ka/Ks ratios of the 24 homologous gene pairs were lower than 1 implying that these genes were mainly evolved under negative selection. This finding was in line with the recent report which found that most MYB transcription factor genes are evolved under negative selection [44]. Gene duplication, tandem and segmental, are of great importance for expansion of the number of a gene family [35]. In the evolution and diversification of the C2H2-zinc finger genes family in upland cotton genome duplications, segmental type of gene duplication played a significant role, which also consistent with previous findings [8]. By calculating the date of gene pair duplication, the gene duplication events in the upland cotton C2H2-zinc finger family was in agreement with whole upland cotton genome duplication time scale (1–2 MYA) [27, 37].

Upland cotton C2H2-zinc finger protein sequences showed a high degree of complexity due to the large variation in protein lengths, molecular weight and subcellular location. These results indicate that these genes may play various functions in plant growth and development, according to their exact position within the plant cell, thus concurs with earlier findings, which suggested that this C2H2-zinc finger family are involved in various aspects of plant growth and development [3, 16, 19, 22]. Based on the phylogenetic tree analysis, the majority of C2H2-zinc finger gene within the same subgroup had similar exon-intron organization and conserved motifs but high divergence was showed among the different subgroups. It was suggested that C2H2-zinc finger family members with similar protein arrangements were classified into the same subgroups. In addition, some exon-intron structure and motif compositions were predominantly found in a specific subgroup, which could be related to the functional diversity of that subgroup. There was a strong connection between the intron-exon structure, conserved motifs and phylogenetic tree analysis of the C2H2-zinc finger family in *G. hirsutum.* Multiple alignments result of the C2H2-zinc finger proteins allowed us to identify 3 main subclasses of the C2H2-zinc finger including, Q-type, Z-type and C-type. The Q-type zinc fingers contain a conserved motif, QALGGH which is mainly plant-specific, however, C-type zinc fingers and Z-type zinc fingers are both in plant and other organisms [39, 45, 46]. The result suggesting three different subclasses have various amino acid sequence motif which may be involved in different feature of plant growth and development’s. For instance, Q-type of C2H2-zinc finger family had clustered in subgroups I, II, IV and V, and only subgroup II contains QALGGH motifs (Q-type) along with LDLDL, FDLDL and IDLDL motifs, which were previously reported to play different functions during plant growth and development [3].

The expression profiles of 386 upland cotton C2H2-zinc finger members, a total of 378 were detected in upland cotton and only 8 C2H2-zinc finger members could not be detected by RNA-Seq data. These C2H2-zinc finger genes exhibited a wide range of expression levels at various developmental stages. Interestingly, the majority of C2H2-zinc finger members belonging to subgroups I, IV and V were expressed relatively at low levels in different tissues, which provides a stronger evidence that many of C2H2-zinc finger genes in these subgroups are not directly involved in cotton fiber development (Additional file 2: Figure S2C). In some members of subgroup II showed high expression during developmental stages of cotton fiber. This result was in agreement with earlier reports which suggested that C2H2-zinc finger genes with QALGGH motifs and LDLDL, FDLDL and IDLDL motifs had a role in response to biotic and abiotic stress [3, 17, 19]. In addition, the AT1G10480 (ZFP5), which is homologs to *Gh_D02G1695* and *Gh_A03G1255*, function in regulating trichome cell development by GA signaling In *Arabidopsis*, [13, 47]. Overexpression of this gene in *Arabidopsis thaliana*, accelerates high levels of trichome initiation [40]. *AT1G67030*, (ZFP6) which is homolog to *Gh_D09G0619* involved in regulating trichome development (initiation) through integrating cytokinin and gibberellin signaling pathways in *Arabidopsis*. These two *Arabidopsis* genes and their homologous genes from upland cotton showed low expression levels in diverse stages of fiber development, suggesting that these genes may play a negative role in controlling fiber development by integrating plant hormones or plant pathways. AT2G41940 (ZFP8) and ZFP5 interaction, facilitates the regulation of epidermal cell differentiation [47]. Upland cotton C2H2-zinc finger genes, *Gh_A05G2741*, *Gh_D05G3769*, *Gh_A04G0449* and *Gh_Sca045498G01* (homologs to AT2G41940) were significantly expressed at initiation stages (0 and 3 DPA) but not in elongation stage of cotton fiber. C2H2-zinc finger genes were differentially expressed in Li1 mutant and wild-type during fiber development. A total of 27 and 45 C2H2-zinc finger genes were down-regulated at 0 and 8 DPA, respectively, while 36 and 51 C2H2-zinc finger genes were up-regulated at the same period of 0 and 8 DPA, respectively, which provide a stronger evidence of the involvement of these genes either positively or negatively in initiation and elongation stages of cotton fiber development of Li1 mutant and the wild-type (Fig. 3). Previously, it was stated that C2H2-zinc finger genes with other transcription factors were differentially expressed in cotton fuzz fiber mutants during initiation and elongation stages [48]. In the Ligon-lintless-2 mutant, C2H2-zinc finger gene was indicated as a candidate gene in regulating cotton fiber development [23].. By contrast, the C2H2-zinc finger genes belonging to subgroups III, VI and VII (Z-type and C-type) were significantly expressed in cotton fiber and tissues as compared to Q-type, which pointed that, they may be involved in regulation of cotton fiber development. The previous reports showed that some C2H2-zinc finger genes were up-regulated in wild-type and down-regulated in Ligon-lintless-1 mutant at 5 and 7 DPA of cotton fiber development [22]. The expression level of C2H2-zinc finger genes were not be detected in Ligon-lintless-2 but significantly expressed in wild-type at 16 DPA [23]. In the Ligon-lintless-2 mutant, C2H2-zinc finger gene was indicated as a candidate gene in regulating cotton fiber development [23] . Furthermore, some C2H2-zinc finger genes could be associated with short fiber development in Ligon-lintless-1. The findings of this work may help to elucidate the possible roles of C2H2-zinc finger genes in fiber development and will lay the foundations for further molecular and functional analysis of C2H2-zinc finger genes in cotton.

## Conclusion

The C2H2-zinc finger gene family is one of the most of the abundant transcription factor families in higher plants and plays a key role in plant growth and development. This research is the first comprehensive analysis of C2H2-zinc finger genes and their expression analysis in cotton fiber development. A total of 386, 196 and 195 C2H2-zinc finger genes were found to be in upland cotton, *G. arboreum* and *G. raimondii,* respectively. The result showed that C2H2-zinc finger gene members were distributed across the whole cotton genome. Based on phylogenetic tree analysis, these C2H2-zinc finger gene members were divided into 7 subgroups. C2H2-zinc finger proteins within the same subgroup contained similar exon-intron structure and protein motif compositions. Moreover, RNA-Seq data showed that at least some of C2H2-zinc finger genes were involved in diverse functions during cotton fiber development while the functions of the most upland cotton C2H2-zinc finger genes remain unclear, further research is therefore needed to determine a specific function of this group of most abundant transcription factors within the plant kingdom. The expression profiles of 16 genes during cotton fiber development, through RT-qPCR, showed that different C2H2-zinc finger genes are either positively or negatively involved in regulation of fiber development in cotton. Based on our findings, the expression levels of C2H2-zinc finger genes family is a pointer of their involvement in various biochemical and physiological functions which are directly related to cotton fiber development during initiation and elongation stages. Further research on functional studies of this family of C2H2-zinc finger proteins is necessary in order to understand their interactions and regulations of various pathways in stimulating cotton fiber development. The results of this research provide the fundamental information for further investigations on the roles of C2H2-zinc finger genes in cotton fiber development and will be useful for further study on the evolutionary time of C2H2-zinc finger genes in other plant species.

## Methods

### Identification of C2H2-zinc finger gene family in cotton

The upland cotton and *G. raimondii* (D_5_) genome sequence were extracted from the Cotton Gen database (http://www.cottongen.org); *G. arboreum* (A_2_) genome sequence was downloaded from the Cotton Genome Project (http://cgp.genomics.org.cn/page/species/download). The Hidden Markov Model (HMM) profile of the C2H2-zinc finger domain (PF00096) was downloaded from Pfam database (http://pfam.sanger.ac.uk/) and was used to identify the C2H2-zinc finger genes in the cotton genome (proteome sequence) using HMMER 3.0 software [49] with E value < 10^− 10^. Moreover, the corresponding protein sequences of C2H2-zinc finger proteins were downloaded from Arabidopsis database (TAIR; http://www.Arabidopsis.org/), *T. cacao*, *Z. mays*, *V. vinifera* and *P. trichocarpa* protein sequences were obtained from the plant transcription factor database (http://planttfdb.cbi.edu.cn/). Hence, they were utilized as query sequences to identify all the cotton C2H2-zinc finger proteins encoded by searching also against cotton proteome sequences, followed by removal of the same sequences from all the search results. Furthermore, to confirm the protein sequences derived from the selected cotton C2H2-zinc finger, candidate genes were examined using the domain analysis SMART (http://smart.emblheidelberg.de/). Only the protein sequences with C2H2-zinc finger domains were taken for further analyses. All redundant sequences were manually removed, resulting in 386 protein sequences in *G. hirsutum* contained C2H2-zinc finger domain. Moreover, the isoelectric points (pI) and molecular weights of upland cotton C2H2-zinc finger proteins were estimated by ExPASy Server tool (http://web.expasy.org/compute_pi/). In addition, WoLFPSORT (http://wolfpsort.org/) was used to predict the subcellular localization of the upland cotton C2H2-zinc finger proteins.

### Phylogenetic analysis of upland C2H2-zinc finger proteins

Multiple sequence alignments of upland cotton, *G. arboreum, G. raimondii, T. cacao*, *Z. mays*, *V. vinifera*, *P. trichocarpa* and *A. thaliana* C2H2-zinc finger proteins were performed using ClustalW (http://www.clustal.org/clustal2/). Phylogenetic tree was constructed with MEGA 6.0 software (http://www.mega software.net/) using the neighbor-joining (NJ) algorithm with 1000 bootstrap repetitions. The tree was constructed with the following parameters: Substitution, Poisson Model; data subset to use, the p-distance, pairwise deletion; replication, bootstrap analysis with 1000 replicates. In addition, minimum evolution method was also used to validate the result of the NJ method. A separate phylogenetic tree was constructed with all the upland cotton C2H2-zinc finger proteins for further analysis.

### Exon-intron structure analysis and conserved motif identification

The Gene structure display server (GSDS 2.0, http://gsds.cbi.pku.edu.cn/index.php) [50] was used to perform the exon/intron structure. The Multiple Expectation Maximization for Motif Elucidation (MEME) system (Version 4.9.1, http://meme.nbcr.net/meme/) [51] was used to find conserved motifs for each upland cotton C2H2-zinc finger proteins. The following parameters were used: “any number of repetitions, the maximum number of motifs-20, and optimum width from 6 to 250”.

### Chromosomal localization and gene duplication

C2H2-zinc finger genes were located on cotton chromosomes based on their location data retrieved from the cotton genome sequences. The distribution of genes on the cotton chromosomes was mapped by Map Chart software. The duplicated genes events were identified based on the criteria defined in the previous study, which stated that the aligned region of two sequences covers > 80% of the longer sequence and the similarity of the aligned region is > 70% [52]. The DnaSP software (version 5.10) [53] was used to estimate non-synonymous substitution rate (Ka) and synonymous substitution rate (Ks), which was used to calculate the date of duplication events with the eq. T = Ks/2λ, supposing clock-like rate (λ) of 1.5× 10^− 6^ (Mya) synonymous substitution rate per 10^− 8^ years for cotton [54].

### Plant materials, RNA extraction and RT-qPCR analysis

Two Upland cotton (*G. hirsutum* L.), were used in this study, Ligon-lintless-1 (*Li1*) which is a mutant form and its wild-type (TM-1) were planted in the experimental field at the Institute of Cotton Research, Chinese Academy of Agricultural Sciences (ICR, CAAS) under normal field conditions. The Ligon-lintless-1 (*Li1*) is a mutant upland cotton, with abnormal morphological characteristics such as distorted leaves, stems and significantly reduced or short lint fiber length approximately 4 to 6 mm on the mature seed [55]. At flowering stage, the flowers were tagged for self-pollination before anthesis in the experimental site. To test the C2H2- zinc finger gene expression, samples were harvested from Li1 and wild-type (TM-1) at 0, 5, 8 and 10 days post anthesis (DPA). Cotton fibers are unicellular, branched, simple trichomes (or seed hairs) which do differentiate from ~ 25% of the epidermal cells in the outer integument of a developing seed [26]. The first visible signs of cotton fiber development is evident on the day of flower opening (anthesis) [56]. RNA was isolated from ovules and fibers using the RNA Aprep Pure Plant Kit (Tiangen Biotech, Beijing, China). Gel electrophoresis and a NanoDrop 2000 spectrophotometer developed by the Thermo Fisher, Waltham, MA, USA were used to determine the quality and concentration of RNA sample. RT-qPCR analysis was conducted using the Applied Biosystems 7500 Real-Time PCR system and the SYBER premix ExTaq kit (TaKaRa Bio Inc., Nojihigashi 7–4-38, Kisatsu, Shiga 525–0058, Japan). The amplification of the target genes was examined by a SYBR Green fluorescence signal. *G. hirsutum* β-actin gene (GenBank accession no: AY305733) was used as the reference gene and primers specific to 16 C2H2-zinc finger genes were used for RT-qPCR analysis based on their expression levels at initiation and elongation stages of cotton fiber development. The detailed information of RT-qPCR procedure and gene expression analysis was done as described by Salih et al. 2016 [44].

### Gene expression analysis using the publicly available RNA-seq data

RNA-sequencing (RNA-seq) data from various tissues of wild-type (TM-1) (*G. hirsutum*) were downloaded from the database in National Center for Biotechnology Information website (http://www.ncbi.nlm.nih.gov/) under accession number (PRJNA248163) [27]. To estimate the gene expression level at different developmental stages, including root, stem, leaves and fibers at 0, 3, 5, 10, 20 and 25 DPA, we calculated the expression of each gene using FPKM (Fragments per Kilobase of exon model per Million mapped reads) with Cufflinks (Version 2.1.1) [57] (http://cufflinks.cbcb.umd.edu/). Heat maps were generated and hierarchical clustering was performed using MeV_4_9_0 software [58]. In addition, RNA-seq experiment, including two stages of cotton fiber development (0 and 8 DPA) from Ligon-lintless-1 mutant wild-type (TM − 1) upland cotton was performed by Illumina sequencing. RNA-seq data were mapped to the entire genome of *G. hirsutum* (TM-1) [27] using Tophat2 (v2.0.9) software [59]. Cufflinks (v2.1.1) software [57] was used to reconstruct the cotton transcriptome, followed by transcript abundance assembly, and differential isoform analysis. To calculate the gene expression level at different developmental stages of cotton fiber development at 0 and 8 DPA. Gene FPKMs were computed by summing the FPKMs of transcripts in each gene group. Fold changes of different genes expression analysis and the related statistical computations of the two tested conditions were performed using the DESeq R package (1.10.1) [60]. The resulting *P*-values were adjusted using Benjamini’s and Hochberg’s method to control the false rate [61]. Only genes with an adjusted *P*-value ≤0.05 found using DESeq were categorized as differentially expressed.

## Additional files

Additional file 1: **Figure S1.** Distribution of C2H2-zinc finger genes on cotton chromosomes. The chromosomal position of each C2H2-zinc finger gene was located to the *G. hirsutum* genome. (PDF 96 kb)

Additional file 2: **Figure S2.** Phylogenetic tree analysis, gene structure and conserved motifs of upland cotton C2H2-zinc finger genes were performed. (**A**) An unrooted tree is constructed by MEGA6.0 software using the full length amino acid sequences of the 386 upland cotton C2H2-zinc finger proteins by the Neighbor-Joining method, with 1000 bootstrap replicates. Based on phylogenetic tree, upland cotton C2H2-zinc finger divided into seven main subgroups (group I to VII) and each subgroup indicated with different colors and (**B**) Exon-intron structure of upland cotton C2H2-zinc finger genes. The yellow boxes represent exons, black lines represent introns and blue boxes represent the upstream/downstream (UTRs). The sizes of exons and introns can be estimated using the scale at the bottom. (**C**) Expression patterns of upland cotton C2H2-zinc finger members in different tissues (root, stem and leaves) and different of fiber developmental stages (0, 3, 5, 10, 20 and 25 DPA). The expression levels are represented by the color bar. (**D**) Distribution of conserved motifs in upland cotton C2H2-zinc finger members and different motif boxes present by different colors (motifs 1 to 20). (TIFF 6685 kb)

Additional file 3: **Figure S3.** The neighbor-joining (NJ) and minimum evolution methods of C2H2-zinc finger proteins family in *G. hirsutum*. (TIFF 3125 kb)

Additional file 4: **Figure S4.** Motif analysis of C2H2-zinc finger gene family in upland cotton. A total of 20 putative conserved motifs of upland cotton TPS proteins were identified using the MEME online program. (PDF 300 kb)

Additional file 5: **Table S1.** Location, annotation and protein domains of C2H2-zinc finger genes in cotton genomes. (XLSX 54 kb)

Additional file 6: **Table S2.** List of 386 C2HC2-zinc finger genes in upland cotton and their proteins sequence features including, proteins length, molecular weight and subcellular positions. (XLSX 23 kb)

Additional file 7: **Table S3.** Synonymous substitution rates (Ks) and non-synonymous substitution rates (Ka) are represented for each orthologous gene pairs between upland cotton and their ancestral diploid cotton, *G. arboreum (*A_2_) and *G. raimondii* (D_5_). (XLSX 35 kb)

Additional file 8: **Table S4.** Multiple sequence alignment of upland cotton C2H2-zinc finger proteins for each group. (PDF 494 kb)

Additional file 9: **Table S5.** Expression patterns of C2H2-zinc finger genes at various developmental stages in *G. hirsutum* (TM-1) were measured by RNA-seq. (XLSX 49 kb)

Additional file 10: **Table S6.** Expression patterns of C2H2-zinc finger genes at two stages of cotton fiber development in Ligon-lintless-1 and wild-type (WT) were measured by RNA-seq. (XLSX 38 kb)

## Data Availability

All related datasets supporting the results of this study are available within the manuscript and its supplementary files.

## Abbreviations

DPA: Days post anthesis
Dt: Tetraploid A
Dt: Tetraploid D
Ka: Non-synonymous substitution rates
Ks: Synonymous substitution rates
Li1: Ligon-lintless-1
MYA: Million par years
TFs: Transcription factors
