# Supplementary figures and images for: Genome-wide analysis of cotton C2H2-zinc finger transcription factor family and their expression analysis during fiber development
Source: BMC Plant Biol. 2019 Sep 11;19:400. doi: 10.1186/s12870-019-2003-8 (PMC6739942; doi:10.1186/s12870-019-2003-8)

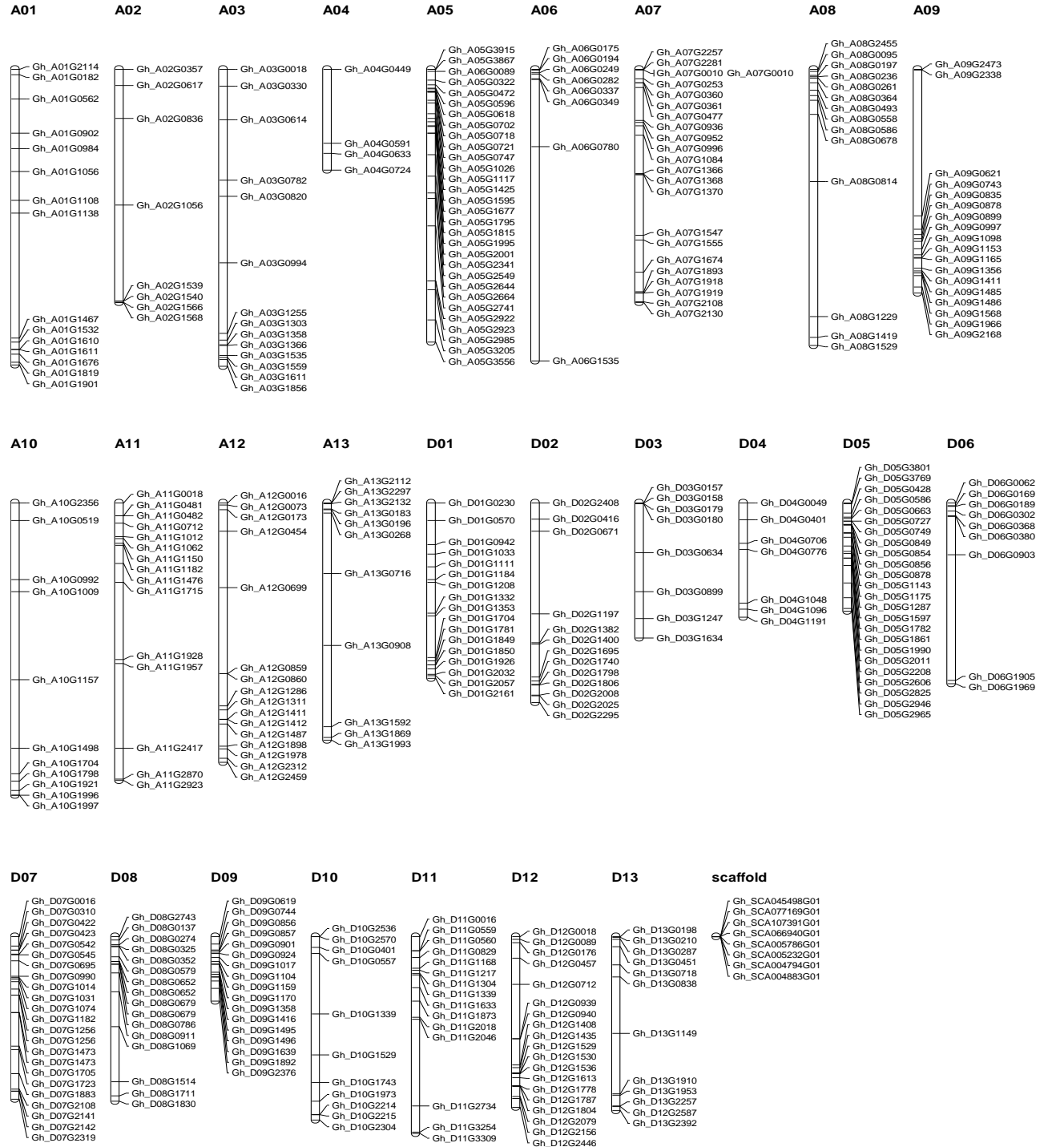

Supplement: Supplementary file 1 — Figure S1. Distribution of C2H2-zinc finger genes on cotton chromosomes. The chromosomal position of each C2H2-zinc finger gene was located to the G. hirsutum genome. (PDF 96 kb) [file 12870_2019_2003_MOESM1_ESM.pdf]

## Conserved motif identification

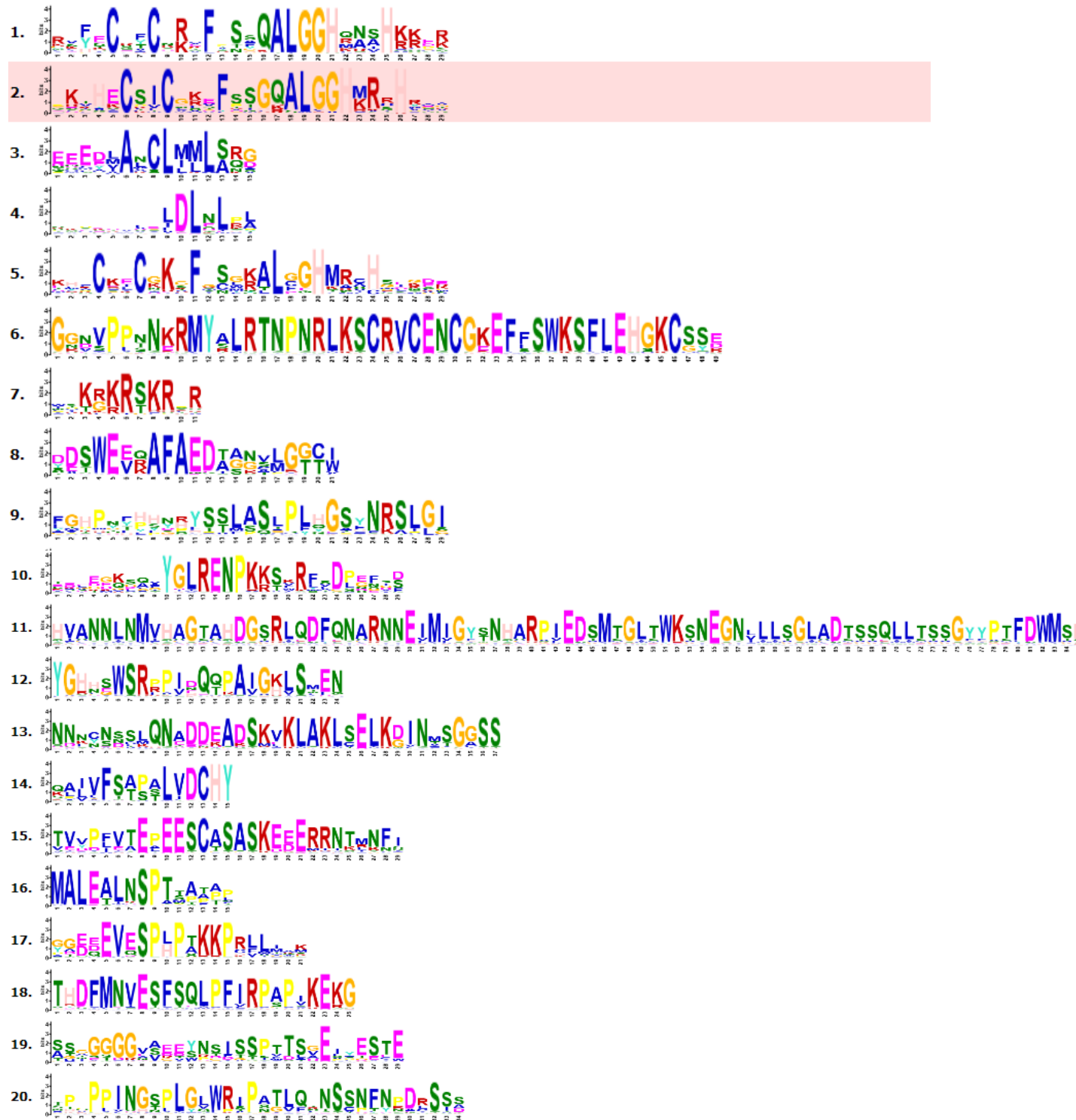

Supplement: Supplementary file 4 — Figure S4. Motif analysis of C2H2-zinc finger gene family in upland cotton. A total of 20 putative conserved motifs of upland cotton TPS proteins were identified using the MEME online program. (PDF 300 kb) [file 12870_2019_2003_MOESM4_ESM.pdf]
