# Supplementary material for: Genome-wide analysis of cotton C2H2-zinc finger transcription factor family and their expression analysis during fiber development
Source: BMC Plant Biol. 2019 Sep 11;19:400. doi: 10.1186/s12870-019-2003-8 (PMC6739942; doi:10.1186/s12870-019-2003-8)
Supplement: Supplementary file 8 — Table S4. Multiple sequence alignment of upland cotton C2H2-zinc finger proteins for each group. (PDF 494 kb) [file 12870_2019_2003_MOESM8_ESM.pdf]

Subgroup 1

```
Gh_D03G1634       WE--------------VKAFAE------E--TSN--AMGTTWPPRSYTCTFCRREFRSAQ
Gh_A03G0018       WE--------------VKAFAE------D--TSN--AMGTTWPPRSYTCTFCRREFRSAQ
Gh_D12G2079       WE--------------VRAFAE------D--IGN--VLGTTWPPRSYTCSFCTREFRSAQ
Gh_A12G1898       WE--------------VRAFAE------D--IGN--VLGTTWPPRSYTCSFCAREFRSAQ
Gh_D08G1711       WE--------------VRAFAQ------D--TGN--VMGTTWPPRSYTCTFCRREFRSAQ
Gh_A08G1419       WE--------------VRAFAQ------D--TGN--VMGTTWPPRSYTCTFCRREFRSAQ
Gh_A09G2338       WE--------------VRAFAE------D--TGN--VMGTTWPPRSYSCSFCRREFRSAQ
Gh_D09G1892       WE--------------VRAFAE------D--TGN--VMGTTWPPRSYSCSFCRREFRSAQ
Gh_A13G0183       WE--------------EKAFAE------D--AAG-SLEGCVWPPRSYTCSFCRREFKSAQ
Gh_D13G0198       WE--------------EKAFAE------D--AAG-SLGGCIWPPRSYTCSFCWREFKSAQ
Gh_D10G2304       WE--------------EQAFAE------D--AAR-CLRGCIWPPRSYSCSFCNREFRSAQ
Gh_A10G1997       WE--------------EQAFAE------D--AAR-CLRGCIWPPRSYSCSFCNREFRSAQ
Gh_A10G1996       WE--------------EQAFAE------D--AAR-CFRGCIWPPRSYSCSFCNREFKSAQ
Gh_A04G0724       WE--------------EQAFAE------D--TAG-SLGGCIWPPRSYSCSFCGREFTSAQ
Gh_D04G1191       WE--------------EQAFAE------D--TAG-SLGGCIWPPRSYSCSFCGREFTSAQ
Gh_D11G3309       WE--------------EQAFAE------D--ASG-TPGGCIWPPRSYSCSFCGREFRSAQ
Gh_A11G2923       WE--------------EQAFAE------D--ASG-TLGGCIWPPRSYSCSFCGREFRSAQ
Gh_D07G2141       WEF-----------NRLDLVEL------E--GEQYSCCKSPWPPKYYTCAFCKREFRSAQ
Gh_A07G1918       WEF-----------NRLDLVEL------E--GEQYSCCKSPWPAKYYTCTFCKREFRSAQ
Gh_A09G1966       WRE-----------F-------------G--EEDYVCGGIPWPPRSYTCSFCKREFRSAQ
Gh_D02G0416       --M-----------ATNQGF-----------EEDYL-SGFSWPPRSYTCSFCERGFRSAQ
Gh_A02G0357       --M-----------ATNQGF-----------EEDYL-SGFSWPPRSYTCSFCERGFRSAQ
Gh_A01G0182       WSS-----------CNNQGFGS------G--GEDYL-SGFSWPPRSYTCSFCKREFRSAQ
Gh_D01G0230       WSS-----------CNNQGFGS------G---EDYL-SGFSWPPRSYTCSFCKREFRSAQ
Gh_A10G1009       WSE-----------ESDD--------------DQVKCHTEATTKRSYECIFCKRGFTNAQ
Gh_D10G1529       SSE-----------ENDD--------------DQVKCHTGATTKRSYECTFCKRGFTNAQ
Gh_A08G0558       SSE-----------ETDK--------------SEQSIDDMRTGRRSYECVFCKRGFSTAQ
Gh_D08G0652       SSE-----------ETDK--------------SEQSIDDMRAGRRSYECVFCKRGFTTAQ
Gh_A07G1919       SSE-----------ETDR--------------SEQSNDDTGTG-RSYECVFCKRGFTTAQ
Gh_D07G2142       SSE-----------DTDR--------------SEQSNDDTGTG-RSYECVFCKRGFTTAQ
Gh_Sca107391G01   SSE-----------DTDR--------------SEQSNDDTGTG-RSYECVFCKRGFTTAQ
Gh_D11G1339       SSE-----------ESDR--------------SEQSNDDVGSG-RSYECVFCKRGFTTAQ
Gh_A11G1182       SSE-----------ETDR--------------SEQSNDDVGSG-RSYECVFCKRGFTTAQ
Gh_A07G2257       -----------------------------------SDEQGASPARSYDCTFCKRGFSNAQ
Gh_D07G0990       -----------------------------------SDEQGASPARSYDCTFCKRGFSNAQ
Gh_D01G2161       WSL--------------------------------EEDDVSIHSKSYTCSFCKKGFLNAQ
Gh_A01G1901       WSL--------------------------------EEEDVSVHSKSYTCSFCKKGFSNAQ
Gh_D02G1695       GDSMKVCGEGDESVNSS---------NTISSTAKEKSSMVEADDKKFECQYCFKEFANSQ
Gh_A03G1255       GDSMKVCGEGDESVNSS---------NTISSTAKEKSSMVEADDKKFECQYCFKEFANSQ
Gh_A12G1311       GNFLKGCVEGDESINSS---------SRAAGVD----DGDSTDEKKFECRYCFKEFANSQ
Gh_D12G1435       GNFLKGCVEGDESINSS---------SRAAGVD----DGDSTDEKKFECRYCFKEFANSQ
Gh_A03G1303       LESSTT---KDP-----------------SSGSSEPGGFPAPGDRKYECQYCCREFANSQ
Gh_D02G1740       LESSTT---KDP-----------------SSGSSEPGGFPAPGDRKYECQYCCREFANSQ
Gh_Sca066940G01   LESSTT---KDP-----------------SSGSSEPGGFPAPGDRKYECQYCCREFANSQ
Gh_D07G2108       G-----------------------------SNKAPAAGGSPELDRKYECQYCCREFANSQ
Gh_A07G1893       G-----------------------------SNKAPPAGGSLELDRKYECQYCCREFANSQ
Gh_A08G0364       SVKA---------------------------------EEDFGESSKFECPFCQRLFANSQ
Gh_D08G2743       SVKA---------------------------------EEDFGDSRKFECPFCHRVFANSQ
Gh_D07G1256       VSDK---------------------------------SDDFGESRKFECPFCHRVFGNSQ
Gh_D11G1168       VLEK---------------------------------AKGFGESRKFECPFCHRVFANSQ
Gh_A11G1012       VLEK---------------------------------AEGFGESRKFECPFCHRVFANSQ
Gh_D09G0619       PPSV---------------------------------RRNVSEYRRFECQFCHRGFANSQ
Gh_A09G0621       PPSV---------------------------------QRNVSEYRRFECQFCHRGFANSQ
Gh_A10G1498       ----------------------------------------PCDNRRFECQYCHRGFPNSQ
Gh_D10G1743       ----------------------------------------PCDNRRFECQYCHRGFPNSQ
Gh_A01G1611       -----------SSAET----------SNPITPSV-SSETSQEITHPFVCYRCPKRFTSTY
Gh_D01G1849       -----------LAPET----------SNPITPSV-SIETSQEITHPFVCYRCPKRFTSTY
Gh_D01G1208       -----------TEATESD----NNNNNEDTTKDNENGDNNNNSSRRFECHYCCRNFLTSQ
Gh_A01G1138       -----------TEATESD----NNNNNEDTTKDNENGDNNNNSSRRFECHYCCRNFLTSQ
Gh_A04G0449       ----------ATPSNESD----SAENNEDTTKENEN---NGDNSRRFECHYCCRNFPTSQ
Gh_Sca045498G01   ----------ATPSNESD----SAENNEDTTKENEN---NGDNSRRFECHYCCRNFPTSQ
Gh_A05G2741       ---------SAMARNESD----SAGNNDDTTKEN-E---NGENSRRFECHYCCRNFPTSQ
```

```
Gh_D05G3769          ---------SAIARNESD----SAGNNEDTTKEN-E---NGENSRRFECHYCCRNFPTSQ
Gh_A06G0194          YEGNS-----MEWPDLSLGKHG------DIAASKSGLRSNYASNKIFSCNFCRRKFYSSQ
Gh_D06G0189          YEGNS-----MEWLNLSLGRHR------DIVASKSGLRSNYASNKIFSCNFCRRKFYSSQ
Gh_D05G1990          DGDNS-----TEWLNLSLGRHG------DIVAPKSGFQSKYVPNKVFSCNFCRRKFCSSQ
Gh_A05G1795          DRDNS-----TEWLNLSLGRHG------DIVAPKGGFQSKYVPNKVFFCNFCRRKFCSSQ
Gh_A06G1535          SNDNRSLRESEIVINLSTPTNPII--------TSSE--NQPEPEKTFSCNYCRSKFTSPQ
Gh_D06G1905          SNDNRSSRESEIGLNLSTTMNPII--------TSSE--NQPEPERTFSCNYCRSKFTSPQ
Gh_D10G1973          NGGAKLRRESKIELDLFTPRNPTN--------SSQHDGDHHDKSKVFSCNFCKRVFSTSQ
Gh_A10G1704          NGGAKPRRESKIELDLFTPRSPTN--------SSQHDGDHHDKSKVFSCNFCNRVFSTSQ
Gh_D13G1149          ---SV-------ELRL----SS--------MISSPKPQQQSSKGRAFTCGFCSKQFSTSQ
Gh_A13G0908          ---SV-------ELRL----SS--------MRSSPQPQQQSSKSRAFTCGFCSKQFSTSQ
Gh_A07G2130          YNFFN-------QP------EPS-SASKPIKKHPQ-TASPSAPPRLFPCLYCPRKFYTSQ
Gh_D04G0706          YNFFN-------QP--------S-SASRPTKKHPQ-RPSPSAPPRLFQCLYCPRKFYTSQ
Gh_A05G2922          YNFFN-------QP--------S-SASRPTKKHPQ-RPSPSAPPRLFQCLYCPRKFYTSQ
Gh_A05G2923          YNFFN-------QP--------S-SASRPTKKHPQ-RPSPSAPPRLFQCLYCPRKFYTSQ
Gh_D13G0287          KG---------------A-------------TGETMGINTLSTPRVFSCNYCRRKFYSSQ
Gh_A13G0268          KG---------------A-------------TGETMGTNSLSTPRVFSCNYCRRKFYSSQ
Gh_D07G1705          VG---------------LSFTST---SESSNIDPALRTTASAIPRVFSCNYCQRKFFSSQ
Gh_A07G1547          VG---------------LSFTST---SESSNIDPALRTTASAIPRVFSCNYCHRKFFSSQ
Gh_D08G0274          ---ND-------QLNLDLALEPSSS-------SSSSNSPTTTEPRVFSCNYCQRKFFSSQ
Gh_A08G0197          ---ND-------QLNLDLALEPSSS-------SSSSNSPTTTEPRVFSCNYCQRKFFSSQ
Gh_D08G0911          ---NN-------QPNLELVLEPSSS-------SSSPHIP--AAPRIFSCNYCKRKFYSSQ
Gh_A08G0814          ---NN-------QPNLELVLEPSSS-------SLSPHSP--AAPRIFSCNYCKRKFYSSQ
Gh_D06G1969          ---KP-------ELNLIECFKVTVS-SDDHQDCLGTPQGNETEPRVFSCNYCQRKFYSSQ
Gh_A06G1920          ---KP-------ELNLIECFKVTVS-SDNHQDCLGTPQGNETEPRVFSCNYCQRKFYSSQ
Gh_D05G1143          ---NP-------ELNFIDCFKADLS-GDQDS-SELPPQGNETEPRVFSCNYCQRKFYSSQ
Gh_A05G1026          ---NP-------ELNFIDCFKADLS-GDQDS-SELPPQGNETEPRVFSCNYCQRKFYSSQ
Gh_D07G0310          FNFNQ-------ELNLIGSLNT-----------PETPQPADSEQRVFSCNYCQRKFYSSQ
Gh_A07G0253          FNFNQ-------ELNLIGSLNT-----------PETPQPADSEQRVFSCNYCQRKFYSSQ
Gh_D13G1953          FN-QE-------ELNLIDSLKNSTGSSD-----STTTQPTDGEQRVFSCNYCQRKFYSSQ
Gh_A13G1592          LN-QE-------ALNLIDSLKNSTGSSD-----STTTQPTDGEQRVFSCNYCQRKFYSSQ
Gh_D05G0586          GGFNK-------ELNLIDSFNTA-GSSDSTAPEPETPQATDGEQRVFSCTYCQRKFYSSQ
Gh_A05G0472          GGFNK-------ELNLIDSLNAA-GSSDSTAPEPETPQATDGEQRVFSCTYCQRKFYSSQ
                                                                   : *  *   * .

Gh_D03G1634          ALGGHMNVHRRDRARLHQTQPPPHGGDATT--GPL-------I-----------------
Gh_A03G0018          ALGGHMNVHRRDRARLHQTQPLPHGGDATT--VPL-------I-----------------
Gh_D12G2079          ALGGHMNVHRRDRARLRQTYPAT----------------------SSILSATAISSS--N
Gh_A12G1898          ALGGHMDVHRRDRARLHQTYPATDALRDPA--------------TSSILSATAISSS--T
Gh_D08G1711          ALGGHMNVHRRDRARLHQTHPPAPTVPL--------------------------INQ--T
Gh_A08G1419          ALGGHMNVHRRDRARLHQTHPPAPTVPL--------------------------INQ--T
Gh_A09G2338          ALGGHMNVHRRDRARLHQAHPAAGGGGGGN--GAL-------NNPTSPSSPSTTSSS--T
Gh_D09G1892          ALGGHMNVHHRDRARLHQAHPAAAA--GGN--GAL-------NNPTSPSSPSTTSSS--T
Gh_A13G0183          ALGGHMNVHRRDRAKLKQSLSPKNEVVSNQNQN---------------------------
Gh_D13G0198          ALGGHMNVHRRDRAKLKQSLSPKNEVVSNQNQN---------------------------
Gh_D10G2304          ALGGHMNVHRRDRAKLKQSVNDHHQYPNNEEAA---------------------------
Gh_A10G1997          ALGGHMNVHRRDRARLKQSLHGHHQYPNNEAAA---------------------------
Gh_A10G1996          ALGGHMNVHKKDRAKLKQLDHSIDDHKGQ-SFG---------------------------
Gh_A04G0724          ALGGHMNVHRRDRARLKQSPTSHDEILHHDPQNSP-------NHIQKPFL----------
Gh_D04G1191          ALGGHMNVHRRDRARLKQSPTSQDGILHHDPQNSP-------NHIQKPFL----------
Gh_D11G3309          ALGGHMNVHRRDRARLKQSPPSPSPP-HHEHHHHQ-------NNIQDPFL----------
Gh_A11G2923          ALGGHMNVHRRDRARLKQSPPSPSPP-HHEHHQHQ-------NNIQDPFL----------
Gh_D07G2141          ALGGHMNVHRKDRARLRLFSSWVLESQNPNK-----------------PNS----------
Gh_A07G1918          ALGGHMNVHRKDRARLRLFSSWVLESQNPNK-----------------PSS----------
Gh_A09G1966          ALGGHMNVHRRETARLLRQSSTPPG----DYNG------------QPSFL----------
Gh_D02G0416          ALGGHMNVHRRDKARLRQLPPPM------DHHG------------QSPFL----------
Gh_A02G0357          ALGGHMNVHRRDKARLRQLPPPM------DHHG------------QSPFL----------
Gh_A01G0182          ALGGHMNVHRRDRARLRQSPPPPPKDHHHHHG------------QPPFL----------
Gh_D01G0230          ALGGHMNVHRRDRARLRQSPPPPPKDHHHHHG------------QPPFS----------
Gh_A10G1009          ALGGHMNIHRKDRVKAKQPTSSAVLSMNPRYAPTI----PTYAPSRYHQIVEAQIQT---
Gh_D10G1529          ALGGHMNIHRKDRAKAKQPTSSAVLSMNPRYAPTI----PTYEPSRHHPIVEAQTQT---
Gh_A08G0558          ALGGHMNIHRKDRVK-SRPSSVPIVSLSGNKADD-----KNY-PSFRPYSYPP-IQS---
Gh_D08G0652          ALGGHMNIHRKDRAK-CRPSSVPIVSLSGNKADD-----KNY-PSFRPYSYPP-IRS---
```

```
Gh_A07G1919          ALGGHMNIHRKDRAKGSRPSSVPIVSDKFVDVDDH----DNYNPRISSYPYPP-IQT---
Gh_D07G2142          ALGGHMNIHRKDRAKSSRPSSVPIVSDKFVDVDDH----DNYNPRISSYPYPP-IQT---
Gh_Sca107391G01      ALGGHMNIHRKDRAKSSRPSSVPIVSDKFVDVDDH----DNYNPRISSYPYPP-IQT---
Gh_D11G1339          ALGGHMNIHRKDRAKSSRPNSVPIVSGKVDDE--------NYASLRSSSSYLPIIQS---
Gh_A11G1182          ALGGHMNIHRKDRAKSSRPNSVPIVSGKLDDE--------NYASLRSSSSYLPIIQS---
Gh_A07G2257          ALGGHMNIHRRDKAKLKQPSPTHETT-ST---------------QQSNFDISKIIPS---
Gh_D07G0990          ALGGHMNIHRRDKAKLKQPSPTRETTATT---------------QQSNFDISTIIPS---
Gh_D01G2161          ALGGHMNIHRKDRAKLRESFEENVLISSVI-TLG-------LVQVQDRYLISETILE---
Gh_A01G1901          ALGGHMNIHRKDRAKLRESFEENVLSSDITLDRV-------QVQVQDRYLISEIILE---
Gh_D02G1695          ALGGHQNAHKKERMKKKRLQLQAKRA-SLN---------CYLQPFQNSLG----------
Gh_A03G1255          ALGGHQNAHKKERMKKKRLQLQAKRA-SLN---------CYLQPFQNSLG----------
Gh_A12G1311          ALGGHQNAHKKERMKKKRLQLQAKRAASIN---------SYLQPLHDSCY----------
Gh_D12G1435          ALGGHQNAHKKERMKKKRLQLQAKRAASIN---------SYLQPLHDSCY----------
Gh_A03G1303          ALGGHQNAHKKERQQLKRAQLQATRSAAVS---------FARNPIISAFAPPPHLLA---
Gh_D02G1740          ALGGHQNAHKKERQQLKRAQLQATRSAAVS---------FARNPIISAFAPPPHLLA---
Gh_Sca066940G01      ALGGHQNAHKKERQQLKRAQLQATRSAA--------------------------------
Gh_D07G2108          ALGGHQNAHKKERQLLKRAQMQAAA---------------TRNSMISPFTSPPHLLA---
Gh_A07G1893          ALGGHQNAHKKERQLLKRAQMQAAA---------------TRNSMISPFTPPPHLLA---
Gh_A08G0364          ALGGHQNAHKRERQRARRTQFLHT--HHHH----------HYQRYIAAAA-----AA---
Gh_D08G2743          ALGGHQNAHKRERQRARRTQFLHT--HHHH----------HYQRYIAAAA-----AA---
Gh_D07G1256          ALGGHQNAHKRERQKARQAQFHS------------------RQRFI----------A---
Gh_D11G1168          ALGGHQNAHKRERQKARRAQFHS------------------QQRLI----------A---
Gh_A11G1012          ALGGHQNAHKRERQKARRAQFHS------------------QQRLI----------A---
Gh_D09G0619          ALGGHQNEHKRERRAKQGSFFSLHHQQQQQ----------LKQRFL--------------
Gh_A09G0621          ALGGHQNAHKRERRAKQGSFFTLHHQQQQQ----------LKQRFL--------------
Gh_A10G1498          ALGGHQNAHKRERRAKLASSFTAHHH----------------QRFV--------------
Gh_D10G1743          ALGGHQNAHKRERQEKPASSFTAHHH----------------QRFL--------------
Gh_A01G1611          ALGGHQNAHKKERNEELRLYNERRLALSRQSTVPADGRAKTKRAL-------AVLTN--Q
Gh_D01G1849          ALGGHQNAHKKERNEELRLYNERRLALSRQSTVPADGRAKTKRAL-------AVLTN--Q
Gh_D01G1208          ALGGHQNAHKRERQHAKRQHNNSFSLS--DAHN---------DIYG---FFNYRLPSPSM
Gh_A01G1138          ALGGHQNAHKRERQHAKRQHNNSFSLS--DAHN---------DIYG---FFNYRLPSPSM
Gh_A04G0449          ALGGHQNAHKRERQHAKRAHLQSAMVHTSLSDA---------HIYG---LVNYRLGSAPT
Gh_Sca045498G01      ALGGHQNAHKRERQHAKRAHLQSAMVHTSLSDA---------HIYG---LVNYRLGSAPT
Gh_A05G2741          ALGGHQNAHKRERQHAKRAHLQSTMVQNSLSDA---------QIYG---FVNYRLASAPT
Gh_D05G3769          ALGGHQNAHKRERQHAKRAHLQSTMVQNSLSDA---------QIYG---LVNYRLASAPT
Gh_A06G0194          ALGGHQNAHKRERGR---CQSERMM-------------------------------P--M
Gh_D06G0189          ALGGHQNAHKRERGR---CQSERMM-------------------------------P--M
Gh_D05G1990          ALGGHQNAHKRERGVVRRYQSERLI-------------------------------A--R
Gh_A05G1795          ALGGHQNAHKRERGVVRRYQSERLI-------------------------------A--R
Gh_A06G1535          ALGGHQNAHRQERALAKRLRE-----------------IQT-QTFTPPTYPHYPYPT--L
Gh_D06G1905          ALGGHQNAHRQERALAKRLRE-----------------IQA-QTFTPPTYPHYPYPT--L
Gh_D10G1973          ALGGHQNAHKQERALAKRRHQGLME------------VNPS-RTFGTPNFPYYPFST--F
Gh_A10G1704          ALGGHQNAHKQERALAKRRHQGLME------------VNPS-RTFGTPNLPYHPFSA--F
Gh_D13G1149          ALGGHQNAHKQERAIAKRRKEMDVGAL---------------PPQQYPF---YSYST--L
Gh_A13G0908          ALGGHQNAHKQERAIAKRRKEMDVGAL---------------ASRQYPF---YSYST--L
Gh_A07G2130          ALGGHQNAHRRERAASRRKFPGGD-----------HDQIRG-AQQ---YNLQQQLNP--F
Gh_D04G0706          ALGGHQNAHKRERAASRRKFPTDQ-----------DQTQQC-TTLHHHHHHHQISPA--F
Gh_A05G2922          ALGGHQNAHKRERAASRRKFPTDQ-----------DQTQQC-TTLH-HHHHHQISPA--F
Gh_A05G2923          ALGGHQNAHKRERAASRRKFPTDQ-----------DQTQQC-TTLH-HHHHHQISPA--F
Gh_D13G0287          ALGGHQNAHKRERTMAKRAMRMGLL--------------------------SDRYTSL--
Gh_A13G0268          ALGGHQNAHKRERTMAKRAMRMGLL--------------------------SDRYTSL--
Gh_D07G1705          ALGGHQNAHKRERTLAKRAMRMGIF--------------------------SDRYASLSL
Gh_A07G1547          ALGGHQNAHKRERTLAKRAMRMGIF--------------------------SDRYASLSL
Gh_D08G0274          ALGGHQNAHKLERTLAKKSRELGSSVQI-------HG-SSL-SGLSHPQRIQSH-----V
Gh_A08G0197          ALGGHQNAHKLERTLAKKSRELSSSVQI-------HG-SSL-SGFSHPQRMQSH-----V
Gh_D08G0911          TLGGHQNAHKLERTLAKKSRELSSIVRA-------HGGSSI-RGSSHDQSMQSP-----I
Gh_A08G0814          ALGGHQNAHKLERTLAKKSRELSSVVRA-------HGGSSI-SGSSHDQRIQSP-----M
Gh_D06G1969          ALGGHQNAHKRERTLAKRGQQRVVVGVGGDGDGGGGGGASF-AALGHPNTHSGRYST--M
Gh_A06G1920          ALGGHQNAHKRERTLAKRGQQRVVVGLGGD--GGGGGGASF-AAFGHPNTHSGRYST--M
Gh_D05G1143          ALGGHQNAHKRERTLAKQGQR----------LGGGAAAASL-SAFGYPNTHPSRYSS--L
Gh_A05G1026          ALGGHQNAHKRERTLAKRGQR----------LGGGAAAASF-SAFGYPNTHPSRYSS--L
Gh_D07G0310          ALGGHQNAHKRERTLAKRAQRIGV-----------HAAFGG-----HPYFHHNHCSS--L
Gh_A07G0253          ALGGHQNAHKRERTLAKRAQRIGV-----------HAAFGG-----HPYFHHNHYSS--L
```

```
Gh_D13G1953          ALGGHQNAHKRERTLAKRGQRMGA-----------HIAAFG-HPYFHHHHHHNHYSS--L
Gh_A13G1592          ALGGHQNAHKRERTLAKRGQRMGT-----------HIAAFG-HPYFHHHHHHNHYSS--L
Gh_D05G0586          ALGGHQNAHKRERTLVKRGQRMGW-----------GGPQAT--ALGHPYFYHNHHSS--L
Gh_A05G0472          ALGGHQNAHKRERTLVKRGQRMGW-----------GGPQAT--AFGHPYFYHNHHSS--L
                     :**** : *: :
```

Subgroup 2

```
Gh_A05G0618          ------HMETRKNKKMKTEDTSM--EFELGGTKSASG-----------------------
Gh_D05G0749          ------HMETRKNKKMKTEDTKM--EFELGGTKSESG-----------------------
Gh_A05G0596          ---------RVYYGRMKTEETSM--ESELGAADNESTVELSGNPVKTRDSPNKS------
Gh_D05G0727          ---------------MKTEETSM--ESEWGAADNESTVELSGNCVKTCDSSNKS------
Gh_D07G0422          G---AAKVHPKRYK-CGTCNRTFRSHQALGGHRA-SH-----KLKSKKISQEEE-EEEDD
Gh_A07G0360          G---AAKVHPKRYK-CGTCNRTFRSHQALGGHRA-SH-----KLKSKKISQEEE--EDDD
Gh_D07G0423          T---AAKAHPKCYK-CETCNITFRSHQALGGHTA-SH-----NFKNKKIAQEEE-----A
Gh_A07G0361          T---EAKAHPKCYK-CETCNRTFRSHQALGGHIA-SH-----NFKNKKIAQE-E-----A
Gh_D12G2156          ---SSKKKIHGKHR-CELCKKTFRSFYTLDEHKR-VC-----SETKKTSKV---------
Gh_A12G1978          ---SSKKKIHGKHR-CELCKKTFRSFYTLDEHKR-VC-----SETKKTSKV---------
Gh_D08G1830          ---SSKKKMNGKHR-CGKCKKAFGSYNTLEEHKR-VC-----LSSA--------------
Gh_A08G1529          ---SSKKKMNGKHR-CGKCKKAFGSYNTLEEHKR-VC-----LSSA--------------
Gh_A08G1229          EFAEFNRTTRGKYR-CETCNKVFNSYQALGGHRA-SH-----KKIKAYE------TELAP
Gh_D08G1514          ESAEFNRTTRGKYR-CETCNKVFNSYQALGGHRA-SH-----KKIKAFE------TELAP
Gh_D12G0939          KSTKPNRSTRGKYK-CETCNRVFKSYQALGGHRA-SH-----KKMKPYSPATEK-IHEPP
Gh_A12G0859          KSTKPNRSTRGKYR-CETCNRVFKSYQALGGHRA-SH-----KKMKPYSPATEA-IHEPP
Gh_A07G1368          KLSKVNRAPRGKYK-CETCNKVFKSYQALGGHRA-SH-----KKMKAYSPPATHNTELEP
Gh_D07G2458          KLSKVNRATRGKYK-CETCNKVFKSYQALGGHRA-SH-----KKMKAYSPPATHDSELEP
Gh_D03G0899          SEFCKDPHKRSKFE-CTTCKKIFHSYQALGGHRA-SH-----KRTKGCSASDNSETSTET
Gh_A03G0614          SEFCKDPHKRSKFE-CTTCKKIFHSYQALGGHRA-SH-----KRTKGCSASDNSETSTET
Gh_D12G1613          -----SPQKGSKYE-CLTCNKTFDSHRALGGHRA-SH-----TKVNGYNESIQESYENNL
Gh_A12G1487          -----SPQKGSKYE-CLTCSKTFDSHRALGGHRA-SH-----TKVNGYNESIQESYENNL
Gh_A05G2644          EVLCNNAPKASKYE-CLTCNKAFDSHRALGGHRA-NH-----TKVNDYNE-------DSL
Gh_D05G2946          EVLCNNAPKASKYE-CLTCNKAFDSHRALGGHRA-NH-----TKVNDYNE-------DSL
Gh_D13G0838          -----SSPKGTKL-----------------------------ESIDGRGE-------DSL
Gh_A13G0716          -----SSPKGTKL-----------------------------ESIHDRGE-------DSL
Gh_D12G0176          SSSAHPLHLNLSYK-CSVCNKAFPSYQALGGHKA-SH-----RKPSTAQNPSITTE----
Gh_A12G0173          SSSAHPLHLNLSYK-CSVCDKAFPSYQALGGHKA-SH-----RKPSTAQNPSITTE----
Gh_D13G0451          SPPPPPPALKLSYK-CSVCDKAFPSYQALGGHKA-SH-----RKPLSADAATTTAAANVD
Gh_A13G2112          SPPPPPPALKLSYK-CSVCDKAFPSYQALGGHKA-SH-----RKPLSADAATTTAAVNVD
Gh_A03G1611          SSAPAPAALKLSYK-CSVCNKAFPSYQALGGHKA-SH-----RKSSTDAKPDNQSI----
Gh_D02G2025          SSAPAPAALKLSYK-CSVCNKAFPSYQALGGHKA-SH-----RKSSTDAKPDNQSI----
Gh_D11G2018          ---AASRTSLNLYK-CTVCNKGFPSYQALGGHKA-SH-----RKLVVGAGDHLTITTAAT
Gh_A11G1957          ---AASRTSLNLYK-CTVCNKGFPSHQALGGHKA-SH-----RKLVVGAGDHLTITTAAT
Gh_D12G0712          ---AAKNSLNHNYK-CEVCNKSFPTYQSLRGHKS-SH-----RKFVGADEDPTTTAAAED
Gh_A12G0699          ---AAKNSLNHNYK-CEVCNKSFPTYQSLGGHKS-SH-----RKFVGADEDSTTTAAAED
Gh_A02G0836          ------TDQQLRFK-CKVCNKAFNSYQALGGHKA-SH-----RKLSSGINDDQSTSTTTS
Gh_A01G0984          ------------------------------------------------N-DDHSTPTTLS
Gh_D01G1033          -----THQKLGTYK-CSVCNKAFNSYQALGGHKA-SH-----RKLSGAN-DDHSTPTTSS
Gh_D06G2303          ----STEEQKPSYK-CSVCNKAFNSYQALGGHKA-SH-----RKLSGGN-DDQTTSAT--
Gh_D05G2011          APAPTSTVQNVSYK-CSVCNKSFNSYQALGGHKA-SH-----RKLSGGN-DDQSTSTTTS
Gh_A05G1815          APAPTSTEQKVSYN-CSVCNKSFNSYQALGGHKA-SH-----RKLSGGN-DDQSTSTTTS
Gh_D02G2295          --NMVQETDTFRFE-CSSCKKVFGSHQALGGHRA-SH-----KNVKGCFAITRTDGGY--
Gh_A03G1856          --NMVQETDTFRFE-CSSCKKVFGSHQALGGHRA-SH-----KNVKGCFAITRTDGGY--
Gh_A01G1467          EAFSQHLGANIRFE-CSSCKKGLASHQALGGQWA-SH-----KNVKGCFAITRSEG-Y--
Gh_D01G1704          EAFSQHLGANIRFE-CSSCKKVFASHQALGGHWA-SH-----KNVKGCFAITRSEG-Y--
Gh_A05G3205          SEASDDSLRKNKYR-CRICNKTFKSHQALGGHQT-IH-----RKSNTYAEPVEDQDKT-T
Gh_D04G0401          SEVFDDSLRKNKYR-CRICNKTFKSHQALGGHQT-IH-----RKSNTYAEPVEDHEKT-T
Gh_D08G0352          MDH---NKPKGLFE-CKACKKVFNSHQALGGHRA-SH-----KKVKGCFAARLDHFDK--
Gh_A08G0261          MDH---NKPKGLFE-CKACKKVFNSHQALGGHRA-SH-----KKVKGCFAARLDHFDK--
Gh_D12G0940          MDNKAKGVAKGLFE-CKACKKVFNSHQALGGHRA-SH-----KKVKGCFAR-LDHID---
Gh_A12G0860          MDNKAKGVARGLFE-CKACKKVFNSHQALGGHRA-SH-----KKVKGCFAR-LDHID---
Gh_A11G0712          PR-VPTVKAKGLFE-CKACKKVFNSHQALGGHRA-SH-----KKVKGCFAA---RVD---
Gh_D11G0829          PR-VPTDKAKGLFE-CKACKKVFNSHQALGGHRA-SH-----KKVKGCFAA---RVD---
```

```
Gh_A07G1370         MG-KAKGVAKGLFE-CKACKKVFNSHQALGGHRA-SH-----KKVKGCFAA---RVD---
Gh_D07G1473         MD-KAKGVAKGLFE-CKACKKVFNSHQALGGHRA-SH-----KKVKGCFAA---RVD---
Gh_D13G2392         ----NTIHQRGVFA-CKTCDKRFSSFQALGGHRA-SH-----KKPRFTDA---DL-----
Gh_A13G1993         ----NTIHQRGVFA-CKTCDKRFSSFQALGGHRA-SH-----KKPRFTDV---DL-----
Gh_A03G0330         --------AGRVFA-CKTCNKKFSSFQALGGHRA-SH-----KKPKLTVGDNEGL-----
Gh_D03G1247         --------AGRVFA-CKTCDKKFSSFQALGGHRA-SH-----KKPKLTVGDNEEL-----
Gh_A09G1486         ----KEHSVSEVFE-CKTCHRCFPSFQALGGHRA-SH-----KRPKLMGDKSNEKPQF--
Gh_D09G1496         ----KEHSVSEVFE-CKTCHRCFPSFQALGGHRA-SH-----KRPKLMGDKPNETTQF--
Gh_D09G1495         ----KEHSVSEVFE-CKTCHRCFPSFQALGGHRA-SH-----KRPKLMGDKPNETTQF--
Gh_A09G1485         ----KEHSVNEVFE-CKTCHRCFPSFQALGGHRA-SH-----KRPKLMGDKPNETIQF--
Gh_D06G2374         ----DSNLTCRVFK-CKTCNRQFTSFQALGGHRA-SH-----KKPKLMERDGGVL--E--
Gh_Sca005232G01     ----DTNLTCRVFK-CKTCNRQFTSFQALGGHRA-SH-----KKPKLMERDGGVL--E--
Gh_A13G0196         -----DSNPSRVFE-CKTCNRQFNSFQALGGHRA-SH-----KKPRLMEVDSEMSE-N--
Gh_D13G0210         -----DTNPSRVFE-CKTCNRQFNSFQALGGHRA-SH-----KKPRLVEVDSEMSE-N--
Gh_D10G2214         --DTNNNNPTRVFE-CKTCNRQFASFQALGGHRA-SH-----KKPKLLDGGST----E--
Gh_A10G2356         --DSNNNNPTRVFE-CKTCNRQFASFQALGGHRA-SH-----KKPKLLDGSST----E--
Gh_A10G1921         --DNNNNNPTRVFE-CKTCNRQFASFQALGGHRA-SH-----KKPKLLDSGST----E--
Gh_D10G2215         --DNNNNNPTRVFE-CKTCNRQFASFQALGGHRA-SH-----KKPKLLDSGST----E--
Gh_A10G1798         ASNGDGKAGYYVYE-CKTCNRTFPSFQALGGHRA-SH-----KKPKAAATVDEKIRQFTA
Gh_D10G2536         ASNGNGKAGYYVYE-CKTCNRTFPSFQALGGHRA-SH-----KKPKAAATVDEKIRQFTA
Gh_D04G0049         PSNGTGKVGYYVYE-CKTCNRTFPSFQALGGHRA-SH-----KKPKATLSEVAMVEA---
Gh_A05G3556         ASNGTGKAGYYVYE-CKTCNRTFSSFQALGGHRA-SH-----KKPKATLSEAAMVEA---
Gh_A09G2473         -ATKTNKGELYVHE-CKTCNRSFPSFQALGGHMA-SH-----KKPKGAIAEQKRPLVL--
Gh_D09G0857         -ATKTNKGELYVHE-CKTCNRSFPSFQALGGHMA-SH-----KKPKGAIAEQKRPLVL--
Gh_Sca077169G01     -ATKTNKGELYVHE-CKTCNRSFPSFQALGGHMA-SH-----KKPKGAIAEQKRPLVL--
Gh_A07G0952         IATTTNKAGFFVYE-CKTCNRSFPSFQALGGHRA-SH-----KKPKATMAEDKKPLVL--
Gh_D07G1031         IATTTNKAGFFVYE-CKTCNRSFPSFQALGGHRA-SH-----KKPKATMAEDKKPLVL--
Gh_D05G0856         MVTATNKAEVYAYE-CKTCNRSFPSFQALGGHRA-SH-----KKPKGATADEKKPLFL--
Gh_A05G0721         MVTATNKAEVYAYE-CKTCNRSFPSFQALGGHRA-SH-----KKPKGATAYEKKPLVL--
Gh_D01G2057         EPYTAAAAETDVHQ-CKTCNRCFPSFQALGGHRA-SH-----KKFKVVNDQDINNNRN--
Gh_A01G1819         EPSTAAAVETDVHQ-CKTCNRCFPSFQALGGHRA-SH-----KKFKVVNDQDINNNHN--
Gh_D04G1048         ----GKTARMNVHQ-CKTCNRCFPSFQALGGHRA-SH-----KKPKLHS-EENTKRLK--
Gh_A04G0591         ----GKTAGMNVHQ-CKTCNRCFPSFQALGGHRA-SH-----KKPKLHS-EENTKWLK--
Gh_A09G1356         -TTSKTMTGTYVHQ-CKTCNRCFPSFQALGGHRA-SH-----KKPKVANIEENNKGLM--
Gh_D09G1358         -TTSKTMTGTYVHQ-CKTCNRCFPSFQALGGHRA-SH-----KKPKVANIEENNKGLM--


Gh_A05G0618         ---------------------VDPC-----------------------------HARRS-
Gh_D05G0749         ---------------------VDPC-----------------------------HARRS-
Gh_A05G0596         --------------------KSRSC-----------------------------RGRRSV
Gh_D05G0727         --------------------KSKSC-----------------------------HGRRTV
Gh_D07G0422         EN-----------------------------------------------------GGDAT
Gh_A07G0360         EN-----------------------------------------------------GGDTT
Gh_D07G0423         EN-----------------------------------------------------GGDAT
Gh_A07G0361         EN-----------------------------------------------------GGDAT
Gh_D12G2156         --------------------------------------------------------A--T
Gh_A12G1978         --------------------------------------------------------A--T
Gh_D08G1830         ------------------------------------------------------------
Gh_A08G1529         ------------------------------------------------------------
Gh_A08G1229         EN-----------------------------------------------------VG-TT
Gh_D08G1514         EN-----------------------------------------------------VG-TT
Gh_D12G0939         EN-----------------------------------------------------VG-T-
Gh_A12G0859         EN-----------------------------------------------------VG-T-
Gh_A07G1368         EN-----------------------------------------------------VG-TG
Gh_D07G2458         EN-----------------------------------------------------VG-TR
Gh_D03G0899         ETSPEPNPTSAT------D---------------------------------TANLVSDS
Gh_A03G0614         ETSPEPNPTSAT------D---------------------------------TANFVSDS
Gh_D12G1613         ANDSFTAPMTDT------KVTKASSNGKRLGVPR-----------------G-SSYNAEK
Gh_A12G1487         ANDSFTAPMTDT------KVTKASSNGKRLGVPR-----------------G-SSYNAEK
Gh_A05G2644         ANDGFIVPTTDN------KVTKSSH-GKTLNTHR-----------------GSSSGNAEK
Gh_D05G2946         ANDGFIVPTTDK------KATKSSH-GKTLNTHR-----------------GSSSGNAEK
Gh_D13G0838         ANGSFHAPMAAT------K---------------------------------LSNGSAEK
Gh_A13G0716         ANGSFHAPMAAT------K---------------------------------LSNGNAEK
```

```
Gh_D12G0176         ---------------------------------------------------------TNA
Gh_A12G0173         ---------------------------------------------------------TNA
Gh_D13G0451         NP----------------------------------------------------STTSTA
Gh_A13G2112         NP----------------------------------------------------STTSTA
Gh_A03G1611         TT----------------------------------------------------NSTTAS
Gh_D02G2025         TT----------------------------------------------------SSTTAS
Gh_D11G2018         KTTADVSAVAAT------------------T--------------------VTPPTNLPL
Gh_A11G1957         KTTADVSAVAAT------------------T--------------------VTPPTNLPL
Gh_D12G0712         KFT---------------------------A--------------------STTATNSHP
Gh_A12G0699         KFT---------------------------A--------------------STTATNSHA
Gh_A02G0836         ST----------------------------------------------------------
Gh_A01G0984         AV----------------------------------------------------------
Gh_D01G1033         AV----------------------------------------------------------
Gh_D06G2303         GT----------------------------------------------------AGGVVK
Gh_D05G2011         AT----------------------------------------------------AGGVIS
Gh_A05G1815         AT----------------------------------------------------AGGVIS
Gh_D02G2295         -------EV---------------------------------------------------
Gh_A03G1856         -------GV---------------------------------------------------
Gh_A01G1467         -------DVKDH------SFD---------------------------------------
Gh_D01G1704         -------DVKDH------SCD---------------------------------------
Gh_A05G3205         ---HISSSPEIE----------AGCR--LVKV------EYVENSV-------DQEMNEVT
Gh_D04G0401         ---HISSSPEIE----------AGCK--LVKV------EYVEHSV-------DQEMNEVT
Gh_D08G0352         ----TQQVDEDHDVITHEELLPTKSTS-HIQLDQG------------------TSTPFAA
Gh_A08G0261         ----TQQVDEDHDVITHEELLPTKSTS-HIQLDQG------------------TSTPFAA
Gh_D12G0940         ----DSQVDEDLDVITHEEFFPTKSRSTTLQFDPG------------------TSTNLLA
Gh_A12G0860         ----DSQVDEDLDVITHEEFFPTKSRSTTLQFDPG------------------TSTNPLA
Gh_A11G0712         ----DSLADVDH-----HGFFLKKPTS-TFRFD-----------------------NTLA
Gh_D11G0829         ----DSLADEDH-----HGFFLKKPTS-TFRFD-----------------------DTLA
Gh_A07G1370         ----HKQADEDHDVITHEEFFPTKSMS-TLQFHQG------------------TN-TPLA
Gh_D07G1473         ----HNQADEDHDVITHEEFFPTKSMS-TLQFHQG------------------TS-TPLA
Gh_D13G2392         ------------------------------------------------------------
Gh_A13G1993         ------------------------------------------------------------
Gh_A03G0330         ------------------------------------------------------------
Gh_D03G1247         ------------------------------------------------------------
Gh_A09G1486         ------------------------------------------------------------
Gh_D09G1496         ------------------------------------------------------------
Gh_D09G1495         ------------------------------------------------------------
Gh_A09G1485         ------------------------------------------------------------
Gh_D06G2374         ------------------------------------------------------------
Gh_Sca005232G01     ------------------------------------------------------------
Gh_A13G0196         ------------------------------------------------------------
Gh_D13G0210         ------------------------------------------------------------
Gh_D10G2214         ------------------------------------------------------------
Gh_A10G2356         ------------------------------------------------------------
Gh_A10G1921         ------------------------------------------------------------
Gh_D10G2215         ------------------------------------------------------------
Gh_A10G1798         GTTTIGLSDEEEGQHQQQQFMKITNNVSSLSLQLSNTNNYNNHNINNRVLYGNNNKDNNN
Gh_D10G2536         GTTTIGLSDEEEGQHQQQQFMKT-NNVSSLSLQLSNTNNYNNHNINNRVLYGNSNKDNNN
Gh_D04G0049         -------AYEE-----------------------------------------QGPNYNNN
Gh_A05G3556         -------AYEE-----------------------------------------QGPNNN-N
Gh_A09G2473         -------AVNDAV----------------IALRV--------------------------
Gh_D09G0857         -------AVNDAV----------------IALRV--------------------------
Gh_Sca077169G01     -------AVNDAV----------------IALRV--------------------------
Gh_A07G0952         -------AVKDDKHVDQEL--QSET--PLLALQVS-----N--------------NNSKG
Gh_D07G1031         -------AVKDDKHVDQEP--QSET--PLLALQVT-----N--------------NNSKG
Gh_D05G0856         -------AFKDHGVEDADQFNRESP--AVLALQVG--NN----------------NNKTN
Gh_A05G0721         -------AFKDHGVEDADQFNRESP--AVLALQVG--NNYN--------------NNKTN
Gh_D01G2057         -------KEDHR---HYDQFNEKAT---TLSLHIT--S-----------------K----
Gh_A01G1819         -------KEDHR---HYDQFNEMAT---TLSLHIT--S-----------------K----
Gh_D04G1048         -------FVKED--------DDMNT---TLSLQIT--N-----------------KAPAL
Gh_A04G0591         -------FVKED--------DDRNT---TLSLQIT--S-----------------KAPAL
Gh_A09G1356         -------FMKED---VQQPFNNMNT---TLSLQIT--N-----------------K-PTL
Gh_D09G1358         -------FMKED---VQQPFSDMNT---TLSLQIT--N-----------------K-PAL
```

```
Gh_A05G0618          RKNKAVKSEHQCEICGKTFETGQALGGHKTYHRVKKNKVELLQQGKTK----QEPCMMTP
Gh_D05G0749          RKNKAVKSEHQCEICGKTFETGQALGGHKTYHRVKKNKVELLQQGKTK----QEPCMMTP
Gh_A05G0596          KQTKAVKSVHRCEICDKTYETGQALGGHKSYHRVKDPLKQRKTEQQSC----GEVKMVTG
Gh_D05G0727          KKTKAVKSVHRCEICDKTYETGQALGGHKSYHRVKDPLKQRKTEQQSC----GEVKMVTR
Gh_D07G0422          DHKIHQQRTYL-------------------------------------------------
Gh_A07G0360          DHKIHQQRTYL-------------------------------------------------
Gh_D07G0423          DHKIHQQRIFECEFCDRVFQSGQALGGHKKIHFGYSPSVTH---NRI-------------
Gh_A07G0361          GHKIHQQRIFECEFCDRVFQSGQALGGHKKIHFGYSPSVTH---NRI-------------
Gh_D12G2156          TAAGNNSKIFECPLCYRVFGSGQALGGHKRSHLLAATPN---------------------
Gh_A12G1978          TAAGNNSKIFECPLCYRVFGSGQALGGHKRSHLLAATPN---------------------
Gh_D08G1830          ----ADSKRFECPFCYRVFGSGQALGGHKRSHLLASASSN----TA--------------
Gh_A08G1529          ----ADSKRFECPFCYRVFGSGQALGGHKRSHLLASASSN----TA--------------
Gh_A08G1229          -CSMTEKKTHKCPVCFKVFSSGQALGGHKRSHVAAA-----TTQTYV-------------
Gh_D08G1514          -CSMTEKKTHECPVCFKVFSSGQALGGHKRSHVAAA-----TAQTYV-------------
Gh_D12G0939          --SIPEKKTHECPVCFRIFSSGQALGGHKRSHVTGQVRATSTTKTAV-------------
Gh_A12G0859          --SIPEKKTHECPVCFRIFSSGQALGGHKRSHVTGQVIATSTTKTAV-------------
Gh_A07G1368          PSSMTDKKIHECPVCFRVFSSGQALGGHKRSHVVA------TTETPV-------------
Gh_D07G2458          PSSMTDKKIHECPVCFRVFSSGQALGGHKRSHVVA------TTETPV-------------
Gh_D03G0899          YGVVKKNKGHECPICLKVFPSGQALGGHKRSHLVAEAKENRSQTIET-------------
Gh_A03G0614          NGVVKKNKGHECPICLKVFPSGQALGGHKRSHLVAEAKENRSQTIET-------------
Gh_D12G1613          RLGSKKNKGHECPFCFRVFKSGQALGGHKRSHFVGGSDD-RTLVIKQ-------------
Gh_A12G1487          RLGSEKNKGHECPFCFRVFKSGQALGGHKRSHFVGGSDD-RTLAIKQ-------------
Gh_A05G2644          RLGSKKNKGHQCPFCFRVFKSGQALGGHKRSHFVGGSED-RTLVIKQ-------------
Gh_D05G2946          RLGSKKNKGHQCPFCFRVFKSGQALGGHKRSHFVGGSED-RTLVIKQ-------------
Gh_D13G0838          RLGSKKNKGHVCPFCFRVFKSGQALGGHKRSHFAGGSED-TTLVIKR-------------
Gh_A13G0716          RLGSKKNKGHVCPFCFRVFKSGQALGGHKRSHFAGGSEE-TTLVIKQ-------------
Gh_D12G0176          AGSSGRGRSHECSICHKSFPTGQALGGHKRCHYEGGNNNSSS----------YKSGSVSG
Gh_A12G0173          AGSSGR----------------------------GGNNNSSS----------YKSGSVSG
Gh_D13G0451          TTATSSGRLHECSICHKSFPTGQALGGHKRCHYEGGNNNNNN-----KNNNNSGSVSVSG
Gh_A13G2112          TTITSSGRLHECSICHKSFPTGQALGGHKRCHYEGGNNNNKN-----NNNSGSVSVSVSG
Gh_A03G1611          GETGGNGKAHKCSICHKSFPTGQALGGHKRCHYEGGNTTTTT-----NNNNKSSSVSLSG
Gh_D02G2025          GEAGGNGKAHKCSICHKSFPTGQALGGHKRCHYEGGNTTTTTTTTAAITNNKSSSVSLSG
Gh_D11G2018          MINRVGNKNHVCSICNKTFSSGQALGGHKRCHYEAGGNNN-N----------G-------
Gh_A11G1957          MINRVGNKNHVCSICNKTFSSGQALGGHKRCHYEAGSNNN-S----------G-------
Gh_D12G0712          MISNQGGKTHTCSICYKTFSSGQALGGHKRCHYEAGSNNNNS----------G-------
Gh_A12G0699          MISNQGGKTHTCSICYKTFSSGQALGGHKRCHYEAGSNNNNS----------G-------
Gh_A02G0836          STSNPSGRSHQCSICHKSFPSGQALGGHKRLHYEGCAGTARG----------V-------
Gh_A01G0984          -GSNPSGRSHECSICHKSFPSGQALGGHKRCHYEGGAANTG-------------------
Gh_D01G1033          -GSNPSGRSHECSICHKSFPTGQALGGHKRRHYEGGAANTG-------------------
Gh_D06G2303          ---SSGGKSHECSICHKSFPTGQALGGHKRRHYEGGAGNNNA----------GATATASS
Gh_D05G2011          SALNPSGKTHQCSICHKSFPTGQALGGHKRCHYEGGAGNSA-----------SVTASASG
Gh_A05G1815          SALNPSGKTHQCSICHKSFPTGQALGGHKRCHYEGGAGNNA-----------SVTASASG
Gh_D02G2295          DDKTVMFLGHKCSICLRVFSSGQALGGHKRCHWDKNDEP--SLNH---------------
Gh_A03G1856          DDNTVMFLGHKCSICLRVFSSGQALGGHKRCHWDKNDEP--SLNH---------------
Gh_A01G1467          EDKMMMVLGHKCSICWKLLSSGQALGGHKRYRWEKRDDEASSLNL---------------
Gh_D01G1704          EDKMMMVLGHKCSICWKLLSSGQALGGHKRYHWGKGDDEASSLNL---------------
Gh_A05G3205          SSETRVYKVHKCLICLKVFGSGQALGGHKRSHISRDPGPGDKQPAKQL------------
Gh_D04G0401          SSETRVYKVHKCLICLKVFGSGQALGGHKRSHISRDPGPGDKQPAKQL------------
Gh_D08G0352          STSKKKLKVHECSICHRVFSSGQALGGHKRCHWVTSNSPD-TSSLTKF------------
Gh_A08G0261          STSKKKPKVHECSICHRVFSSGQALGGHKRCHWVTSNSPD-TSSLTKF------------
Gh_D12G0940          STSKRKSKVHECSICHRVFSSGQALGGHKRCHWITSNSPD-ISSLVKF------------
Gh_A12G0860          STSKRKSKVHECSICHRVFSSGQALGGHKRCHWITSNSPD-ASSLVKF------------
Gh_A11G0712          STSKKKSKVHECSICHRVFSSGQALGGHKRCHWITSNSPVETSSLVKF------------
Gh_D11G0829          STSKKKSKVHECSICHRVFSSGQALGGHKRCHWITSNSPVETSSLGKF------------
Gh_A07G1370          SPSKKKSKVHECSICYKVFSSGQALGGHKRCHWITSNAPD-ASSLAKF------------
Gh_D07G1473          SPSKKKSKVHECSICHRVFSSGQALGGHKRCHWITSNAPD-TSSLAKF------------
Gh_D13G2392          PASPKKPKTHECSICGLEFPIGQALGGHMRKHRVVSNEGMVVTRPVTE------------
Gh_A13G1993          PVSPKKPKTHECSICGLEFPIGQALGGHMRKHRVVSNEGMVVTRPVTE------------
Gh_A03G0330          AVSPKKPKTHECSICGLEFAIGQALGGHMRRHRAALNDGLVTRDLLPE------------
Gh_D03G1247          AVSPTKPKTHECSICGLEFAIGQALGGHMRRHRAALNDGLVTRDLLPE------------
Gh_A09G1486          LCLSTKPRTHECSICGQEFSTGQALGGHMRRHRATMNESFSPFALV--------------
```

```
Gh_D09G1496          LCLSTKPRTHECSICGQEFSTGQALGGHMRRHRAAMNETFSPFPLV--------------
Gh_D09G1495          LSLSTKPKTHECSICGQEFSMGQALGGHRRRHRAAMNESFSPFPLV--------------
Gh_A09G1485          PSLSTKPKTHECSICGQEFSMGQALGGHMRRHRVAMNESFSPFPLV--------------
Gh_D06G2374          NQPPAKPKMHECSICGLEFSIGQALGGHMRRHRANLSEENHQEP----------------
Gh_Sca005232G01      NQPPAKPKMHECSICGLEFSIGQALGGHMRRHRANLSEENHQEP----------------
Gh_A13G0196          QASPAKPKTHECSICGLEFAIGQALGGHMRRHRGVLSENQHE------------------
Gh_D13G0210          QASPAKPKTHECSICGLEFAIGQALGGHMRRHRGVLSENQHE------------------
Gh_D10G2214          NQPPAKPKTHECSICGLEFSIGQALGGHMRRHRGGLSGNHHQYGPLSS------------
Gh_A10G2356          NQPPAKPKTHECSICGLEFSIGQALGGHMRRHRGGVSENHHQYGPLSS------------
Gh_A10G1921          NQPQAKPKTHECSICGLEFSIGQALGGHMRRHRGGSSENQHQHGPLSS------------
Gh_D10G2215          NQPPAKPKTHECSICGLEFSIGQALGGHMRRHRGGSSENQHQHGPLSS------------
Gh_A10G1798          NNNGKPNKVHECSICGSEFTSGQALGGHMRRHRGSIGGGSNSVVAANT------------
Gh_D10G2536          NNNGKPNKVHECSICGSEFTSGQALGGHMRRHRGSIGGGSNSAVAANT------------
Gh_D04G0049          NNSKVKVKVHECSICGAEFTSGQALGGHMRRHRGSIGNQVNV-LTTNT------------
Gh_A05G3556          NNSKVKVKVHECSICGAEFTSGQALGGHMRRHRGSIGNHVNV-LTTNT------------
Gh_A09G2473          -SNKKGNKIHECSICGSEFTSGQALGGHMRRHRTASAAAASNQ-----------------
Gh_D09G0857          -SNKKGNKIHECSICGSEFTSGQALGGHMRRHRTASAAAASNQ-----------------
Gh_Sca077169G01      -SNKKGNKIHECSICGSEFTSGQALGGHMRRHRTASAAAASNQ-----------------
Gh_A07G0952          CQVNKGNKIHECSICGSEFSSGQALGGHMRRHRAAAATTAS-----N-------------
Gh_D07G1031          CQVNKGNKIHECSICGSEFSSGQALGGHMRRHRAAAATTAS-----N-------------
Gh_D05G0856          CHGNKGNKIHECSICGSEFLSGQALGGHMRRHRAAASNQAAVSVDTT-------------
Gh_A05G0721          CHGNKGNKIHECSICGSEFLSGQALGGHMRRHRAAASNQAAVSVDTT-------------
Gh_D01G2057          -----KSRVHECSICGAEFSSGQALGGHMRRHRTLTNAPTMTTPATAT------------
Gh_A01G1819          -----KPRVHECSICGAGFSSGQALGGHMRRHRTLTNAPTTKTPATAT------------
Gh_D04G1048          CNG-TKSKVHECSICGAEFSSGQALGGHMRRHRTSTNATTTVGTSTSA------------
Gh_A04G0591          CNG-TKSKVHECSICGAEFSSGQALGGHMRRHRTSTNATTTVGTSTSG------------
Gh_A09G1356          CHI-SKPKVHECSLCGAEFSSGQALGGHMRRHRTIPNAAAAAATTIVR------------
Gh_D09G1358          CHI-SKPKVHECSMCGAEFSSGQALGGHMRRHRTIPNAA--AATTIVR------------


Gh_A05G0618          MLL------PHLSQQP---DLSHPKTMLDFDLNIPYQQ----------------------
Gh_D05G0749          MLL------PHLSQQP---DLSHPKTMLDFDLNIPYQQ----------------------
Gh_A05G0596          MVL------PGLAPEED--DKNPTKRMLDFDFNIPYKE----------------------
Gh_D05G0727          MVL------PGLPPEEA--DKNPSKRMLDFDFNISYEE----------------------
Gh_D07G0422          ------------------------------------------------------------
Gh_A07G0360          ------------------------------------------------------------
Gh_D07G0423          -------------------SIKFKPTVQPFDLNLPPSEQDDDEVSLAQNTVPQP-----S
Gh_A07G0361          -------------------SIKFKPTVQPFDLNLPPSEQDDDEVSLAQNTVRRP-----S
Gh_D12G2156          ---------------------SGKFYNNLIDLNLPAPLEDDEFS-----VVSDA------
Gh_A12G1978          ---------------------SGKFDNNLIDLNLPAPLEDDDFS-----VVSDA------
Gh_D08G1830          -------------------AKFTKFDNNFIDLNLPAPLEDDEFS-----VVSDA------
Gh_A08G1529          -------------------AKFTKFDNNFIDLNLPAPLEDDEFS-----VVSDA------
Gh_A08G1229          -------------------KSSNKLVVNFIDLNLPAPVDDDD-DDDGFLMLK--------
Gh_D08G1514          -------------------KSSNKLVVNFIDLNLPAPVDDDDDDDDGFLMLK--------
Gh_D12G0939          -------------------TSSKKLGDSLIDLNLPAPMDDDDISQIELSAVSDA-----E
Gh_A12G0859          -------------------TSSKKLGDSLIDLNLPAPMDDDDISQIELSAVSDA-----E
Gh_A07G1368          -------------------RSSKKLGDSFIDLNLPAPMDDDDASQIELSAVSDA-----E
Gh_D07G2458          -------------------RSSKKLGDSFIDLNLPAPMDDDDASQIELSAVSDA-----E
Gh_D03G0899          -------------------QNTIPEIRDFLDLNLPAPVEEGTSS-VHMGFEPWW------
Gh_A03G0614          -------------------QNPIPEIRDFLDLNLPAPVEEGTSS-VHMGFEPWW------
Gh_D12G1613          -------------------DSSDMP--ALIDLNLPAPVEEDAFG-NAAGFMP--------
Gh_A12G1487          -------------------DSSDMP--ALIDLNLPAPVEEDALG-NATGFMP--------
Gh_A05G2644          -------------------NSPEMPTATIIDLNLPAPVEDDAMG-N-VGFMPWE------
Gh_D05G2946          -------------------NSPEMPTATIIDLNLPAPVEDDGMG-N-VGFMPWE------
Gh_D13G0838          -------------------DSPDMPLPALIDLNLPAPLEEDAME-N-SGFIPW-------
Gh_A13G0716          -------------------DSPDMPLPALIDLNLPASLEEDAME-N-SGFIPW-------
Gh_D12G0176          VTLSDGG-------ALRQ---SH-RLNFDFDLNMPACVEN---E----DGR-FG------
Gh_A12G0173          VTLSDGG-------ALSQ---SH-RLNFDFDLNMPACEEN---E----DGR-FG------
Gh_D13G0451          VTSSDGG-------ALSH---NHRAVDFDFDLNLPALPEF-------------S------
Gh_A13G2112          VTSSDGG-------ALSH---NHRAVDFDFDLNLPALPEF-------------S------
Gh_A03G1611          VTVSDGG-------ALSQ----TRCVDFDFDLNLPALCELENKD----GSR-FS------
Gh_D02G2025          VTVSDGG-------ALSQ----TRCVDFDFDLNLPALCELEIKD----GSR-FC------
Gh_D11G2018          ---SDGV-------K------LSRQSQRDFDLNLPAEPQELSIP----DVNQRD------
```

```
Gh_A11G1957          ---SDGA-------K------LPSQSQRDFDLNLPAGPQELSTP----DVNQRD------
Gh_D12G0712          ---NDGV-------K------SWSQSQRDFDLNLPAGPDETSMD----V-LRQD------
Gh_A12G0699          ---NDGV-------K------SWSQSQRDFDLNLPAGPDETSMD----V-LRQD------
Gh_A02G0836          -TTPERS-------AS-----TSSTSQRGLDLNLPAFPELSPSK----LFVS--------
Gh_A01G0984          SITSEGV-------SSTSTN----TTHRDFDLNIPGLPAFSPAN----LFVS--------
Gh_D01G1033          SITSEGV-------SSTSTN----TTHRDFDLNIPALPAFSPAN----LFVS--------
Gh_D06G2303          VTESEGV-------GSTNTNTNTHISNRDFDLNMPVLLEFSPAN----FFVS--------
Gh_D05G2011          VTTSEGV-------GSTNTMSHI-SQRIDFDLNLPALPEFSPAN----FFVS--------
Gh_A05G1815          VTTSEGV-------GSTNTMSHI-SQRIDFDLNLPALPEFSPDN----FFVS--------
Gh_D02G2295          -------------------FEPGQGGC--LDLNLPAPTPPVELENASSSSYSSCMALDLS
Gh_A03G1856          -------------------FEPRQVGC--LDLNLPAPTPPVELENGSSSSYSSCMALDLS
Gh_A01G1467          -------------------LTAKEDCG--LDLNLPP----AALQ---NDSYPSGLALDLR
Gh_D01G1704          -------------------LTAKEDCG--LDLNLPP----APLQ---NDSYPSGLALDLR
Gh_A05G3205          -----------------DLSNISD----VIDLNLPVMHNEEANGDTRLKLCRL-------
Gh_D04G0401          -----------------DLSNISD----VIDLNLPVMHNEEANGDTRLKLCRL-------
Gh_D08G0352          HRFQDQT-AHIRQKP--K-LNIDQSLDLKLDLNLPATTD-LDLV-RRNHVNP--------
Gh_A08G0261          HRFQDQT-AHIRQKP--K-LNIDQSLDLKLDLNLPATTD-LDLV-RRNHVNP--------
Gh_D12G0940          HQVEDHI-EQIQ-QQRPKFVDKPEPLDLNLDLNLPAPT--DDNV-RRN----------RE
Gh_A12G0860          HHFEDHI-EQIQ-QQRPKFIGKPEPLDLKLDLNLPAPT--DDNV-RRN----------RE
Gh_A11G0712          HQLQHQV-QQIQQTQRPNFVDDSEPLDLNLPA----P--VDKLV-RRNHIHPSSFDVSTK
Gh_D11G0829          HQLQHQV-EQIQQTQRPNFVDDSEPLDLNLPA----P--VDELV-RRNHIHPSSFDVSTK
Gh_A07G1370          HRFQDQM-EMN--QQTPKFIDNSEPLNLKLDLNLPAAGDHHDAL-RRKHVNPSSFKVSTG
Gh_D07G1473          HRFQDQM-ELN--QERPKFIDNSEPLNLKLDLNLPAAGGHHDAL-RRKHVNPSSFKVSTG
Gh_D13G2392          -K------------LNGDDADNDGGLC--LDLKLAPCGIDLDLK-----LEKAA------
Gh_A13G1993          -K------------LNGDDADNDGGLC--LDLKLAPCGIDLDLK-----LEKAA------
Gh_A03G0330          -M------------NKSTG--DGRDPS--IDLGLTSWGVDLELK-----LGKVT------
Gh_D03G1247          -M------------NKSTG--DGRDPS--LDLSLTSWGVDLELK-----LGKVT------
Gh_A09G1486          ----------PTVPVLKRSNSSRRVVC--LDLNLTPLENDLQVL-----FGNKA------
Gh_D09G1496          ----------PTVPVLKRSNSSRRVVC--LDLNLTPLENDLQVL-----FGNKA------
Gh_D09G1495          ----------PTVPVLKRSNSSRRVVC--LDLNLTPLENDLQVL-----FGNKA------
Gh_A09G1485          ----------PTVPVLKRSNSSRRVVC--LDLNLTPLENDLQVL-----FGNKA------
Gh_D06G2374          ----------LMSPLVKK-A--NKVWC--LDLNLTPFENDLELL-----KLAEP------
Gh_Sca005232G01      ----------LMSPIVKK-S--NKVWC--LDLNLTPFENDLELL-----KLAKP------
Gh_A13G0196          ------------EPIVKK-SNSRRVLC--LDLNLTPLENDLELF-----KLGKG------
Gh_D13G0210          ------------EPIVKK-SNSRRVLC--LDLNLTPLENDLELF-----KLGKG------
Gh_D10G2214          -SSSSQE--MVTTPIVKK-SNSRRVLC--LDLNLTPLENDWELF-----KLGKA------
Gh_A10G2356          -SSSSQE--MVTTPIVKK-SNSRRVLC--LDLNLTPLENDWELF-----KLGKA------
Gh_A10G1921          -STSSQE--TVRTPIVKK-PNSRRVLC--LDLNLTPLENDLELF-----KLGKA------
Gh_D10G2215          -STSSQE--TVRTPIVKK-PNSRRVLC--LDLNLTPLENDLELI-----KLGKA------
Gh_A10G1798          -ALSLKVATPLEQPQQP--KKPKNVLSLDLDLNLPAPDDVDHRE-PKFSFASKQ------
Gh_D10G2536          -ALSLKVATPLEQPQQP--KKPKNVLSLDLDLNLPASDDVDHRE-PKFSFASKQ------
Gh_D04G0049          -TLSLTVPMALE-SEKP--KKPPNVLSLSLDLNLPAPED-DHRE-SEFTFASNQ------
Gh_A05G3556          -TLSLTVPMALE-SEKP--KKPPNVLSLSLDLNLPAPED-DHRE-SEFTFASSQ------
Gh_A09G2473          ------D---AMNGNDD--IKPRNITP--LDLNLPAPEDDVRD--SMFEFG---------
Gh_D09G0857          ------D---AMNGDDD--IKPRNITP--LDLNLPAPEDDVRD--SMFEFG---------
Gh_Sca077169G01      ------D---AMNGDDD--IKPRNITP--LDLNLPAPEDDVRD--SMFEFG---------
Gh_A07G0952          ---QVVV---SVGNGDG--IKPRNILS--LDLNLPAPEDDLRD--SKFQFG---------
Gh_D07G1031          ---QVVV---SVGNGDG--IKPRNILS--LDLNLPAPEDDLRD--SKFQFG---------
Gh_D05G0856          ---SIPI---ESGNGDG--TKPRNILA--LDLNLPAPEDDLRD--AKFQFG---------
Gh_A05G0721          ---SIPI---ESGNGDG--NKPRNILA--LDLNLPAPEDDLRD--AKFQFG---------
Gh_D01G2057          -TTLDTV---VRTSEQR--KKPRTVLQ--LDLNLPAPEDDLHKE-PNNKVLSLA------
Gh_A01G1819          -TTLDTV---VRTSEQR--KKPRTVLQ--LDLNLPAPEDDLHKE-PNNKVLSFA------
Gh_D04G1048          ----------ETEDLSS--NKPTTVLQ--LDLNLPAPEDDRHNE-SNFPFT--Q------
Gh_A04G0591          ----------ETEDLSS--NKPTTVLQ--LDLNLPAPEDDRHNE-SNFPFT--E------
Gh_A09G1356          ----------PESDDKP--KKPRTVLQ--LDLNLPAPEDDHQRE-TKFSFA--S------
Gh_D09G1358          ----------PESDDKP--KKPRTVLQ--LDLNLPAPEDDHQRE-TKFSFA--S------
```

Subgroup 3

```
Gh_D05G0878      --HLGCFVFTAAKDNHKATKKRKSSPLSPNSHKH----RKALSSTWT-F-----------
Gh_A05G0747      --HLGCFVFTAAKDNHKATKKRKSSPLSPNSHKH----RKALSSTWT-F-----------
Gh_A07G0936      --HLGCFIFTAK-DHQKPPKKRKHWPLSPSSPSHKPHANKSLSSTWS-Y-----------
Gh_D07G1014      --HLGCFIFTAK-DHQKPPKKRKHSPLSPSSPSHKPHANKSLSSTWS-Y-----------
```

```
Gh_D01G0570      -----------------------------------------MPTVWF-S-----------
Gh_A01G0562      -----------------------------------------MPTVWF-S-----------
Gh_A05G1425      -----------------------------------------MSSAWLTS-----------
Gh_D05G1597      -----------------------------------------MSSAWLTS-----------
Gh_A01G0902      ------------------------------------------------------------
Gh_D01G0942      IMALLTFLPEAAAEPMKQ---QPK-----RRRKQVKKQKQPTTSSWD-Q-----------
Gh_A05G1595      -MALLTFLPEPAAEPMKV---EQPSK--RGRRNPVKKQKEKQPSSWD-Q-----------
Gh_D05G1782      -MALLTFLPEPAAEPMKV---EQPSK--RGR-NPVKKQKEKQPSSWD-Q-----------
Gh_A06G0349      -MAGPTHQRKPSKDNYHN---QKKKR--RPQPQPQPQKNDKPPSNWS-L-----------
Gh_D06G0380      -MAGPTHQRKPSKDTYHN---QKKKR--RPQPQPQPQKNDKPPSNWS-L-----------
Gh_D05G2208      -MAGSTHQRKPTMEAYQN---QNKW----------PQKIDK-PPSWA-V-----------
Gh_A05G2001      -MAGSTHQRKPTMEAYQN---QNKW----------PQKIDK-PPSWA-V-----------
Gh_A01G1610      ---QNAHRLVKEPMKMSPVL----------------------------------------
Gh_D01G1850      ---QNAHRLVKEPMKMSPVL----------------------------------------
Gh_A10G1157      ---IEGFKLVPSAEQITRTN--------GSDDEPVPYDDHKNPNAAMEFQDGRFGVGRQD
Gh_D10G1339      ---IEGFKLVPSAEQITRTN--------GSDDEPVPYDDHKNPNAAMEFQDGRFGVGRQD

Gh_D05G0878      ---------------LKRIFSS-KA-----------------------------------
Gh_A05G0747      ---------------LKRIFSS-KA-----------------------------------
Gh_A07G0936      ---------------LKRIFTSSKA-----------------------------------
Gh_D07G1014      ---------------LKRIFTSSKA-----------------------------------
Gh_D01G0570      ---------------LKRSLHCKSEP-SDVHDPKTRKQLS--------------------
Gh_A01G0562      ---------------LKRSLHCKSEP-SDVHDPKTRKQLS--------------------
Gh_A05G1425      ---------------LKRSVNCKPKL-PDVKEPAANDSTE--------------------
Gh_D05G1597      ---------------LKRSVNCKPKL-PDVKEPAADDSTE--------------------
Gh_A01G0902      ------------------------MEGSKVHDPSKNNNP------APPHHH---------
Gh_D01G0942      ---------------IKNLLTCKQMEGSKVHDPSKNNNP------APPHHH---------
Gh_A05G1595      ---------------IKNLLSCKQIEGSKVHDPSKNNQL----------PH---------
Gh_D05G1782      ---------------IKNLLSCKQIEGSKVHDPSKNNQL----------PH---------
Gh_A06G0349      ---------------VKGLFFGKPQQQQQHQQQQQQ------QQQQQQQRE---------
Gh_D06G0380      ---------------VKGLFFGKPQQQQQHQQQQQQQQQ-QQQQQQQQQRE---------
Gh_D05G2208      ---------------LKGFFFGKHHHHQQEQEEQQKLQPKKKVKKQKQQQK---------
Gh_A05G2001      ---------------LKGFFFGKHHHHQQEQEEQQKLQPKKKVKKQKQQQK---------
Gh_A01G1610      ------------------------------------AAVLNN--PLPPFHKPFLIPPKTK
Gh_D01G1850      ------------------------------------AAVLNN--PLPPFHKPFLIPPKTK
Gh_A10G1157      SLDNVGKPDTVAEKDLPATISLKHLEDSEIVQPPQNSAESSQ--NENPFTKSMLFVSNGT
Gh_D10G1339      SLDNVGKPNTVAEKDLPATISLKHLEDSDIVQPPQNSAESSQ--NENPFTKSMLFVSNGT

Gh_D05G0878      -------------------NCINTIQ----------------------AAHPPTSATTPA
Gh_A05G0747      -------------------NCINTIQ----------------------AAHPPTSATTPA
Gh_A07G0936      -------------------NCKNTIP------------------------THPTAAPTPA
Gh_D07G1014      -------------------NCRNTIP------------------------THPRAAPTPA
Gh_D01G0570      ------TILTRKA---GRSGCSRSIANL-----------KDVIHGSKRHLEKPPS-CSPR
Gh_A01G0562      ------TILTRKA---GRSGCSRSIANL-----------KDVIHGSKRHLEKPPS-CSPR
Gh_A05G1425      ------K-NSPCL---CSSGCSRSLSNL-----------RDVIHGSKRQADKAPRIGSPT
Gh_D05G1597      ------K-NSPCL---CSSGCSRSLSNL-----------RDVIHGSKRQADKAPRIGSPT
Gh_A01G0902      --QHGYSKLGSSC---S------SICSF-----------RDVVHGNTRVVHRADNSPESS
Gh_D01G0942      --QHGYSKLGSSC---S------SICSF-----------RDVVHGNTRVVHRADNSPESS
Gh_A05G1595      --HHGYSKLGSSC---K------SICSF-----------KDVVHGNTRVVHRADNSPESS
Gh_D05G1782      --HHGYSKLGSSC---K------SICSF-----------NDVVHGNTRVVHRADNSPESS
Gh_A06G0349      --HVVVDETGKKC---KKMRCSGSLCSN-----------TKIM-ERPVTASPELNK-KRA
Gh_D06G0380      --HVVVDETGKKC---KKMRCSGSLCSN-----------TKIM-ERPVTASPELNK-KRA
Gh_D05G2208      --EQVMEETGKKC---KKMRCSGSLCSN-----------TKVIHCRPEMASPEVHK-KRA
Gh_A05G2001      --EQVMEETGKKC---KKMRCSGSLCSN-----------TKVIHCRPEMASPEVHK-KRA
Gh_A01G1610      -----------------------------------PAANKELISKQPTANQSPRHHPYK-
Gh_D01G1850      -----------------------------------PAANKELISKQPTANQSPRHHPYK-
Gh_A10G1157      LEHQGVGLSYSKDSEDRTASASGVVPIEAETLKGVPEESREVDPGAS-AAECSVEQPTNT
Gh_D10G1339      LEHLDVGLSYSKDSEDRTASVSGVVPTEAETLKGAPEESREVGAGAS-AAECSVEQPTNT

Gh_D05G0878      -LTFA-----R---------------------------------------------NS--
```

```
Gh_A05G0747     -LTFA-----R---------------------------------------------NS--
Gh_A07G0936     -LTSA-----R---------------------------------------------NS--
Gh_D07G1014     -LTSA-----R---------------------------------------------NS--
Gh_D01G0570     SIGSSEF-LNPI-THEVILSNSR-----------------CELKITGFG-------GFQD
Gh_A01G0562     SIGSSEF-LNPI-THEVILSNSR-----------------CELKITGFG-------GFQD
Gh_A05G1425     SIASSEI-INQI-THQAVFTDSK-----------------CVLEIKTEC-----------
Gh_D05G1597     SIASSEI-INQI-THQDVFTDSK-----------------CVLEIKTQC-----------
Gh_A01G0902     TLGQETA-LLRR---------------------------------KAVN-------GSS-
Gh_D01G0942     TIGQETA-LLRR---------------------------------KAVN-------GSS-
Gh_A05G1595     TVGQETG-LLRR---------------------------------KAAN-------SSS-
Gh_D05G1782     TVGQETG-LLRR---------------------------------KAGN-------SSS-
Gh_A06G0349     SLGSVKA-PVQ----------------------------------HEPN-------GVV-
Gh_D06G0380     SLGSVKA-PVQ----------------------------------HEPN-------GVV-
Gh_D05G2208     SFGSFDT-SSRS-M--------K-----------------AP--LRELN-------GVAV
Gh_A05G2001     SFGSFDT-SSRS-M--------K-----------------AP--LREIN-------GVAV
Gh_A01G1610     -RHDEEANATDQLQDKLMKQTTQEGEDFLRQL----------------------------
Gh_D01G1850     -RHDEEANATDQLQDKLMKQTTQEGEDFLRQL----------------------------
Gh_A10G1157     -NGNEEGELNENLLDGLILPSEQAAGEVSRTVPTSEKTLGLTLDIFQLNEDSSDRLASKT
Gh_D10G1339     -NGNEEGKLNANLLDGLILPSEQAAGEVSRTVPTSEKTLGLTLDIFQLNEDFSDRLASKT

Gh_D05G0878     ---------------------QHSLV----------------SMSMINPPETHLSDSPPP
Gh_A05G0747     ---------------------QHSLV----------------SMSMINPPETHLSDSPPP
Gh_A07G0936     ---------------------QQSLV----------------SMVIL--PETHLSETSLP
Gh_D07G1014     ---------------------QQSLV----------------SMVIP--PETHLSETSLP
Gh_D01G0570     SLTNGGNMDANGGGGGGGSGGGSTFVGTLRPGTPGPGGH--PTMHYFNPSFKNSSTTTPP
Gh_A01G0562     SLTNGGNMDANGGGGGGGSGGGSTFVGTLRPGTPGPGGH--PTMHYFNPSFRNSSTTTPP
Gh_A05G1425     -------------SGNDDGKGGSTFVGTLRPGTPGPGDR--FME----PAYNSRRSRK-I
Gh_D05G1597     -------------SANDDGKGGSTFVGTLRPGTPGPGDR--FME----PAYNSRRSRK-I
Gh_A01G0902     ---------------------SRSLSGSTRSNNSTT----------------T---SSSS
Gh_D01G0942     ---------------------SRSLSGSTRSNNSTT----------------T---SSSS
Gh_A05G1595     ---------------------TRSLSGSTRSNNSSTT-------------YTT---SSSS
Gh_D05G1782     ---------------------TRSLSGSTRTNNSSTT-------------YTT---SSSS
Gh_A06G0349     -----------------------------------VS-------------SSN---VGSF
Gh_D06G0380     -----------------------------------VS-------------SSN---VGSF
Gh_D05G2208     -----------------SSTNSSSLL-SVSSAAPPAS-------------SSA---GGSF
Gh_A05G2001     -----------------SSTNSSSLL-SVSSAA-AAS-------------SSA---GGSF
Gh_A01G1610     ------------------------------------------------------------
Gh_D01G1850     ------------------------------------------------------------
Gh_A10G1157     SMNETGEQEETGGNGNPGTGIERNVIDIVTSNSEGITSVTESAENIVEPSVE--------
Gh_D10G1339     SMNETGEQEETGGNGNPGTGIERNVIDIVTLNSEGITSVTESAEKIVEPSVE--------

Gh_D05G0878     R-S---QIQSGYCPESGISSDYP-----FFPLRNDIFPCTACGEIFQKSHLLEQHQATKH
Gh_A05G0747     L-S---QIQSGSCPESDISSDYP-----FFPLRNDIFPCTACGEIFQKPHLLEQHQATKH
Gh_A07G0936     P----------PHPESDISSDNP-----FSPLRNDIFPCTACGEVFQKPHFLEQHQATKH
Gh_D07G1014     P----------PHPESDISSDNP-----FSPLRNDIFPCTACGEVFQKPHFLEQHQATKH
Gh_D01G0570     RKSPFLVSERKGCGGNGHSSNRV--PLDADSNGSSTVTCHKCGEQFSKWEAAETHHLSKH
Gh_A01G0562     RKSPFLVSERKGSGGNGHSSNRV--PLDADSNGSSTVTCHKCGEQFNKWEAAETHHLSKH
Gh_A05G1425     IAGPSLM--RSSCGISS-SK-----PRRSLDFESQRYVCQKCGENFKKLEAIESHHLSTH
Gh_D05G1597     IAGPSLM--RSSCGISS-SK-----PRRSLDFESQRYVCQKCGEIFKNLEAIESHHLSTH
Gh_A01G0902     RAIQF--RKLSGCYECHTIVDPS----RYPSSRTSICACSQCGEVFPEIESLELHQAVRH
Gh_D01G0942     RAIQF--RKLSGCYECHTIVDPS----RYPSSRTSICACSQCGEVFPKIESLELHQAVRH
Gh_A05G1595     RAMQF--RKLSGCYECRMIVDPS----RYPSSRTTISACSQCGEVFPKIESLELHQAVRH
Gh_D05G1782     RAMQF--RKLSGCYECRMIVDPS----RYPSSRTTISACSQCGEVFPKIESLELHQAVRH
Gh_A06G0349     RGLPF--TRFSGCYECRMVVDPVLGMAKDPSLRTTIRSCPECGEIFMKAENLELHQAVRH
Gh_D06G0380     RGLPF--TRFSGCYECRMVVDPVLGMAKDPSLRTTIRSCPECGEIFMKAENLELHQAVRH
Gh_D05G2208     GGMPF--RRFSGCYECKLVVDPLLGISKDPSLRGTICSCPECGEIFMKAENLELHQAVRH
Gh_A05G2001     GGMPF--RRFSGCYECKLVVDPLLGISKDPSLRGTICSCPECGEIFMKAENLELHQAVRH
Gh_A01G1610     ---------------------------------------IRNEAQYEYN---------DQ
Gh_D01G1850     ---------------------------------------IRNEAQYEYN---------DQ
Gh_A10G1157     ------------------VHDSVLQDKPDNALTNQLYEDIEIDASYMRV---------AK
Gh_D10G1339     ------------------VHDSVLQDKPDTALTNQLYEDIEIDASYMYV---------AK
```

```
                                                             :             :

Gh_D05G0878      AVSELVDGDSGNNIVRII--------FKTGWTDKVK-SPEIHRILKIHNSPKILTKFEEY
Gh_A05G0747      AVSELVDGDSGNNIVRII--------LKTGWTDKVK-SPEIHRILKIHNSPKILTKFEEY
Gh_A07G0936      AVSQLLDGDSGKNIVRII--------FKTGWTDKVR-NPEIHRILKIHNSTKILARFEEY
Gh_D07G1014      AVSQLLDGDSGKNIVRII--------FKTGWTDKVR-NPEIHRILKIHNSPKILARFEEY
Gh_D01G0570      AVTELVEGDSSRKIVEII--------CRTSWLKSENHCGRIERVLKVHNMQKTLARFEDY
Gh_A01G0562      AVTELVEGDSSRKIVEII--------CRTSWLKSENHCGRIERVLKVHNMQKTLARFEDY
Gh_A05G1425      AVTALSEGDSSKKIVELI--------CQTSFLEPENKFGEIERILKIHNMQRTLVQFEDY
Gh_D05G1597      AVTALSEGDSSKKIVELI--------CQTSFLEPENKFGEIERILKIHNMQRTLVQFEDY
Gh_A01G0902      AVSELGPEDSGRNIVEII--------FKSSWLKKDNPICKIERILKVHNNQRTIQRFEDC
Gh_D01G0942      AVSELGPEDSGRNIVEII--------FKSSWLKKDNPICKIERILKVHNNQRTIQRFEDC
Gh_A05G1595      AVSELGPDDSGRNIVEII--------FKSSWLKKDSPICKIERILKVHNTQRTIQRFEDC
Gh_D05G1782      AVSELGPDDSGRNIVEII--------FKSSWLKKDNPICKIERILKVHNTQRTIQRFEDC
Gh_A06G0349      AVSELGSEDTSKNIVEII--------FQSSWLKKQAPICQIDRILKVHNTPKTISKFEEY
Gh_D06G0380      AVSELGPEDTSKNIVEII--------FQSSWLKKQAPICQIDRILKVHNTPKTISKFEEY
Gh_D05G2208      AVSELGPEDTSKNIVEII--------FKSSWLKKQTPICQIDRILKVQNTPKTISKFEEY
Gh_A05G2001      AVSELGPEDTSKNIVEII--------FKSSWLKKQTPICQIDRILKVQNTPKTISKFEEY
Gh_A01G1610      EEY---------------------------------------------------------
Gh_D01G1850      EEY---------------------------------------------------------
Gh_A10G1157      DSYELG-GNHEATVKEVLFEGKAGLLQDHKWSDELSPVD-------------------AY
Gh_D10G1339      DSYELG-GNHEAIVKEVLFEGKAGLLQDHKWSDELPPVD-------------------AD


Gh_D05G0878      REVVKAKAARNGA-MGRRDERCIADGNELLRFNCSTFTC-----------D------LGL
Gh_A05G0747      REVVKAKAARNGA-MGRRDERCIADGNELLRFNCSTFTC-----------D------LGL
Gh_A07G0936      RELVKAKAARNGAVVGRRDERCIADGNELLRFYCSTFMC-----------D------LGL
Gh_D07G1014      RELVKVKAARNGAVVGRRDERCIADGNELLRFYCSTFMC-----------D------LGL
Gh_D01G0570      REIVKIKASK----LPKKHPRCIADGNELLRFYGTTVAC-----------S------LGL
Gh_A01G0562      REMVKIKASK----LPKKHPRCIADGNELLRFYGTTVAC-----------S------LGL
Gh_A05G1425      REMVKLKANK----LSKKHPRCLADGNELLRFYGSTVAC-----------S------LGM
Gh_D05G1597      REMVKLKANK----LSKKHPRCLADGNELLRFYGSTVAC-----------S------LGM
Gh_A01G0902      RDAVKTRALN----STRKNPRCAADGNELLRFHCTTLSC-----------S------LGA
Gh_D01G0942      RDAVKTRAIN----STRKNPRCAADGNELLRFHCTTLSC-----------S------LGA
Gh_A05G1595      RDAVKTLALN----STRKNARCAADGNELLRFHCTTLSC-----------S------LGA
Gh_D05G1782      RDAVKTRALN----STRKNPRCAADGNELLRFHCTTLSC-----------S------LGA
Gh_A06G0349      RDSIKSKATK----HPKKHPRCIADGNELLRFHCTTFAC-----------S------LGL
Gh_D06G0380      RDSIKSKATK----HPKKHPRCIADGNELLRFHCTTLAC-----------S------LGL
Gh_D05G2208      RDSIKSKAT-------KKHPRCIADGNELLRFHCTTFAC-----------S------LGL
Gh_A05G2001      RDSIKSKAT-------KKHPRCIADGNELLRFHCTTFAC-----------S------LGL
Gh_A01G1610      ------------------------------------------------------------
Gh_D01G1850      ------------------------------------------------------------
Gh_A10G1157      TTENEK-DQE-VGSLQEQQPVYVADDLDPTGFSGSMINDLPEGKPLVVDADIEAGKLNNV
Gh_D10G1339      STENEK-DQE-VGSLQEQQPVYVADDLDPTGFLGSMINDLPEGKPLVVDADIEAGKLNNV


Gh_D05G0878      NGSSSICNQ-QYCSICGIIKSGFSPKMD--------------------------------
Gh_A05G0747      NGSSSICNQ-QYCSICGIIKSGFSPKMD--------------------------------
Gh_A07G0936      NGSSGICSQ-QYCSVCGIIKSGFSPKMD--------------------------------
Gh_D07G1014      NGSSGICSQ-QYCSVCGIIKSGFSPKMD--------------------------------
Gh_D01G0570      NGSSSLCIS-EKCCVCRIIRNGFSAKKE--------------------------L-----
Gh_A01G0562      NGSSSLCIS-EKCCVCRIIRNGFSAKKE--------------------------L-----
Gh_A05G1425      ENTSSLCTL-EQCEVCQILRQGFFSKRE--------------------------A-----
Gh_D05G1597      ENTSSLCTL-DQCEVCQILRQGFFSKKE--------------------------A-----
Gh_A01G0902      RGSSSLCGSIPGCGVCTIIRQGFQNKGE---------------------------GT---
Gh_D01G0942      RGSSSLCGSIPGCGVCTIIRQGFQNKGG---------------------------GT---
Gh_A05G1595      RGSSSLCGSIPGCGVCTIIKQGFQKKGG---------------------------GV---
Gh_D05G1782      RGSSSLCGSIPGCGVCTIIKQGFQKKGG---------------------------GA---
Gh_A06G0349      NGSSNLCNSSPNCNVCSIIKNGFKVAQE--------------------------LGN---
Gh_D06G0380      NGSSNLCNSSPNCNVCSIIKNGFKVAQE--------------------------LGN---
Gh_D05G2208      SGSTNLCNTIPKCNVCSIIKNGFKVAQE--------------------------------
Gh_A05G2001      SGSTNLCNTIPKCNGCSIIKNGFKVAQE--------------------------------
Gh_A01G1610      ------------------------------------------------------------
```

```
Gh_D01G1850      ------------------------------------------------------------
Gh_A10G1157      VGEDVICIP--DDNLGRIDDQTYVKTSSFESMNNSSPSHTNPASNLLEVDNSDDIGEKKT
Gh_D10G1339      VGEDVICIP--NDNLGRIDDQTYVKNSSCESMNNSSPSHTNPASNLLEVDNSDDIGEKKT

Gh_D05G0878      ------------GISTL------------------------STSWRAHMAIPEDVEEEFK
Gh_A05G0747      ------------GISTL------------------------STSWRAHMAIPEDVEEEFK
Gh_A07G0936      ------------GISTL------------------------STSWRAHMAVPEDVEEEFK
Gh_D07G1014      ------------GISTL------------------------STSWRAHMAVPEDVEEEFK
Gh_D01G0570      --------KEGLGVFTT------------------------STSGRAFESIQILEDD---
Gh_A01G0562      --------KEGLGVFTT------------------------STSGRAFESIQILEDD---
Gh_A05G1425      --------NGCWGVFTS------------------------STSKRAMESVELDKEK---
Gh_D05G1597      --------NGCWGVLTS------------------------STSKRAMESVELDKEQ---
Gh_A01G0902      -----APAAEFKGVVTT------------------------ASSGRAHDSIKR-------
Gh_D01G0942      -----APAAEFKGVVTT------------------------ASSGRAHDSIKR-------
Gh_A05G1595      -----AAAAEFKGVCTT------------------------ASSGRAHDSLKC-------
Gh_D05G1782      -----AAAAEFKGVCTT------------------------ASSGRAHDSLKC-------
Gh_A06G0349      -----GGGPNGKGILTT------------------------ATSGKAHDMAAAVEED--N
Gh_D06G0380      -----GGGPNGKGILTT------------------------ATSGKAHDMAAGVEED--N
Gh_D05G2208      --------TNGKGILTT------------------------ATSGKAHDKAAGVED----
Gh_A05G2001      --------TNGKGILTT------------------------ATSGKAHDKAAGVED----
Gh_A01G1610      ------------------------------------------------------------
Gh_D01G1850      ------------------------------------------------------------
Gh_A10G1157      EKHDINVVESGDGIEDLIKANSTSESISTGNQSPDVIEEVNETKGPHLDMVSDREDEELE
Gh_D10G1339      KKHDISVVE------------------------------------------SDREDEELE

Gh_D05G0878      F-----------------------------------------------------------
Gh_A05G0747      F-----------------------------------------------------------
Gh_A07G0936      F-----------------------------------------------------------
Gh_D07G1014      F-----------------------------------------------------------
Gh_D01G0570      ------------------------------------------------------------
Gh_A01G0562      ------------------------------------------------------------
Gh_A05G1425      ------------------------------------------------------------
Gh_D05G1597      ------------------------------------------------------------
Gh_A01G0902      ------------------------------------------------------------
Gh_D01G0942      ------------------------------------------------------------
Gh_A05G1595      ------------------------------------------------------------
Gh_D05G1782      ------------------------------------------------------------
Gh_A06G0349      G-----------------------------------------------------------
Gh_D06G0380      G-----------------------------------------------------------
Gh_D05G2208      G-----------------------------------------------------------
Gh_A05G2001      G-----------------------------------------------------------
Gh_A01G1610      ------------------------------------------------------------
Gh_D01G1850      ------------------------------------------------------------
Gh_A10G1157      LSMDYKIR-EGASEDLMSFAVDNNGGGNGSERTSIDQSKKELMHSLSYSNPTSQNSGAVD
Gh_D10G1339      LSMDYKIQGEGAGEDLVSFAVDNNGGGNGFERTSIDQSKKELMHSLSYSNPTSQNSGAVD

Gh_D05G0878      -----MNVKRAMLVCRVV-AGRVGSEGEE--IDKE-----------DGGFDSVIGRGGGS
Gh_A05G0747      -----MNVKRAMLVCRVV-AGRVGSEGEE--IDKE-----------DGGFDSVIGRGGGS
Gh_A07G0936      -----MNVKRAMLVCRVV-AGRVGSEGDE--TNKE-----------DGGFDSVIGRGGGN
Gh_D07G1014      -----MNVKRAMLVCRVV-AGRVGSEGDE--TNKE-----------DGGFDSVIGRGGGN
Gh_D01G0570      -----PSIRKALIVCRVI-AGRVHRPLEN--IQEMAGQ---------TGFDSLAGKVGL-
Gh_A01G0562      -----PSIRKALIVCRVI-AGRVHRPLEN--IQEMAGQ---------TGFDSLAGKVGL-
Gh_A05G1425      -----HSLRKALVVCRVI-AGRVVKGLEK--MREMEGQ---------SSFDSVAGKADC-
Gh_D05G1597      -----HSLRKALVVCRVI-AGRVVKGLEK--MREMEGQ---------SSFDSVAGKADC-
Gh_A01G0902      -----TDGRRAMLVCRVI-AGRVKRVADE--APPLEDENSSGVSSSAGSYDSVAAYAGV-
Gh_D01G0942      -----TDGRRAMLVCRVI-AGRVKRVADE--APPLEDENSSGVSSSAGSYDSVAAYAGV-
Gh_A05G1595      -----TDGRRAMLVCRVI-AGRVKRVSED--APPFEEDNSGGVAAATGLYDSLAAYAGV-
Gh_D05G1782      -----TDGRRAMLVCRVI-AGRAKRVTED--APPFEEDNSGVAAAATGSYDSLAAYAGV-
Gh_A06G0349      -----KQENRAMLVCRVI-AGRVKKNMEG----SLEDYDSVT---------VAGGDVGA-
Gh_D06G0380      -----KQENRAMLVCRVI-AGRVKKNMEG----SLEDYDSVT---------VAGGDVGA-
```

```
Gh_D05G2208      -----DYEKRAMLVCRVI-AGRVKKNIME--GDNMEEYDS------------VAGAVGV-
Gh_A05G2001      -----DYEKRAMLVCRVI-AGRVKKNIME--GDNLEECDS------------VAGAVGV-
Gh_A01G1610      ------------------------------------------------------------
Gh_D01G1850      ------------------------------------------------------------
Gh_A10G1157      DNHTRESGLDAFGTSTVILQGEADIGPIKPQPDTTIGDVSIGSSSH---TDSLEAHWGSI
Gh_D10G1339      DNHTRESGLDAFGTSTVILQGEADNGPIKPQPDTTVGDVSIGSSSH---TDSLEAHWGSI


Gh_D05G0878      GA-Y----TKF--------------DEEELLVFNPRAVLPCFVIVYTV------------
Gh_A05G0747      GA-Y----TKF--------------DEEELLVFNPRAVLPCFVIVYTV------------
Gh_A07G0936      MVHH----TKV--------------DEEELLVFNPRAVLPCFVIVYTV------------
Gh_D07G1014      MAHH----TKV--------------DEEELLVFNPRAVLPCFVIVYTV------------
Gh_D01G0570      ---------YS--------------NIEELYLLNPRALLPCFVVICKP------------
Gh_A01G0562      ---------YS--------------NIEELYLLNPRALLPCFVVICKP------------
Gh_A05G1425      ---------HS--------------DIEELYSLSPRALLPCFILIICKN-----------
Gh_D05G1597      ---------HS--------------NIEELYSLSPRALLPCFILIICKN-----------
Gh_A01G0902      ---------YS--------------NLEELVVFNPRAILPCFVVIYKALESGV-------
Gh_D01G0942      ---------YS--------------NLEELVVFNARAILPCFVVIYKALECGL-------
Gh_A05G1595      ---------YS--------------NLEELVVFNPRAILPCFVVIYKAHES---------
Gh_D05G1782      ---------YS--------------NLEELVVFNPRAILPCFVVIYKAHES---------
Gh_A06G0349      ---------YS--------------NLDELYVFNPKAILPCFVVIYRGF-----------
Gh_D06G0380      ---------YS--------------NLDELYVFNPKAILPCFVVIYRGF-----------
Gh_D05G2208      ---------YS--------------DLDELFVFNPNAILPCFVVIYRGF-----------
Gh_A05G2001      ---------YS--------------DLDELFVFNPNAILPCFVVIYRGF-----------
Gh_A01G1610      ------------------------------------------------------------
Gh_D01G1850      ------------------------------------------------------------
Gh_A10G1157      SVLSTQSDAFSKAEKTKKSKAVSKQHFDTSDEFE----PPSFMTLVEPGGSNEKSTFSEI
Gh_D10G1339      SVLSTQSDAFSEAEKTKKSKAVSKQHFDTSDEFE----PPSFMTLVEPGGSNEKSTISDI
```

Subgroup 4

```
Gh_D10G0401          MNFHSGREDAQKHAVDKSCIYCNKVFDDYLALSGHLRIHEEE-TLRALNFPGRSSNSIDI
Gh_Sca004883G01      MNFHSGRKDAQKHAVDKSCIYCNKVFDDYLALSGHLRIHEEE-TLRALNFPGRSSNSIDI
Gh_A09G0743          MNFLLPRDDSEEHLGTKTCFYCNKVFSSHRALGGHLRIHQDGRILRAPNYPGRSRSSVNN
Gh_D09G0744          MNFLLPRDDSEEHAGTKTCFYCNKVFSSHRALGGHLRIHQDGSILRALNYPGGSSSSVNN
                     ***   *.*:::*   *:*:******..: **.******::   *** *:** * .*::

Gh_D10G0401          TRNPPAPLPNSQLSLA-SVNNLTPITR---------------------------------
Gh_Sca004883G01      TRNPPAPLPNSQLSLA-SVNNLTPITR---------------------------------
Gh_A09G0743          TGNPPASLPNSELNSFASFNSLSAISSNSVNNSGNPPASLPNSQLNSSSSFNSPTQFSIS
Gh_D09G0744          TGNPPASLPNSQLNSFASFNSLSAITSNSVNNSGNPPASLPNSQPNSSSSFNSPTQFSTS
                     * **** ****:*.   *.*.*: *:

Gh_D10G0401          ------------------------------TPTLIDFCAMFGSCDANQANRSKSTGSNLG
Gh_Sca004883G01      ------------------------------APTLIDFCAMFGSCDANQANRSKSTGSNLG
Gh_A09G0743          SVNNSGNSPASLPNNQLNSTAFNSLTLLPAAHLACDFSWMIYLNETNQVSRSQFIGGYSG
Gh_D09G0744          SVNNSGNSPASLPNNQLNSTAFNSLTLLPAAHLACDFSWMIYLNETNQVSRSQFIGGYSG
                                                   :    **. *:   ::**..**:  *.  *

Gh_D10G0401          DAQTQIVMSP-------SGATFRHNSSA-----------------------------SKT
Gh_Sca004883G01      DAQTQIVMSP-------SGATFRHNSSA-----------------------------SKT
Gh_A09G0743          VPLIPVILSPDFSYGYSYGATYHHNSLAPRAFAPVGSNVDFPYVCSSCAAYYHNILALRA
Gh_D09G0744          VPLIPVILSPDFSYGYSYGATYHHNSLASRAFAPVGSNVDFPYVCSSCAAYYHNILASRA
                          :::**        ***::*** *                              ::

Gh_D10G0401          FAPVGANAALSSAACASSGGVVATGLPTDSSSYLRKYEVCQFNTDEFQISQDGLPPASRD
Gh_Sca004883G01      FAPVGANAALSSAACASSGGIVATGLPTDSSSYLRKYEVCQFNTDEFQISQDGLPPASRD
Gh_A09G0743          FVPVGMIAAMPSAAFALCGSVVAPGFLVDSSLFLGSNGVRQFNTNEFQISQVTLSPSFGN
Gh_D09G0744          FVPVGMIAAMPSAAFALFGSVVAPGFLVDSSLFLGSNGVRQFNTNEFQVSQVTLSPSFGN
                     *.***  **: *** *  *.:** *: .*** :* .  * ****:***:**  * *:  :
```

```
Gh_D10G0401          AMQKTQGYNLGKLPNPTFAITNPDLGCESYQLPRSNGSIASLWLLPGPPPSSYVGKVGQS
Gh_Sca004883G01      AMQKTQGYNLGKLPNPTFAITNPDLGCESYQLPRSNGSIASSWLLPGPPPSSNVGKVGQS
Gh_A09G0743          ALPNVQAQNAG-------------------------------------PPSAVSANVGRS
Gh_D09G0744          ALPNVQAQNAG-------------------------------------PPSAGSANVGRS
                     *: :.*. * *                                     ***:  .:**:*

Gh_D10G0401          ETILLMGEGSKRPCLADNPMTASLMNASKKPKISPSALEEPKKLQIKELPLLKKLEDSLS
Gh_Sca004883G01      ETILLMGEGSKRPCLADNPMTASLMNASKKPKISPNALEEPKKLQIKELPLLKKLEDSLS
Gh_A09G0743          ERRSL-G---KRSREADRSRNAETSNASRRPRIAPNEHVEQENRPKKELQLFVDVVVPSS
Gh_D09G0744          ERRSS-G---KRSREADRSRNAETSNASRRPRIAPNEHVEPENCPKKELQLFVDVVVPSS
                     *     *   **   **.  .*.  ***::*:*:*.   * ::   *** *: .:    *

Gh_D10G0401          ALETCVGAEEEGPVDLDLSLHL
Gh_Sca004883G01      ALETCVGAEEEGPVDLDLSLHL
Gh_A09G0743          SSEASSSAEEEDPVDMDLSLHL
Gh_D09G0744          SSEASSSAEEEDLVDMDLSLHL
                     : *:. .****  **:******
```

Subgroup 5

```
Gh_D01G1111       AETIKCHKCTMTFNLQDDFICHLLSSHKGSA---------------------------MS
Gh_A01G1056       AETIKCHKCTMTFNLQDDFICHLLSSHKGSA---------------------------MS
Gh_D07G1723       QGSS---SIKRGSTT------DVAIQIGSGS-GS--------------GSN---------
Gh_A07G1555       QGSS---SRKRGSTI------DVAIQIGSGS-GS--------------GSN---------
Gh_A08G0095       GGQHVCPVCSKGFTSGKALGGHVRIHMKANKNGGRYKKISKRQPRNNNNNNIRKKIPIMA
Gh_D08G0137       GGQHVCPVCSKGFTSGKALGGHVRIHMKANKNGGRYKKISKRQPRNNNNNNIRKKIPIMA
Gh_A07G1084       CNQHICSVCSKGFTSGKALGGHIRIHMKGNNLDLHSRASKE-------KKRISKN-----
Gh_D07G1182       CNQHICSVCSKGFTSGKALGGHIRIHMKGNNLDLHSRASKE-------KKRISKN-----
                           .   .       .:     .

Gh_D01G1111       SGHGTPATEEVKIKNGKYECQFCHQLFEERNCYSSHLGIHMENNMKKVEGSVGEQNTVQP
Gh_A01G1056       SGHGTPATEEVKIKNGKYECQFCHQLFEERNCYSSHLGIHMENNMKKVEGSVGEQNTVQP
Gh_D07G1723       ----ARKKREMEIPRGEPTCYVCSKNFTSWKAVFGHLKSHQRETPGALPPP---------
Gh_A07G1555       ----ARKKREMEIPRGEPTCYVCSKNFTSWKAVFGHLKSHQRETPGALPPP---------
Gh_A08G0095       AVAAAETPHESHGSEEKVSCCICKKDFKSLKSLFGHMRNHPERNWRGIRPP---------
Gh_D08G0137       AVAAAETPHESHGSEEKVSCCICKKDFKSLKSLFGHMRNHPERNWRGIRPP---------
Gh_A07G1084       ----IDDDAVDDQAEDKVSCCVCNKDFKSMKSLFGHMRNHPNRTWRGIRPP---------
Gh_D07G1182       ----IDDDAVDDQAEDKVSCCVCNKDFKSMKSLFGHMRNHPNRTWRGIRPP---------
                             .  . :  * .* : * . :.  .*:  * ...   :

Gh_D01G1111       LNSAGSNEIGPGFRCSESNENALVETF-TDKYNHEGSLLSNDEQDKVNMNEKVLADRNCD
Gh_A01G1056       LNSAGSNEIGPGFCCSESNENALVETF-TDKYNHEGNLLSRDEQDKVNMNEKVLADRNCD
Gh_D07G1723       -------TFTPTEGSPENNNDDETNPREQLAPTLLNLALE---------------TMQKM
Gh_A07G1555       -------TFTPTEGSPENNNDDETNPREQLAPTLLNLALE---------------TMQKM
Gh_A08G0095       -------PSDRNSCCSSVSENDEAVVVDQIKGSAS-DLLK---------------SLPKW
Gh_D08G0137       -------PSDRNSCCSSVSENDEAVVVDQIKGSAS-DLLK---------------SLPKW
Gh_A07G1084       -------PLEKNSCCSTVSEEDEALENDQVSFARDENFVS---------------SLPKR
Gh_D07G1182       -------PLEKTSCCSTVSEEDEALENDQVSFTRDENFVS---------------SLPKR
                                .   .::  .              :.

Gh_D01G1111       TKSKFCFVTDNKGDITDATAAADLNVCLGSENILFTSDKEGISRPSDKIDVGFAVNSME-
Gh_A01G1056       TKIKFCFVTDNKGDITDATAAADLNVCLGSESILFTSDKEGISRPSDKIDVGFAVNSME-
Gh_D07G1723       SEDSN---------------------------------MSVVV-------AGEEASSSG-
Gh_A07G1555       SEDSN---------------------------------MSVVV-------AGEEASSSG-
Gh_A08G0095       TTTTKRFEKSTRSDHANN---ADADTDTDTEDEITEAAYCLMKLSRGNSF---E--LDNT
Gh_D08G0137       TTTTKRFEKSTRSDHANN---ADAD--TDTEDEITEAAYCLMKLSRGNSF---E--IDNT
Gh_A07G1084       SNSAKC-------------------CWNSDEEEIFEAAYSLMKLSRDSFGVGYQTKSSPT
Gh_D07G1182       SNTAKR-------------------CWNSDEEEIFEAAYSLMKLSRDSFGVGYQTKSSPT
                  :                                        :

Gh_D01G1111       -------------------------------------------------EKKREMASNTS
Gh_A01G1056       -------------------------------------------------EKKREMALNTS
Gh_D07G1723       ------------KGRGRGKGRGRGR--------------------GRRGLDIDLNQPKTS
Gh_A07G1555       ------------KGRGRGKGRGRGR--------------------GRRGLDIDLNQPKTS
```

```
Gh_A08G0095       -------------------------------------------KIHKTP-------LKTS
Gh_D08G0137       -------------------------------------------KIHKTP-------LKTS
Gh_A07G1084       TIHETPSWGLPNKASLEGKGKLLPKELNCDESAEIIENNSCFDQFHKFPVKISS-SQNFG
Gh_D07G1182       TIHETPSWGLPNKASLEGKGKLLAKELNCDESAEIIENNSCFDQFHKFPVTISS-SQNFG
                                                                           : .

Gh_D01G1111       FLAPNAKGNM--------FTDENTEDGHF-PSFLKGMEVDLKDKATRDDPKAGCADTSTE
Gh_A01G1056       FLAPNAKGNM--------FSDENTEDGHF-PSFLKGMEVDLKDKATRDDPKAGCADTSTE
Gh_D07G1723       FLLDLNEPPPPDQDDDDEDDDDDDDDGA--------------------------------
Gh_A07G1555       FLLDLNEPPPPEQDDDDDYDDDDDDGAA--------------------------------
Gh_A08G0095       FYTKNPKTTPLKEEAKQYYTKNN---------KTQQLDFDLNEP----------------
Gh_D08G0137       FYTKNPKTTPLKEEAKQYYTKNN---------KTQQLDFDLNEP----------------
Gh_A07G1084       FYLKNPSGPPAVVEGKHLYSASEAGEGSHQVCSRKLLDVDLNEP----------------
Gh_D07G1182       FYLKNPSGPPAVVEGKHLYSASEAGEGSHQVWSRKLLDVDLNEP----------------
                  *     .              .:

Gh_D01G1111       LSNVRIDNVQGNYSEGCSLIPSGNKQRVNLVDHLKGASVTTDSTHERGSGCGLNSSKGDQ
Gh_A01G1056       LSNVRIDNVQGNYSEGCSLIPSGNKQRVNLVDHLKGASVTIDSTHERGSGCGLSSSKGDQ
Gh_D07G1723       ------------------------------------------------------------
Gh_A07G1555       ------------------------------------------------------------
Gh_A08G0095       ------------------------------------------------------------
Gh_D08G0137       ------------------------------------------------------------
Gh_A07G1084       ------------------------------------------------------------
Gh_D07G1182       ------------------------------------------------------------


Gh_D01G1111       TCVINNSLILVSGTLDDPESIMVNESANIDPTICFQSHLPMKKPSQEKSETVLLTSHGRE
Gh_A01G1056       TCVISNSLILVSGTLDDPESIMVNESANIEPTICFQSHLPMKKPSQEKSETVLLTSHGRE
Gh_D07G1723       ------------------------------------------------------------
Gh_A07G1555       ------------------------------------------------------------
Gh_A08G0095       ------------------------------------------------------------
Gh_D08G0137       ------------------------------------------------------------
Gh_A07G1084       ------------------------------------------------------------
Gh_D07G1182       ------------------------------------------------------------


Gh_D01G1111       QIFPSDNNAFKAFSRTVEVSELDGAQNYRGLSPGVNSRNSGVDPNILASFKHEKTKDRLF
Gh_A01G1056       QIFPSDNNAFKAFSRTVEVSDLDGAQNYRGLSPGVNSRNSGVDPNILASVKHEKTKDRLF
Gh_D07G1723       -----------------------------AADDDENDKN---------------------
Gh_A07G1555       -----------------------------ADEDDKN------------------------
Gh_A08G0095       ---------------------------YIGEDSDDSDNEV--------------------
Gh_D08G0137       ---------------------------YIGEDSDDSDSEV--------------------
Gh_A07G1084       ---------------------------YVGVDHDLT------------------------
Gh_D07G1182       ---------------------------YVGVDYDLT------------------------
                                               . .   .
```

Subgroup 6

```
Gh_A06G0249           ------VLCWYADLY----------------------------ALFVSIRIMETNS----
Gh_D02G2008           ------SLAWYFFLNFL----------------NLQ-----DFFLRASIQAIEANS----
Gh_A03G1559           ---------------MM----------------LCL-----PINRRASIQAIEANS----
Gh_A01G1676           ------------------------------------------------------------
Gh_D01G1926           ------------------------------------------------------------
Gh_A09G1153           ------------------------------------------------------------
Gh_D09G1159           ------------------------------------------------------------
Gh_D11G2046           ------------------------------------------------------------
Gh_A11G1928           ------------------------------------------------------------
Gh_A06G0175           ------------------------------------------------------------
Gh_D06G0169           ------------------------------------------------------------
Gh_D12G1804           --------------------------------------------VPDLFSFF--------
Gh_Sca005786G01       --------------------------------------------VPDLFSFF--------
Gh_A03G0820           ------------------------------------------LEVPKVAKES--------
Gh_D02G2408           ------------------------------------------LEVPKVAKES--------
Gh_D07G1883           ----------------------------------------MDFELRRAREKLDKEQRERK
```

```
Gh_A07G1674          ----------------------------------------MDFELRRAREKLDKEQRERK
Gh_D03G0179          ------DRKQYFVIFFP----------------HTRNYSWADMLLVRSISEFPQPIAYRS
Gh_A02G1540          ------DRKQYFVIFFP----------------HTRNYSWADMLLVRSISEFPQPIAYRS
Gh_D03G0180          ------DRKQYFVVFFP----------------HTRNYSWADMILVRPINEFPQPIACRS
Gh_A02G1539          ------DRKQYFVVFFP----------------HTRNYSWADMIFVRPINEFPQPIAYRS
Gh_D13G1910          ------------------------------------------MDYRRYNSSQGANLGTKQ
Gh_A13G2297          ------------------------------------------MDYRRYNSSQEANLGTKQ
Gh_A09G0878          ------------------------------------------MEFWGIEVKAGQPIKT--
Gh_D09G0901          ------------------------------------------MEFWGIEVKAGQPIKT--
Gh_D05G0849          --------------------------------------NHLVILLLGIEVKSGQPIKA--
Gh_A05G0702          ------------------------------------------MEFWGIEVKSGQPIKA--
Gh_D01G2032          ------------------------------------------MEFWGAEVKSGQNFEVEL
Gh_D11G3254          ------------------------------------------MEFWGAEVKSGQSFEVEL
Gh_A11G2870          ------------------------------------------MEFWGAEVKSGQSFEVEL
Gh_D05G1175          ------------------------------------------------------------
Gh_A05G3867          ------------------------------------------------------------
Gh_A05G2341          ------------------------------------------------------------
Gh_D05G2606          ------------------------------------------------------------
Gh_A12G0454          ------------------------------------------------------------
Gh_D12G0457          ------------------------------------------------------------
Gh_A03G1535          ENRSIISSLKVSLKSHPEFQHLDFTTPPSSPRDAIVSYGMRDTMVDFEIKDAMISLGKRK
Gh_D01G1781          ------------------------------------------------------------
Gh_A01G1532          ------------------------------------------------------------
Gh_A10G0992          ------------------------------------------------------------
Gh_D10G2570          ------------------------------------------------------------
Gh_A06G0337          ------------------------------------------------------------
Gh_D06G0368          ------------------------------------------------------------
Gh_D05G3801          ------------------------------------------------------------
Gh_A05G1995          ------------------------------------------------------------
Gh_D07G0542          ------------------------------------------------------------
Gh_A07G0477          ------------------------------------------------------------
Gh_A05G2985          ------------------------------------------------------------
Gh_D04G0776          ------------------------------------------------------------
Gh_D05G0663          AM----------------------------------------------------------
Gh_A05G3915          AM----------------------------------------------------------
Gh_D07G0016          TM----------------------------------------------------------
Gh_A07G0010          TM----------------------------------------------------------
Gh_D12G2446          ------------------------------------------------------------
Gh_A12G2312          ------------------------------------------------------------
Gh_D08G1069          ------------------------------------------------------------
Gh_A08G2455          ------------------------------------------------------------
Gh_D07G2319          ------------------------------------------------------------
Gh_A07G2108          ------------------------------------------------------------
Gh_A05G2549          ELLRSKGGAKMLIICHSDYQKIKANAIPVADD-IGIPFNYNDVPLDAASEE---------
Gh_D05G2825          ELLQSKGGAKMLVICHSDYQKIKANAIPVADD-IGIPFNYNDVPLDAASEE---------
Gh_A07G2281          QQLRQIGGVQVFLLCHPEYPRIEAEAKLVAEE-LGIDYPWNDILFGDATKD---------
Gh_D07G0545          QQLRQIGGVQVFLLCHPEYPKIEAEAKLVAEE-LGIDYPWNDILFGDATKD---------
Gh_D09G2376          QQLRQIGGVHIFLLCHPEYPRIEAEAKLVAEE-LGINYLWNNILFGDATKD---------
Gh_A09G2168          QQLRQIGGVHIFLLCHPEYPKIEAEAKLVAEE-LGIDYLWNNILFGDATKD---------

Gh_A06G0249          ---SLKNQQCL----------------TYWVLFAFITMVELTLGN-----ILKWFP-FWP
Gh_D02G2008          ---NTDTKKLV----------------TYWIIFSLISLFEHAFMG-----ILQWLP-FWP
Gh_A03G1559          ---NTDTKKLV----------------TYWIIFSLISLFEHAFMG-----ILQWLP-FWP
Gh_A01G1676          ------------------------------------------------------------
Gh_D01G1926          ------------------------------------------------------------
Gh_A09G1153          ------------------------------------------------------------
Gh_D09G1159          ------------------------------------------------------------
Gh_D11G2046          ------------------------------------------------------------
Gh_A11G1928          ------------------------------------------------------------
Gh_A06G0175          ------------------------------------------------------------
Gh_D06G0169          ------------------------------------------------------------
Gh_D12G1804          ------------------------------------------------------------
```

```
Gh_Sca005786G01     ------------------------------------------------------------
Gh_A03G0820         ------------------------------------------------------------
Gh_D02G2408         ------------------------------------------------------------
Gh_D07G1883         ERAKLKLDRE--------------------------------------------------
Gh_A07G1674         ERAKLKLDRE--------------------------------------------------
Gh_D03G0179         HKVGLKMVRDLTVARRYIQQKLAVGMLNIIDQFHVEALI-EAARN-----VVVWKD-FAM
Gh_A02G1540         HKVGLKMVRDLTVARRYIQQKLAVGMLNIIDQFHVEALI-EAARN-----VVVWKD-FAM
Gh_D03G0180         HKVGLKMVRDLTVARRYIQQKLAVGMLNIIDQFHCEALI-ETARN-----VVVWKE-FAM
Gh_A02G1539         HKVGLKMVRDLTVARLYIQQKLAVGMLNIIDQFHCEALI-ETARN-----VVVWKE-FAM
Gh_D13G1910         ---------QISEEQAY-------------QDSLVEDL----------------------
Gh_A13G2297         ---------QISEEQAY-------------QDSLVEEL----------------------
Gh_A09G0878         DPGASH---VIHLSQATLGES--------KNK----------------------------
Gh_D09G0901         DPGASH---VIHLSQATLGES--------KNK----------------------------
Gh_D05G0849         DPGANY---VIHLSQASLGES--------KNK----------------------------
Gh_A05G0702         DPGANY---VIHLSQASLGES--------KNK----------------------------
Gh_D01G2032         EDDGNR---ILHLSQVALGEG--------TGDNKKEKG----------------------
Gh_D11G3254         EDDGSR---ILHISQAALGEV--------TSDNKKEKG----------------------
Gh_A11G2870         EDDGSR---ILHISQAALGEV--------TSDNKKEKG----------------------
Gh_D05G1175         --------------------------------------------------------MDE-
Gh_A05G3867         -----------------------------------------------------CNQMDE-
Gh_A05G2341         ----------------------------------------------------------M-
Gh_D05G2606         ------------------------------------------------------------
Gh_A12G0454         ------------------------------------------------------------
Gh_D12G0457         ------------------------------------------------------------
Gh_A03G1535         RGRKPNVQ-AV-------NSE-----------EERERGLEIVNKNGVAVDLVALGGLEDP
Gh_D01G1781         --------------------------------------------------------MEMQ
Gh_A01G1532         --------------------------------------------------------MEMQ
Gh_A10G0992         ------------------------------------------------------------
Gh_D10G2570         ------------------------------------------------------------
Gh_A06G0337         ------------------------------------------------------------
Gh_D06G0368         ------------------------------------------------------------
Gh_D05G3801         ------------------------------------------------------------
Gh_A05G1995         ------------------------------------------------------------
Gh_D07G0542         ------------------------------------------------------------
Gh_A07G0477         ------------------------------------------------------------
Gh_A05G2985         ------------------------------------------------------------
Gh_D04G0776         ------------------------------------------------------------
Gh_D05G0663         ------------------------------------------------------------
Gh_A05G3915         ------------------------------------------------------------
Gh_D07G0016         ------------------------------------------------------------
Gh_A07G0010         ------------------------------------------------------------
Gh_D12G2446         ------------------------------------------------------------
Gh_A12G2312         ------------------------------------------------------------
Gh_D08G1069         ------------------------------------------------------------
Gh_A08G2455         ------------------------------------------------------------
Gh_D07G2319         ------------------------------------------------------------
Gh_A07G2108         ------------------------------------------------------------
Gh_A05G2549         --DLNLINFAI-------DDE-----------HD--------------------------
Gh_D05G2825         --DLNLINFAI-------DDE-----------HD--------------------------
Gh_A07G2281         --DKERIQSAL-------DSE-----------DS----I---------------------
Gh_D07G0545         --DKERIQSAL-------DSE-----------DA----I---------------------
Gh_D09G2376         --DEVRIRSAL-------DSE-----------DA----I---------------------
Gh_A09G2168         --DEVRIRSAL-------DSE-----------DA----I---------------------


Gh_A06G0249         YAKGVATILL---VTPYFGGASY-VFSLLIRPYFIEKRW---------------------
Gh_D02G2008         YMKLTIVCWM---MIPHFDGAFY-VYNHFVHPCLYMDLP---------------------
Gh_A03G1559         YMKLTIVCWM---MIPHFDGAFY-VYNRFVHPCLYMDLP---------------------
Gh_A01G1676         ------------------------------------------------------------
Gh_D01G1926         ------------------------------------------------------------
Gh_A09G1153         ------------------------------------------------------------
Gh_D09G1159         ------------------------------------------------------------
Gh_D11G2046         ------------------------------------------------------------
```

```
Gh_A11G1928          ------------------------------------------------------------
Gh_A06G0175          ------------------------------------------------------------
Gh_D06G0169          ------------------------------------------------------------
Gh_D12G1804          ----SNTVYS---GYSDSGRGFYKVYSDLFSKIYANEIN---FARKL-------------
Gh_Sca005786G01      ----SNTVYS---GYSDSGRGFYKVYSDLFSKIYANEIN---FARKL-------------
Gh_A03G0820          ----EEAIAA---GSTGEPEE--MVVPE--------------------------------
Gh_D02G2408          ----EEAIAA---GSTSEPEE--MVVPE--------------------------------
Gh_D07G1883          ------------------------------------------------------------
Gh_A07G1674          ------------------------------------------------------------
Gh_D03G0179          ----EASRCN---GYSDLGKMLLKLQTMILP-CYINAEW---LQESLHLWVQQCQNAHSA
Gh_A02G1540          ----EASRCN---GYSDLGKMLLKLQTMILP-CYINAEW---LQESLHSWVQQCQNAHSA
Gh_D03G0180          ----EASRCN---GYSDLGKMLLKLQSMILP-RFINADW---LQESFHSWVQQCQNAHTA
Gh_A02G1539          ----EASRCN---GYSDLGKMLLKLQSMILP-SYINADW---FQESFHSWVQQCQNAHTA
Gh_D13G1910          --------------------------AEDFR-LPINHKP----TENV--DLDNVQQATLD
Gh_A13G2297          --------------------------AEDFR-LPINHKP----TENV--DLDNVQQATLD
Gh_A09G0878          --------------------------AESVP-LYVNVNGKKLVLGTL--SHQNCPQLCFD
Gh_D09G0901          --------------------------AESVP-LYVNVNGKKLVLGTL--SHQNCPQLCFD
Gh_D05G0849          --------------------------AESVP-LYVNVDGKKLILGTL--SHQSCPQLSFD
Gh_A05G0702          --------------------------AESVP-LYVNVDGKKLILGTL--SHQSCPQLSFD
Gh_D01G2032          --------------------------KETIC-LYLKFKNEKFVVGTL--SQEKCPQIALD
Gh_D11G3254          --------------------------NGTAC-VYLKFNNEKFVIGTL--SHDKFPQMPLD
Gh_A11G2870          --------------------------NGTAC-IYLKFNNEKFVIGTL--SHDKFPQMPLD
Gh_D05G1175          ---E---------KGERVSGPIF---------KDIRRYFCD----HCGICRSKK----SL
Gh_A05G3867          ---E---------KGERVSGPMF---------KDIRRYFCD----HCGICRSKK----SL
Gh_A05G2341          ---E---------EEKKVGGAIF---------KDIRRYYCE----FCGICRSKK----SL
Gh_D05G2606          ---M---------EEEKVGGAIF---------KDIRRYYCE----FCGICRSKK----SL
Gh_A12G0454          ------------------------------------------------------------
Gh_D12G0457          ------------------------------------------------------------
Gh_A03G1535          YAEELKRRTEGMAGSEEALLGFM---------RDLGGQWCS-------RRRKRK-----I
Gh_D01G1781          YAHGLFERRPLLKS------------------KAPAVKWFK-------RWVPQD-----V
Gh_A01G1532          YAHGLFERRPILKS------------------KAPAVKWFK-------RWVPQD-----V
Gh_A10G0992          ------------------------------------------------------------
Gh_D10G2570          ------------------------------------------------------------
Gh_A06G0337          ------------------------------------------------------------
Gh_D06G0368          ------------------------------------------------------------
Gh_D05G3801          ------------------------------------------------------------
Gh_A05G1995          ------------------------------------------------------------
Gh_D07G0542          ------------------------------------------------------------
Gh_A07G0477          ------------------------------------------------------------
Gh_A05G2985          ------------------------------------------------------------
Gh_D04G0776          ------------------------------------------------------------
Gh_D05G0663          ---------------------------------------RT----KVGMKNNVIRNLPKN
Gh_A05G3915          ---------------------------------------RT----KVGMKNNVIRNLPKN
Gh_D07G0016          ---------------------------------------RT----KVGMKNNVIQNLPKN
Gh_A07G0010          ---------------------------------------RT----KVGMKNNVIQNLPKN
Gh_D12G2446          ------------------------------------------------------------
Gh_A12G2312          ------------------------------------------------------------
Gh_D08G1069          ------------------------------------------------------------
Gh_A08G2455          ------------------------------------------------------------
Gh_D07G2319          ------------------------------------------------------------
Gh_A07G2108          ------------------------------------------------------------
Gh_A05G2549          ---------------------------------EIQEDWTS----KLGVNLRYCVKVRKN
Gh_D05G2825          ---------------------------------EIQEDWTS----KLGVNLRYCVKVRKN
Gh_A07G2281          ---------------------------------PGNGDWAV----KLGINLFYSANLSRS
Gh_D07G0545          ---------------------------------PGNGDWAV----KLGINLFYSANLSRS
Gh_D09G2376          ---------------------------------PGNGDWAV----KLGINLFYSSNLSHS
Gh_A09G2168          ---------------------------------PGNGDWAV----KLGINLFYSSNLSHS

Gh_A06G0249          ------------------------------------------DIM-FFPKKKG-------
Gh_D02G2008          ------------------------------------------TIINWFKKQQD-------
Gh_A03G1559          ------------------------------------------TIINWFKKQQD-------
Gh_A01G1676          ------------------------------------------------------------
```

```
Gh_D01G1926          ------------------------------------------------------------
Gh_A09G1153          ------------------------------------------------------------
Gh_D09G1159          ------------------------------------------------------------
Gh_D11G2046          ------------------------------------------------------------
Gh_A11G1928          ------------------------------------------------------------
Gh_A06G0175          ------------------------------------------------------------
Gh_D06G0169          ------------------------------------------------------------
Gh_D12G1804          -GLG----------LDIVKEAPLMGNL------ESPYGQVTAFYNYWLGFSTV-------
Gh_Sca005786G01      -GLG----------LDIVKEAPLLGNL------ESPYGQVTAFYNYWLGFSTV-------
Gh_A03G0820          --VD----------KKLLEELEAMG---------FPTARATRALHFSGNTSLE-------
Gh_D02G2408          --VD----------KKLLEELEAMG---------FPTARATRALHFSGNTSLE-------
Gh_D07G1883          ------------------------------------------------------------
Gh_A07G1674          ------------------------------------------------------------
Gh_D03G0179          ESV--ELLKEELYDAILWNEVKSLGDASVQPTLGSEWKTWKHEVMKWFSTSHP-------
Gh_A02G1540          ESV--ELLKEELYDAILWNEVKSLGDASVQPTLGSEWKTWKHEVMKWFSTSHP-------
Gh_D03G0180          ESI--ELLKEELYNAILWNEVKSLGDAPVQPTIGSEWKTWKHEVLKWFSTSHP-------
Gh_A02G1539          ESI--ELLKEELYNAILWNEVKSLGDSPVQSTIGSEWKTWKHEIMKWFSTSHP-------
Gh_D13G1910          TKLNSSNVGFRLLQK----------------------MGWKGKGL---------------
Gh_A13G2297          TKLNSSNVGFRLLQK----------------------MGWKGKGL---------------
Gh_A09G0878          LVFEQ---GFEL--S----------------------HNWKNGSVYFLD-----------
Gh_D09G0901          LVFEQ---GFEL--S----------------------HNWKNGSVYFLGYKAF-------
Gh_D05G0849          LVFDE---EFEL--S----------------------HNWKNGSVYFLGMSSE-------
Gh_A05G0702          LVFDE---EFEL--S----------------------HNWKNGSVYFLGMSSE-------
Gh_D01G2032          LVLHD---KFEL--S----------------------HTGKNGSVYVTGYYVD-------
Gh_D11G3254          LALHS---KFEL--S----------------------HTWKNGSVYFTGYYVD-------
Gh_A11G2870          LALHS---KFEL--S----------------------HTWKNGSVYFTGYYVD-------
Gh_D05G1175          ITTHI--LTHH---P--EKIDNGGKEEE---------KVS--------------------
Gh_A05G3867          ITTHI--LTHH---P--EKIDNGGKEEE---------EVS--------------------
Gh_A05G2341          INSHI--LTHH---T--DEVNKRGKEEE---------GAS--------------------
Gh_D05G2606          INSHI--LTHH---T--DEVNKGGKEEE---------GAS--------------------
Gh_A12G0454          ------------------------------------------------------------
Gh_D12G0457          ------------------------------------------------------------
Gh_A03G1535          VDASI--FGDVL--PIGWKLLLGLKRKE---------GRASV------------------
Gh_D01G1781          VA--T--GGKCM--VLKWVNENTLKA----------------------------------
Gh_A01G1532          VA--T--GGKCM--ILKWVNENTLKA----------------------------------
Gh_A10G0992          --------------------MDASRN----------------------------------
Gh_D10G2570          --------------------MDASRN----------------------------------
Gh_A06G0337          ------------------------------------------------------------
Gh_D06G0368          ------------------------------------------------------------
Gh_D05G3801          ------------------------------------------------------------
Gh_A05G1995          ------------------------------------------------------------
Gh_D07G0542          ------------------------------------------------------------
Gh_A07G0477          ------------------------------------------------------------
Gh_A05G2985          ------------------------------------------------------------
Gh_D04G0776          ------------------------------------------------------------
Gh_D05G0663          VPY---------------------------------------------------------
Gh_A05G3915          VPY---------------------------------------------------------
Gh_D07G0016          VPY---------------------------------------------------------
Gh_A07G0010          VPY---------------------------------------------------------
Gh_D12G2446          ------------------------------------------------------------
Gh_A12G2312          ------------------------------------------------------------
Gh_D08G1069          ------------------------------------------------------------
Gh_A08G2455          ------------------------------------------------------------
Gh_D07G2319          ------------------------------------------------------------
Gh_A07G2108          ------------------------------------------------------------
Gh_A05G2549          SSFKQ--VQHAL--PLSGLFTDKY-------------SSLELFNIKWKSRKSRSKGKLNH
Gh_D05G2825          SSFKQ--VQHAL--PLSGLFTDKY-------------SSLELFNIKWQSRKSRSKGKLNH
Gh_A07G2281          TLYSK--Q---M--PYNWIIYSAFGRNS---------SDSSPKKLNVYGRRS---GKPRK
Gh_D07G0545          TLYSK--Q---M--PYNWIIYSAFGRNS---------SDSSPKKLNVYGRRS---GKPRK
Gh_D09G2376          TLYSK--Q---M--PYNFVIYSAFGRNS---------PASSPTKLNSYGRRS---RKQKK
Gh_A09G2168          MLYSK--Q---M--PYNSVIYSAFGRNS---------PTSSLTKLNSYGRRS---RKQKK
```

```
Gh_A06G0249         -FV--L------HE--------AN-----------------GTVG---------------
Gh_D02G2008         -LF--LKDNFLVEV--------AD-----------------DCVKAHGPTEV---Q---E
Gh_A03G1559         -LF--LKDNFLVEV--------AD-----------------DCVKAHGPTEV---Q---E
Gh_A01G1676         ------------------------------------------------------------
Gh_D01G1926         ------------------------------------------------------------
Gh_A09G1153         ------------------------------------------------------------
Gh_D09G1159         ------------------------------------------------------------
Gh_D11G2046         ------------------------------------------------------------
Gh_A11G1928         ------------------------------------------------------------
Gh_A06G0175         ------------------------------------------------------------
Gh_D06G0169         ------------------------------------------------------------
Gh_D12G1804         -MD--F---VWVD---------EYD--------------AMAGPNRK----SRRVM----
Gh_Sca005786G01     -MD--F---VWVD---------EYD--------------AMAGPNRK----SRRVM----
Gh_A03G0820         -AA--V---NWVV---------EHE--------------ADPDIDQMPMVPVNRHVEASK
Gh_D02G2408         -AA--V---NWVV---------EHE--------------ADSDIDQMPMVPVNRHVEASK
Gh_D07G1883         ----------------------------------------------------RKAKEEAK
Gh_A07G1674         ----------------------------------------------------RKAKEEAK
Gh_D03G0179         -VS--SA---------------GD-------------------VNQRSSDGL--------
Gh_A02G1540         -VS--ST---------------GD-------------------VNQRSSDGL--------
Gh_D03G0180         -VS--TA---------------GD-------------------VNQRNSGSP--------
Gh_A02G1539         -VS--TA---------------GD-------------------VNQRNSGSP--------
Gh_D13G1910         ----------------------GKD--------------EQAGIIEPIRSGIRDPKLGIG
Gh_A13G2297         ----------------------GKD--------------EQ-GIIEPIRSGIRDPKLGIG
Gh_A09G0878         -------------S--------EEE--------------SSEDEELPVA------AE-NG
Gh_D09G0901         -VP--EEGYPFINS--------EEE--------------SSEDEELPVA------AE-NG
Gh_D05G0849         -E--------------------ESE--------------EEEDEELPVAAAAAAAAV-NG
Gh_A05G0702         -E--------------------ESE--------------EEEEEELPVAAA--AAAV-NG
Gh_D01G2032         -TS--QGS--------------DTE--------------SEEDLLEPTMNLV--------
Gh_D11G3254         -TP--QGS--------------GSE--------------SEEELLEPIVNPI--------
Gh_A11G2870         -TP--QGS--------------GSE--------------SEEELLEPIVNPV--------
Gh_D05G1175         ------------------------------------------------------------
Gh_A05G3867         ------------------------------------------------------------
Gh_A05G2341         ------------------------------------------------------------
Gh_D05G2606         ------------------------------------------------------------
Gh_A12G0454         ------------------------------------------------------------
Gh_D12G0457         ------------------------------------------------------------
Gh_A03G1535         -----YCRR---------------------------------------------------
Gh_D01G1781         ------------------------------------------------------------
Gh_A01G1532         ------------------------------------------------------------
Gh_A10G0992         ------------------------------------------------------------
Gh_D10G2570         ------------------------------------------------------------
Gh_A06G0337         ------------------------------------------------------------
Gh_D06G0368         ------------------------------------------------------------
Gh_D05G3801         ------------------------------------------------------------
Gh_A05G1995         ------------------------------------------------------------
Gh_D07G0542         ------------------------------------------------------------
Gh_A07G0477         ------------------------------------------------------------
Gh_A05G2985         ------------------------------------------------------------
Gh_D04G0776         ------------------------------------------------------------
Gh_D05G0663         ------------------------------------------------------------
Gh_A05G3915         ------------------------------------------------------------
Gh_D07G0016         ------------------------------------------------------------
Gh_A07G0010         ------------------------------------------------------------
Gh_D12G2446         --------------------------------------------QARPSIFP--------
Gh_A12G2312         --------------------------------------------QARPSIFP--------
Gh_D08G1069         --------------------------------------------QARPSRFP--------
Gh_A08G2455         --------------------------------------------QARPSRFP--------
Gh_D07G2319         ------------------------------------------------------------
Gh_A07G2108         ------------------------------------------------------------
Gh_A05G2549         PSPSKPCESVEMKVDE--IM--------------------VEKLD---SDIS-KY-----
Gh_D05G2825         PSPSKPCESVEMKVDE--IM--------------------MEKLD---SDIS-KY-----
Gh_A07G2281         VVAGKWCGKVWMSNQVHPFLAQRDPEEQEQERSFHARAISDENVERKPENVL-KA-----
Gh_D07G0545         VVVGKWCGKVWMSNQVHPFLAQRDSEEQEQERSFHAQAISDENVERKPENVP-KA-----
```

```
Gh_D09G2376          VVAGKWCGKVWMSNQVHPFLTQRDPKEQEQEKSFHAWATSDENLESKPENIR-KA-----
Gh_A09G2168          VVAGKWCGKVWMSNQVHPFLTQRDPGEQEQEKSYHAWATSDENLESKPENIR-KA-----


Gh_A06G0249          --------DADTSTLTNGPKSEKLTTDQ--------------------------------
Gh_D02G2008          KLIANE--GTETSILQKGIKPVQIAEKS--------------------------------
Gh_A03G1559          KLIANEPKGTETSILQKGIKPVQITEKW--------------------------------
Gh_A01G1676          ------------------------------------------------------------
Gh_D01G1926          ------------------------------------------------------------
Gh_A09G1153          ------------------------------------------------------------
Gh_D09G1159          ------------------------------------------------------------
Gh_D11G2046          ------------------------------------------------------------
Gh_A11G1928          ------------------------------------------------------------
Gh_A06G0175          ------------------------------------------------------------
Gh_D06G0169          ------------------------------------------------------------
Gh_D12G1804          ---EEEN-----KKLRKKAKREYNETVR------GLA------------EFVK-------
Gh_Sca005786G01      ---EEEN-----KKLRKKAKREYNETVR------GLA------------EFVK-------
Gh_A03G0820          PSLTAEEMKLKAQELRERARKKKEEEER------RMEREREKERIRVGKELLEAK-----
Gh_D02G2408          PSLTPEEMKLKAQELRERARKKKEEEER------RMEREREKERIRVGKELLEAK-----
Gh_D07G1883          KQR--D----AIEAAQRSRRLDAIEAQL--KAEQQMGESLLAGRGVVFYKILEAV-----
Gh_A07G1674          KQR--D----AIEAAQRSRRLDAIEAQL--KAEQQMGESLLAGRGVVFYKILEAV-----
Gh_D03G0179          -------SNTNIQVSRKRAKLEVRRADTHASMVQSNG-SDQT----MAVE-IDSD-----
Gh_A02G1540          -------SNTNIQVSRKRAKLEVRRADTHVSMVQSNG-SDQT----MAVE-IDSD-----
Gh_D03G0180          -------SNTNIQVSRKRPKLEVRRADTHAFQVQSNG-SEQT----MAAE-IESD-----
Gh_A02G1539          -------SNTNIQVSGKRPKLEVRRADTDASQVQSNG-SDQT----MAAE-IDSD-----
Gh_D13G1910          KQEEDDFFTAEENIQRRKLDIEVEETEEHAKKREVLAEREQK--IQTEVKEIRKV-----
Gh_A13G2297          KQEEDDFFTAEENIQRRKLDIEVEETEEHAKKREVLAEREQK--IQTEVKEIRKV-----
Gh_A09G0878          KAK-TDAKTAKAN-----------------------AGKPDA--VKQAVKITEPS-----
Gh_D09G0901          KAK-TDAKTAKAN-----------------------AGKPDA--VKQAVKIAEPS-----
Gh_D05G0849          KAK-PDAKTAKAN-----------------------AGKPDA--VKQSGKIAEPS-----
Gh_A05G0702          KAK-PDGKTAKAN-----------------------AGKPDA--VKQSGKIAEPS-----
Gh_D01G2032          --K-S-----EPP-----------------------ASDPTT---TKQVKIVEPK-----
Gh_D11G3254          --K-P-----HAT-----------------------ISDPTT---SKQVKIVEPK-----
Gh_A11G2870          --K-P-----HST-----------------------ASDPTT---SKQVKIVEPK-----
Gh_D05G1175          ----------------------------------------------TSNECQE-------
Gh_A05G3867          ----------------------------------------------MSNECQE-------
Gh_A05G2341          ----------------------------------------------SSNTCEE-------
Gh_D05G2606          ----------------------------------------------SSNTCEE-------
Gh_A12G0454          ------------------------------------------------------------
Gh_D12G0457          ------------------------------------------------------------
Gh_A03G1535          ----------------------YISP-----------------GGRHFISCKE-------
Gh_D01G1781          ------------------------------------------------------------
Gh_A01G1532          ------------------------------------------------------------
Gh_A10G0992          ------------------------------------------------------------
Gh_D10G2570          ------------------------------------------------------------
Gh_A06G0337          ------------------------------------------------------------
Gh_D06G0368          ------------------------------------------------------------
Gh_D05G3801          ------------------------------------------------------------
Gh_A05G1995          ------------------------------------------------------------
Gh_D07G0542          ------------------------------------------------------------
Gh_A07G0477          ------------------------------------------------------------
Gh_A05G2985          ------------------------------------------------------------
Gh_D04G0776          ------------------------------------------------------------
Gh_D05G0663          ------------------------------------------------------------
Gh_A05G3915          ------------------------------------------------------------
Gh_D07G0016          ------------------------------------------------------------
Gh_A07G0010          ------------------------------------------------------------
Gh_D12G2446          -----------------------ISS-------------------QELFSI--LDDASER
Gh_A12G2312          -----------------------ISS-------------------QELFSI--LDDASER
Gh_D08G1069          -----------------------ISS-------------------QELFSI--LDDASEG
Gh_A08G2455          -----------------------ISS-------------------QELFSI--LDDASEG
Gh_D07G2319          ------------------------------------------------------------
Gh_A07G2108          ------------------------------------------------------------
```

```
Gh_A05G2549          -----G-QK-IIQYSRRKKRKSDYST-----------------GGGGGVELLKN-DLPRE
Gh_D05G2825          -----G-QK-IIQYTRRKKRKSDYST-----------------GGGGGVELLKN-DLPRE
Gh_A07G2281          -----ETMKVVN---RKRKSRAEITL-------------------NKKVKRVESEGAVLD
Gh_D07G0545          -----ETMKVVN---RKRKSRAEITL-------------------NKKVKRVESEGAVSD
Gh_D09G2376          -----ETSKVVKMFTRKSKTRAGATP-------------------SKKAKCIEPESVVSD
Gh_A09G2168          -----ETSKVAKRFTRKSKTRAGTTP-------------------SKKAKCIEPESVVSD


Gh_A06G0249          ------------------------------------------------------------
Gh_D02G2008          ---------------------------------------------------EVAPIRKTD
Gh_A03G1559          ---------------------------------------------------EVAPIPKTD
Gh_A01G1676          ------------------------------------------------------------
Gh_D01G1926          ------------------------------------------------------------
Gh_A09G1153          ------------------------------------------------------------
Gh_D09G1159          ------------------------------------------------------------
Gh_D11G2046          ------------------------------------------------------------
Gh_A11G1928          ------------------------------------------------------------
Gh_A06G0175          ------------------------------------------------------------
Gh_D06G0169          ------------------------------------------------------------
Gh_D12G1804          ------------------KRDKRV-----ID--M-----SVKRK------EEMERKKEEE
Gh_Sca005786G01      ------------------KRDKRV-----ID--M-----SVKRK------EEMERKKEEE
Gh_A03G0820          -------------RIEEENERKRL-----VA--L-----RKAEK------EEEKRARE--
Gh_D02G2408          -------------RIEEENERKRL-----VA--L-----RKAEK------EEEKRARE--
Gh_D07G1883          -------------PFQGSGDK---------------------------------------
Gh_A07G1674          -------------PFQGSGDK---------------------------------------
Gh_D03G0179          -------------FFSN----RDA-----VD-----------------------------
Gh_A02G1540          -------------FFSN----RDA-----VD-----------------------------
Gh_D03G0180          -------------FFSN----RDA-----VD-----------------------------
Gh_A02G1539          -------------FLSN----RDA-----VD-----------------------------
Gh_D13G1910          -------------FYCELCNKQYK-----LA--M-----E--------------------
Gh_A13G2297          -------------FYCELCNKQYK-----LA--M-----E--------------------
Gh_A09G0878          -------------DNKKNEDDDDD-----SD-----------------------------
Gh_D09G0901          -------------NNKKNEDDDDD-----SD-----------------------------
Gh_D05G0849          -------------NNRKSEDEDDD-----DE-----------------------------
Gh_A05G0702          -------------SNKKSEDEDDD-----DE-----------------------------
Gh_D01G2032          -------------KDDDSSD-EDE-----ED--S-----S--------------------
Gh_D11G3254          -------------KAEDSCDDEDE-----DD--T-----S--------------------
Gh_A11G2870          -------------KAEDSSDDEDE-----DD--T-----S--------------------
Gh_D05G1175          ------------------------------------------------------------
Gh_A05G3867          ------------------------------------------------------------
Gh_A05G2341          ------------------------------------------------------------
Gh_D05G2606          ------------------------------------------------------------
Gh_A12G0454          ------------------------------------------------------------
Gh_D12G0457          ------------------------------------------------------------
Gh_A03G1535          ------------------------------------------------------------
Gh_D01G1781          ------------------------------------------------------------
Gh_A01G1532          ------------------------------------------------------------
Gh_A10G0992          ------------------------------------------------------------
Gh_D10G2570          ------------------------------------------------------------
Gh_A06G0337          ------------------------------------------------------------
Gh_D06G0368          ------------------------------------------------------------
Gh_D05G3801          ------------------------------------------------------------
Gh_A05G1995          ------------------------------------------------------------
Gh_D07G0542          ------------------------------------------------------------
Gh_A07G0477          ------------------------------------------------------------
Gh_A05G2985          ------------------------------------------------------------
Gh_D04G0776          ------------------------------------------------------------
Gh_D05G0663          ------------------------------------------------------------
Gh_A05G3915          ------------------------------------------------------------
Gh_D07G0016          ------------------------------------------------------------
Gh_A07G0010          ------------------------------------------------------------
Gh_D12G2446          TFLCGAAVSMQKYIFDGEAGKIGLETKNLVACTSFLVEQKLV--------KAW-LADKDA
Gh_A12G2312          TFLCGAAVSMQKYIFDGEAGKIGLETKNLVACTSFLVEQKLV--------KAW-LADKDA
```

```
Gh_D08G1069          TFLCGAAVSMQKYIFDGEAGKIGLDTKNLVACTSFLVEQKLV--------KAW-LADKDA
Gh_A08G2455          TFLCGAAVSMQKYIFDGEAGKIGLDTKNLVACTSFLVEQKLV--------KAW-LAAKDA
Gh_D07G2319          ------------------------------------------------------------
Gh_A07G2108          ------------------------------------------------------------
Gh_A05G2549          DSAASSQL---LDKHGGNKSKINSRSES-IQAQLEVPTTSVVQRDQNKIVEESGPDDEAQ
Gh_D05G2825          DSAASSQF---LDKHGGNKSKINARSES-IQAQLEVPTTSVVQRDQNRIVEETGPDDEAQ
Gh_A07G2281          DSLDGSSLRQQQIVFRGKKPRLVEKEET-VSCDLL----------------------EDD
Gh_D07G0545          DSLDGSSLRQQQIVFRGKKPRLVEKEEA-VSCDLL----------------------EDD
Gh_D09G2376          DSLDGNSLRQQQRFFRGKKPKLIEKEKE-ISYDSL----------------------EDD
Gh_A09G2168          DSLDGNSLRQQQRFFRGKKPKLIEKEKE-ISYDSL----------------------DDD


Gh_A06G0249          ---GN-----------------------V-----------NISYGNTEVISTQKRVQKEW
Gh_D02G2008          ---PNVIQTVNNTT------VTLS--ETM-----------GEVGPDLPKISSDKQVQKEW
Gh_A03G1559          ---PNVIQTVNNTT------ATLP--ETV-----------GEVGPDLPEIPSDKQVQKEW
Gh_A01G1676          ------------------------------------------------------------
Gh_D01G1926          ------------------------------------------------------------
Gh_A09G1153          ------------------------------------------------------------
Gh_D09G1159          ------------------------------------------------------------
Gh_D11G2046          ------------------------------------------------------------
Gh_A11G1928          ------------------------------------------------------------
Gh_A06G0175          ------------------------------------------------------------
Gh_D06G0169          ------------------------------------------------------------
Gh_D12G1804          ---RERKRKLEKERLARVKAY----EEPE------WAKVEEEEVDNWDEMDEKEREKEEF
Gh_Sca005786G01      ---RERKRKLEKERLARVKAY----EEPE------WAKVEEEEVDNWDEMDEKEREKEEF
Gh_A03G0820          ----KIRQKLEEDKAERRRKLGLPPEEPSAAPKPSASIVEEKK-SALP-VRPATKAEH--
Gh_D02G2408          ----KIRQKLEEDKAERRRKLGLPPEEPSAAPKPSAPIVEEKK-SALP-VRPATKAEQ--
Gh_D07G1883          --------------------IKLPP---------S--CFTELSDQGAFDKGPM-------
Gh_A07G1674          --------------------IKLPP---------S--CFTELSDQGAFDKGPM-------
Gh_D03G0179          --------------------VNLP----T----PQH-CKKEDERE---ETTPM-------
Gh_A02G1540          --------------------VNLP----T----PQH-CKKEDERE---ETTPM-------
Gh_D03G0180          --------------------VNLP----T----PRL-CRKEEERE---ETTPM-------
Gh_A02G1539          --------------------VNLP----I----PRL-CRKEDERE---ETTPM-------
Gh_D13G1910          ------------------FEVHLSSY-------------DHNHRKRFKEMREM-------
Gh_A13G2297          ------------------FEVHLSSY-------------DHNHRKRFKEMREM-------
Gh_A09G0878          ------------------SEDGSG-------------SDDED--------EEM-------
Gh_D09G0901          ------------------SEDGSG-------------SDDEDDSE--DEDEEM-------
Gh_D05G0849          ------------------SEDESG-------------SDEEDDSDDDDESDEM-------
Gh_A05G0702          ------------------SEDESG-------------SDEEDDSDDDDESDEM-------
Gh_D01G2032          ------------------AEDEESSEEQE----PRMLVNGENDSDD-ADSDED-------
Gh_D11G3254          ------------------TEDEMSSEDQE----PGMLVNGENESDNDTDSDED-------
Gh_A11G2870          ------------------TEDEMSSEDQE----PGMLVNGENESNNDTDSDED-------
Gh_D05G1175          ------------------------------------------------------------
Gh_A05G3867          ------------------------------------------------------------
Gh_A05G2341          ------------------------------------------------------------
Gh_D05G2606          ------------------------------------------------------------
Gh_A12G0454          ------------------------------------------------------------
Gh_D12G0457          ------------------------------------------------------------
Gh_A03G1535          ------------------------------------------------------------
Gh_D01G1781          ------------------------------------------------------------
Gh_A01G1532          ------------------------------------------------------------
Gh_A10G0992          ------------------------------------------------------------
Gh_D10G2570          ------------------------------------------------------------
Gh_A06G0337          ------------------------------------------------------------
Gh_D06G0368          ------------------------------------------------------------
Gh_D05G3801          ------------------------------------------------------------
Gh_A05G1995          ------------------------------------------------------------
Gh_D07G0542          ------------------------------------------------------------
Gh_A07G0477          ------------------------------------------------------------
Gh_A05G2985          ------------------------------------------------------------
Gh_D04G0776          ------------------------------------------------------------
Gh_D05G0663          ------------------------------------------------------------
Gh_A05G3915          ------------------------------------------------------------
```

```
Gh_D07G0016          ------------------------------------------------------------
Gh_A07G0010          ------------------------------------------------------------
Gh_D12G2446          EALRCQKLLVEEEEAAQKRQVELL----------------ERKRQKKLRQKEQ-------
Gh_A12G2312          EALRCQKLLVEEEEAAQKRQVELL----------------ERKRQKKLRQKEQ-------
Gh_D08G1069          EALRCQKLLVEEEEAAHKRQVELL----------------ERKRQKKLRQKEQ-------
Gh_A08G2455          EALRCQKLLVEEEEAAHKRQVELL----------------ERKRQKKLRQKEQ-------
Gh_D07G2319          ------------------------------------------------------------
Gh_A07G2108          ------------------------------------------------------------
Gh_A05G2549          SLIACASSI-------KKCENKLM----------------ERNSENDESSP---------
Gh_D05G2825          SLIACAGSI-------KKCENKLM----------------ERNSENGEISP---------
Gh_A07G2281          SLLRHWNLS-------RNRRAKFI----------------ERENAESEDAEED-------
Gh_D07G0545          SLLRHWNLS-------RNRRAKFI----------------ERENAESEDAEED-------
Gh_D09G2376          SLLHHRDLS-------RRKGAKFI----------------EREASESEDVEED-------
Gh_A09G2168          SLLHHRDLS-------RRKGAKFI----------------EREASESEDVEED-------


Gh_A06G0249          SCVLCLISTSSEYCLKEHLRGKKHKTKEYELRVGALP----LKETCML-SSMPK------
Gh_D02G2008          TCAMCQVTTTSEKTLKSHLRGRKHKNKLMQANN--QP----CKGKAGL-ASKVK------
Gh_A03G1559          TCAMCQVTTTSEKTLNSHLRGRKHKDNLMQANN--QP----CKGKAGL-ASKVK------
Gh_A01G1676          ------------------------------------------------------------
Gh_D01G1926          ------------------------------------------------------------
Gh_A09G1153          ------------------------------------------------------------
Gh_D09G1159          ------------------------------------------------------------
Gh_D11G2046          ------------------------------------------------------------
Gh_A11G1928          ------------------------------------------------------------
Gh_A06G0175          ------------------------------------------------------------
Gh_D06G0169          ------------------------------------------------------------
Gh_D12G1804          YCVACGKKFKSEKQWKNHEQSKKHKEKVAELR--------------EL-FIE--------
Gh_Sca005786G01      YCVACGKKFKSEKQWKNHEQSKKHKEKVAELR--------------ES-FIE--------
Gh_A03G0820          -MRECLRSLKQ-----------NHKDDDAKVK--------------RA-FQT--------
Gh_D02G2408          -MRECLRSLKQ-----------NHKDDDAKVK--------------RA-FQT--------
Gh_D07G1883          YF----------------------------------------------------------
Gh_A07G1674          YF----------------------------------------------------------
Gh_D03G0179          D-----TSNSLTDRWNNIVVEARHPEVIHTKNVEITTASEEVKTTSTL-HIQPK------
Gh_A02G1540          D-----TSNSLTDRWNNIVVEARHPEVIHTKNVEITTASEEVKSTSTL-HIQPK------
Gh_D03G0180          D-----RSNNLTDRWDNIVVEARHSEVIHTKNVEITTASEEVNSTSTL-HIQSK------
Gh_A02G1539          D-----RSNNLTDRWDNIVVEARHSEVIHTKNVEITTASEEVNSTSTL-HIQSK------
Gh_D13G1910          H-----GSSSRDDRQKREQ--QRQ-EREMAKFAQMAGARKQQQQESRE-ESGPA------
Gh_A13G2297          H-----GSSSRDDRQKREQ--QRQ-EREMAKFAQMAGARKQQQQESRE-ESGPA------
Gh_A09G0878          SV---DESSDDEDEETPKK--------VES--------SKK----------RPA------
Gh_D09G0901          SV---DESS-DEDEETPKK--------VES--------SKK----------RPA------
Gh_D05G0849          SM---DESSDDEDEETPKK--------VESSKKIPVESSKK----------RPA------
Gh_A05G0702          SM---DESSDDEDEETPKK--------VES--------SKK----------RPA------
Gh_D01G2032          DS---EEDSSDEDQKTP----------EK------AGPSKK----------RPA------
Gh_D11G3254          DS---EEESSDEDPETPET--------EK------AGPSKK----------RSA------
Gh_A11G2870          DS---EEESSDEDPETPET--------EK------AGPSKK----------RSA------
Gh_D05G1175          ------------------------------------------------------------
Gh_A05G3867          ------------------------------------------------------------
Gh_A05G2341          ------------------------------------------------------------
Gh_D05G2606          ------------------------------------------------------------
Gh_A12G0454          ------------------------------------------------------------
Gh_D12G0457          ------------------------------------------------------------
Gh_A03G1535          ------------------------------------------------------------
Gh_D01G1781          ------------------------------------------------------------
Gh_A01G1532          ------------------------------------------------------------
Gh_A10G0992          ------------------------------------------------------------
Gh_D10G2570          ------------------------------------------------------------
Gh_A06G0337          ------------------------------------------------------------
Gh_D06G0368          ------------------------------------------------------------
Gh_D05G3801          ------------------------------------------------------------
Gh_A05G1995          ------------------------------------------------------------
Gh_D07G0542          ------------------------------------------------------------
Gh_A07G0477          ------------------------------------------------------------
```

```
Gh_A05G2985        ------------------------------------------------------------
Gh_D04G0776        ------------------------------------------------------------
Gh_D05G0663        ------------------------------------------------------------
Gh_A05G3915        ------------------------------------------------------------
Gh_D07G0016        ------------------------------------------------------------
Gh_A07G0010        ------------------------------------------------------------
Gh_D12G2446        ---------KAKEQRH--------WELEESKPN--MD--DLLEVNTLAETSSPSA-VDS-
Gh_A12G2312        ---------KAKEQRH--------WELEESKPN--MD--DLLEVNTLAETSSPSA-VDS-
Gh_D08G1069        ---------KAKELRP--------WEMEEGKQN--MD--DSLEDNTPAETSSPKAVFDI-
Gh_A08G2455        ---------KAKELRP--------WEMEEGKQN--MD--DSLEDSTPAVTSSPKAVFDI-
Gh_D07G2319        ------------------------------------------------------------
Gh_A07G2108        ------------------------------------------------------------
Gh_A05G2549        ----------AEKCSKFCLVADDEVYLENT-AT--AT--KVCNPVSEQQSDEPTSGYGLI
Gh_D05G2825        ----------AEKCSKFCLVADGEVYLENT-AT--AT--KVCNPVSEQQSDEPTSGYGLI
Gh_A07G2281        ---------FTHQQQRSNLRGRHNKYIEEDDEV--SG--DLLNESSLK-------QYR--
Gh_D07G0545        ---------FTHQQQRSNLRGRHHKYIEEDDEI--SG--DLLNESSLK-------QYR--
Gh_D09G2376        ---------SDDQQFLKNLGGKKGKYIVENDVV--SG--DSLDKISTK-------QYR--
Gh_A09G2168        ---------SDDQQFRKNLGGKQGKYIVENDVV--SG--DSLDKTSAK-------QYR--

Gh_A06G0249        -----KVEKVV--------LFRN-------------------------------------
Gh_D02G2008        -----QSEVSRREPQKQSEVSRK------------------------------E---PQK
Gh_A03G1559        -----QSEVS-----------RK------------------------------E---PQK
Gh_A01G1676        ------------------------------------------------------------
Gh_D01G1926        ------------------------------------------------------------
Gh_A09G1153        ------------------------------------------------------------
Gh_D09G1159        ------------------------------------------------------------
Gh_D11G2046        ------------------------------------------------------------
Gh_A11G1928        ------------------------------------------------------------
Gh_A06G0175        ------------------------------------------------------------
Gh_D06G0169        ------------------------------------------------------------
Gh_D12G1804        ------------------------------------------------------------
Gh_Sca005786G01    ------------------------------------------------------------
Gh_A03G0820        ------------------------------------------------------------
Gh_D02G2408        ------------------------------------------------------------
Gh_D07G1883        ------------------------QLSL--------VHQ-----------EGSS---ATK
Gh_A07G1674        ------------------------QLSL--------VHQ-----------EGSS---ATK
Gh_D03G0179        -----EVE---LTPVNETVAKKSID-------------------------SG--------
Gh_A02G1540        -----EVE---LTPVNEAVAKKSID-------------------------AG--------
Gh_D03G0180        -----EVK---LTPVNEAVAKKSID-------------------------AG--------
Gh_A02G1539        -----EVE---LTPVNEAVAKKSMD-------------------------AG--------
Gh_D13G1910        -----TAPASSPAPAPAPAPAPAIATAL--------ADQE----QRKTLKFGFS---SKS
Gh_A13G2297        -----TT--------PAPAPASAIATAL--------ADQE----QRKTLKFGFS---SKS
Gh_A09G0878        -----EAAT----PVSAKKAKPA----A--------TPQK----TDG-KKGGHT---ATP
Gh_D09G0901        -----EAAT----PVSAKKAKPA----A--------TPQK----TDG-KKGGHT---ATP
Gh_D05G0849        -----EAAT----PASAKKAKSA----V--------TPQK----TDG-KKGGHT---ATP
Gh_A05G0702        -----EAAT----PASAKKAKSA----V--------TPQK----TDG-KKGGHT---ATP
Gh_D01G2032        -----ESSKKT--PAPEKKA-KL-------------------------KAGGHT---ATP
Gh_D11G3254        -----ESATKT--PAPEKKA-KL----V--------TPQK----TDGKKVGGHT---ATP
Gh_A11G2870        -----ESATKT--PAPEKKA-KL----V--------TPQK----TDGKKVGGHT---ATP
Gh_D05G1175        ------------------------------------------------------------
Gh_A05G3867        ------------------------------------------------------------
Gh_A05G2341        ------------------------------------------------------------
Gh_D05G2606        ------------------------------------------------------------
Gh_A12G0454        ------------------------------------------------------------
Gh_D12G0457        ------------------------------------------------------------
Gh_A03G1535        ------------------------------------------------------------
Gh_D01G1781        ------------------------------------------------------------
Gh_A01G1532        ------------------------------------------------------------
Gh_A10G0992        ------------------------------------------------------------
Gh_D10G2570        ------------------------------------------------------------
Gh_A06G0337        ------------------------------------------------------------
Gh_D06G0368        ------------------------------------------------------------
```

```
Gh_D05G3801          ------------------------------------------------------------
Gh_A05G1995          ------------------------------------------------------------
Gh_D07G0542          ------------------------------------------------------------
Gh_A07G0477          ------------------------------------------------------------
Gh_A05G2985          ------------------------------------------------------------
Gh_D04G0776          ------------------------------------------------------------
Gh_D05G0663          ------------------------------------------------------------
Gh_A05G3915          ------------------------------------------------------------
Gh_D07G0016          ------------------------------------------------------------
Gh_A07G0010          ------------------------------------------------------------
Gh_D12G2446          DGQNPLMSTDQIIPSVEPNLFSKLEENVDYEIQMGFSNGYSD--PGIS-QN-IERRT---
Gh_A12G2312          DGQNPVMSTDQIIPSVEPNLFSKLLENVDYEIQTGFSNGYSD--PGIS-QN-IERRT---
Gh_D08G1069          DGQNAIISTDQVLPALEPIPFPNPEEGVDYRVQTEFYNQYCE--PGTS-QN-GERRV---
Gh_A08G2455          DGQNAVISTDQVLPALEPIPFPNPEEGVDYRVQTEFYNRYCE--PGTS-QN-GEKRV---
Gh_D07G2319          ------------------------------------------------------------
Gh_A07G2108          ------------------------------------------------------------
Gh_A05G2549          NGNSA----SSHSAQRCAGRYNQGLEDITVPK-------F--SINGGA-FSG--------
Gh_D05G2825          NGNSA----SSHSAQRCAGRYNQGLEDITVPK-------F--SINGGA-FSG--------
Gh_A07G2281          -----------RTPRSLQAKFSDGENGVSDDEQEEISHKLHRRIPRGKQIKSSKRNTAVS
Gh_D07G0545          -----------RTPRSLQAKFLDGENGVSDDEQEEISHKLHRRIPRGKQIKSSKRNTAVS
Gh_D09G2376          -----------RIPRSSRDKFMKREGSVSADEQEEISYQFHKRIPRGRQIKLFERRLAVS
Gh_A09G2168          -----------RIPRSCLAKFMKREGSVSADEQEEISHQFHKRIPRGRHIKLFERRLAVS


Gh_A06G0249          -----LN------------IETWSG-----------LLHP------------------VT
Gh_D02G2008          KGS--KN------------YEPPKGQMSASSAACSNAVNP------------------KT
Gh_A03G1559          KGS--KK------------QEPPKGQISASSAACSNAVNP------------------KT
Gh_A01G1676          ------------------------------------------------------------
Gh_D01G1926          ------------------------------------------------------------
Gh_A09G1153          ------------------------------------------------------------
Gh_D09G1159          ------------------------------------------------------------
Gh_D11G2046          ------------------------------------------------------------
Gh_A11G1928          ------------------------------------------------------------
Gh_A06G0175          ------------------------------------------------------------
Gh_D06G0169          ------------------------------------------------------------
Gh_D12G1804          -------------------EEEEEG--------DLEVEAD------------------VE
Gh_Sca005786G01      -------------------EEEEEG--------DLEVEAD------------------LE
Gh_A03G0820          -------------------LLTYIG--------NVARNPD-------------------E
Gh_D02G2408          -------------------LLTYIG--------NVARNPD-------------------E
Gh_D07G1883          DDDKENN------------RTTHSG--------VLEFTAD------------------EG
Gh_A07G1674          DDEKGNN------------RTTHSG--------VLEFTAD------------------EG
Gh_D03G0179          --SKNRQ--------------------------CTAF---------------------IE
Gh_A02G1540          --SKNRQ--------------------------CIAF---------------------IE
Gh_D03G0180          --SKNRQ--------------------------CVAF---------------------ID
Gh_A02G1539          --SKNRQ--------------------------CVAF---------------------IE
Gh_D13G1910          SSSKNAS--------------------------GSAVK----------------------
Gh_A13G2297          SSSKNAS--------------------------GSAVK----------------------
Gh_A09G0878          HPSKQAG--------------------------KTSGK----------------------
Gh_D09G0901          HPSKQAG--------------------------KTSGK----------------------
Gh_D05G0849          HPAKQSG--------------------------KNSAK----------------------
Gh_A05G0702          HPAKQSG--------------------------KNSAK----------------------
Gh_D01G2032          HTSKKAG--------------------------KTSAAAA------------------AA
Gh_D11G3254          HPSKEAR--------------------------KASAT----------------------
Gh_A11G2870          HPSKQAR--------------------------KALAT----------------------
Gh_D05G1175          ------------------------------------------------------------
Gh_A05G3867          ------------------------------------------------------------
Gh_A05G2341          ------------------------------------------------------------
Gh_D05G2606          ------------------------------------------------------------
Gh_A12G0454          ------------------------------------------------------------
Gh_D12G0457          ------------------------------------------------------------
Gh_A03G1535          ------------------------------------------------------------
Gh_D01G1781          ------------------------------------------------------------
Gh_A01G1532          ------------------------------------------------------------
```

```
Gh_A10G0992          ------------------------------------------------------------
Gh_D10G2570          ------------------------------------------------------------
Gh_A06G0337          ------------------------------------------------------------
Gh_D06G0368          ------------------------------------------------------------
Gh_D05G3801          ------------------------------------------------------------
Gh_A05G1995          ------------------------------------------------------------
Gh_D07G0542          ------------------------------------------------------------
Gh_A07G0477          ------------------------------------------------------------
Gh_A05G2985          ------------------------------------------------------------
Gh_D04G0776          ------------------------------------------------------------
Gh_D05G0663          ------------------------------------------------------------
Gh_A05G3915          ------------------------------------------------------------
Gh_D07G0016          ------------------------------------------------------------
Gh_A07G0010          ------------------------------------------------------------
Gh_D12G2446          ------EQV-GYRHTVVARRQTPPNSQRGV---LNDFHASQNSHGYASQNSHGFKCGGIN
Gh_A12G2312          ------EQV-GRRHIVVARRQTPPNSQRGV---LNDFHASQN--------SHGFKCGGIN
Gh_D08G1069          ------EQVDGCQRIVVARWKTPPKSQRVV---LNGFHANQN--------SHGFKSGGTN
Gh_A08G2455          ------EQVDGCQRKVVARWKTPPKSQRVV---LNGFHANQN--------SHGFKSGGTN
Gh_D07G2319          ------------------------------------------------------------
Gh_A07G2108          ------------------------------------------------------------
Gh_A05G2549          --MTSENEVQQGTEA-------TSRNNSEV------IIRSEVLKE---------------
Gh_D05G2825          --MTSENEVQQGTEA-------TSRNNSEV------IIRSEVPKE---------------
Gh_A07G2281          D-----DSLKQYRRM-------RKGKQTKV------FERDDAMSDYASD-D---------
Gh_D07G0545          D-----DSLKQYRRM-------RKGKQTKV------FERDDAMSDYASD-D---------
Gh_D09G2376          DDSRADNSLKQYRRK-------PKGKRAKF------FEREEAMSDDASDND---------
Gh_A09G2168          DDSRADNSLKQYRRM-------PKGKRAKF------FEREEAMSDDASDND---------


Gh_A06G0249          R----SIRWCKWKKP-E--IGCVKL-----NTDGSVD--A-----------GNSGFGG--
Gh_D02G2008          EMSKSNLPKEEWKKV-IPTTNMVGNH---VKSSENVQ--G-----------QQQQVEKEH
Gh_A03G1559          EMSKSNLPQEESKKA-IPTTNMVGNH---VKSSENVQ--G-----------QQQQVGKKH
Gh_A01G1676          ------------------------------------------------------------
Gh_D01G1926          ------------------------------------------------------------
Gh_A09G1153          ------------------------------------------------------------
Gh_D09G1159          ------------------------------------------------------------
Gh_D11G2046          ------------------------------------------------------------
Gh_A11G1928          ------------------------------------------------------------
Gh_A06G0175          ------------------------------------------------------------
Gh_D06G0169          ------------------------------------------------------------
Gh_D12G1804          DKFREGLRIEEEREDE------GENS---VGEL----------------------SEGDD
Gh_Sca005786G01      DKFREGLRIKEEGEDE------GENG---VGEL----------------------SEGDD
Gh_A03G0820          EKFRK-IRLNNQ---T------FQDR---VGSV----------------------K-G-V
Gh_D02G2408          EKFRK-IRLNNQ---T------FQDR---VGSV----------------------KGG-V
Gh_D07G1883          SVAIPPHVWSNLFPVDAPNIPLVEVR---YVRL----------------------SKGTY
Gh_A07G1674          SVAIPPHVWSNLFPVDAPNIPLVEVR---YVRL----------------------SKGTY
Gh_D03G0179          SKGRQCVRWANEGDV----YCC--VH---LASR----------------------FTGSF
Gh_A02G1540          SKGRQCVRWANEGDV----YCC--VH---LASR----------------------FTGSF
Gh_D03G0180          SKGRQCVRWANEGDV----YCC--VH---LASR----------------------FTGSF
Gh_A02G1539          SKGRQCVRWANEGDV----YCC--VH---LASR----------------------FTGTF
Gh_D13G1910          -------------------KPK--VA---VASV----------------------FGN--
Gh_A13G2297          -------------------KPK--VA---VASV----------------------FGN--
Gh_A09G0878          --------SPKSGGQ----FSC--GS---CSKS----------------------FGS--
Gh_D09G0901          --------SPKSGGQ----FSC--GS---CSKS----------------------FGS--
Gh_D05G0849          --------SPKSGGQ----FSC--GS---CNKS----------------------FGS--
Gh_A05G0702          --------SPKSGGQ----FSC--GS---CNKS----------------------FGS--
Gh_D01G2032          AAAQVKQT-PKSGGS----FPC--KS---CGRS----------------------FGS--
Gh_D11G3254          -TGQVNQSAKPGGSS----FPC--KS---CGRS----------------------FGS--
Gh_A11G2870          -TGQVNQSPKPGGSS----FLC--KS---CGRS----------------------FGS--
Gh_D05G1175          ------------------------------------------------------------
Gh_A05G3867          ------------------------------------------------------------
Gh_A05G2341          ------------------------------------------------------------
Gh_D05G2606          ------------------------------------------------------------
Gh_A12G0454          ------------------------------------------------------------
```

```
Gh_D12G0457        ------------------------------------------------------------
Gh_A03G1535        ------------------------------------------------------------
Gh_D01G1781        ------------------------------------------------------------
Gh_A01G1532        ------------------------------------------------------------
Gh_A10G0992        ------------------------------------------------------------
Gh_D10G2570        ------------------------------------------------------------
Gh_A06G0337        ------------------------------------------------------------
Gh_D06G0368        ------------------------------------------------------------
Gh_D05G3801        ------------------------------------------------------------
Gh_A05G1995        ------------------------------------------------------------
Gh_D07G0542        ------------------------------------------------------------
Gh_A07G0477        ------------------------------------------------------------
Gh_A05G2985        ------------------------------------------------------------
Gh_D04G0776        ------------------------------------------------------------
Gh_D05G0663        ------------------------------------------------------------
Gh_A05G3915        ------------------------------------------------------------
Gh_D07G0016        ------------------------------------------------------------
Gh_A07G0010        ------------------------------------------------------------
Gh_D12G2446        KHGTNRERFAPIGNG----NKIWSQKPKAVNEGESLKIRMEKQAANQLNQNKNHE-----
Gh_A12G2312        KHGTNRERFAPIGNG----NKIWSQKPKAANEGESLKIRVEKQAANQLNQNNNHE-----
Gh_D08G1069        KHGTNRERIAAMGNS----NKMWSRKSKAIDDGDSLQIKAGKQATNQPDQNKNPE-----
Gh_A08G2455        KHGTNRERIAAMGNS----NKMWSRKPKAIDDGDSLQIKAGKQATNQPDQNKNPE-----
Gh_D07G2319        ------------------------------------------------------------
Gh_A07G2108        ------------------------------------------------------------
Gh_A05G2549        ----------PFAAA----DSC---------DG-TVSQNKAQKQEIRINARKEV-LSGSF
Gh_D05G2825        ----------PFAAA----DSC---------DG-TVSQNKAQKQEIQINARKEVLLSGSF
Gh_A07G2281        -DSQYQIRRIPRGKQ----MKCMERDD-AFSDD-SVE-DNSQQQHQRILSSKAAKFTE--
Gh_D07G0545        -DSQYQIRRIPRGKQ----MKCMERYD-AFSDD-SVE-DNSQQQHRRILSSKAAKFTE--
Gh_D09G2376        -GSQTQHRRIPSGKQ----MKCTERED-EFSDD-SLE-GNPQQQHRRIAQRKVSKFSDQE
Gh_A09G2168        -DSQTQHRRIPSGKQ----MKCMERED-EFSDD-SLE-DNPQQQHRRIAQRKVSKFSDQE

Gh_A06G0249        ---LL-RDYRGEPLCAF-----------VCK-------------APQGDT------FLVE
Gh_D02G2008        DKTKI-PQF-RCTICNI-----------TCG-------------RSE--D------LNSH
Gh_A03G1559        DKTKI-PQF-RCTICNI-----------TCG-------------RSE--D------LNSH
Gh_A01G1676        ------------------------------------------------------------
Gh_D01G1926        ------------------------------------------------------------
Gh_A09G1153        ------------------------------------------------------------
Gh_D09G1159        ------------------------------------------------------------
Gh_D11G2046        ------------------------------------------------------------
Gh_A11G1928        ------------------------------------------------------------
Gh_A06G0175        ------------------------------------------------------------
Gh_D06G0169        ------------------------------------------------------------
Gh_D12G1804        GFFDA-DA----------------------------------------------------
Gh_Sca005786G01    GFFDA-DA----------------------------------------------------
Gh_A03G0820        EFL---------------------------------------------------------
Gh_D02G2408        EFL---------------------------------------------------------
Gh_D07G1883        AKLQP-DG-IGF---------S-----DLPNHKAILETSLRQHATLSQDD------VLTV
Gh_A07G1674        AKLQP-DG-IGF---------S-----DLPNHKAILETSLRQHATLSQDD------VLTV
Gh_D03G0179        SKIEA-TPAVGTPMCEGTTVLG-----TRCKHRSLYGSSFCKKHRPKSDA------NNSS
Gh_A02G1540        SKIEA-TPPADTPMCEGTTVLG-----TRCKHRSLYGSSFCKKHRPKSDA------NNSC
Gh_D03G0180        SKIEV-TAPVDTPMCEGTTVLG-----TRCKHRSLYGSSFCKKHRPKSDA------NNSC
Gh_A02G1539        SKIEV-TTPVDTPMCEGTTVLG-----TRCKHRSLYGSSFCKKHRPKSDA------NNSC
Gh_D13G1910        ------------------------------------------------------------
Gh_A13G2297        ------------------------------------------------------------
Gh_A09G0878        ------------------------------------------------------------
Gh_D09G0901        ------------------------------------------------------------
Gh_D05G0849        ------------------------------------------------------------
Gh_A05G0702        ------------------------------------------------------------
Gh_D01G2032        ------------------------------------------------------------
Gh_D11G3254        ------------------------------------------------------------
Gh_A11G2870        ------------------------------------------------------------
Gh_D05G1175        ------------------------------------------------------------
```

```
Gh_A05G3867          ------------------------------------------------------------
Gh_A05G2341          ------------------------------------------------------------
Gh_D05G2606          ------------------------------------------------------------
Gh_A12G0454          ------------------------------------------------------------
Gh_D12G0457          ------------------------------------------------------------
Gh_A03G1535          ------------------------------------------------------------
Gh_D01G1781          ------------------------------------------------------------
Gh_A01G1532          ------------------------------------------------------------
Gh_A10G0992          ------------------------------------------------------------
Gh_D10G2570          ------------------------------------------------------------
Gh_A06G0337          ------------------------------------------------------------
Gh_D06G0368          ------------------------------------------------------------
Gh_D05G3801          ------------------------------------------------------------
Gh_A05G1995          ------------------------------------------------------------
Gh_D07G0542          ------------------------------------------------------------
Gh_A07G0477          ------------------------------------------------------------
Gh_A05G2985          ------------------------------------------------------------
Gh_D04G0776          ------------------------------------------------------------
Gh_D05G0663          ------------------------------------------------------------
Gh_A05G3915          ------------------------------------------------------------
Gh_D07G0016          ------------------------------------------------------------
Gh_A07G0010          ------------------------------------------------------------
Gh_D12G2446          ----LLIGSISVTLENYSNHDGNNLAE-ACD-RSL---AECRIPKNNVQEKSSKLDPVQG
Gh_A12G2312          ----LLIGSISVTLENYSNHDGNNLAE-ACD-RSL---AECRIPKNNVQEKSSKLDPFQG
Gh_D08G1069          ----LVIGSISVPL-------GKNLAE-THD-RCP---AECQTPKNNVQESFSKHDHVQI
Gh_A08G2455          ----LIIGSISVPL-------GKNLGE-THD-RCP---AECQ-PKNNVQERFSKHDHVQI
Gh_D07G2319          ------------------------------------------------------------
Gh_A07G2108          ------------------------------------------------------------
Gh_A05G2549          TSAGIDHQSTDLSVEEYSTISKNPGAEEDCH-TDV--------T-LDVEVFQEIQ-ATKG
Gh_D05G2825          TSAGIDHQSTDLSVEEYSTISKNPGAEEDCH-TDV--------A-LDVEVLQDIQ-ATKG
Gh_A07G2281          ------------------------------R-RRV--------HRSQLTEFIEMEDAVSS
Gh_D07G0545          ------------------------------R-RRV--------HRSQLTEFIETEDAVSS
Gh_D09G2376          ------------DIVSFDSLKGN---SHRQH-RRI--------PRSQLTQFIEREDAGSS
Gh_A09G2168          ------------DIVSFDSLKGN---SHRQH-RRI--------PRSQLTQFIEREDAGSS

Gh_A06G0249          LWP------------------IWRGLVLASGLGVKVIWVESDSKSVVKTINQ--------
Gh_D02G2008          LWG---------------------------------------------------------
Gh_A03G1559          LWG---------------------------------------------------------
Gh_A01G1676          ------------------------------------------------------------
Gh_D01G1926          ------------------------------------------------------------
Gh_A09G1153          ------------------------------------------------------------
Gh_D09G1159          ------------------------------------------------------------
Gh_D11G2046          ------------------------------------------------------------
Gh_A11G1928          ------------------------------------------------------------
Gh_A06G0175          ------------------------------------------------------------
Gh_D06G0169          ------------------------------------------------------------
Gh_D12G1804          -----------------------------GD--------EDEEEVEVDSANDH---DE-E
Gh_Sca005786G01      -----------------------------GD--------EDEEEVEVDSANDH---DE-E
Gh_A03G0820          ------------------------------------------------------------
Gh_D02G2408          ------------------------------------------------------------
Gh_D07G1883          KYGELTYKLRVLELKPSSSISVL------------------ETDIEVDIVNPGVESERTD
Gh_A07G1674          KYGELTYKLRVLELKPSSSISVL------------------ETDIEVDIVNPGVESERTD
Gh_D03G0179          HSPEYTQKRKHLEIIQSSETTLCRDIVLVGD-NESPLQVEPVSVIEVDALHRG------N
Gh_A02G1540          HSPENTRKRKRLEIIQSSETTLCRDIVLVGD-NDSPLQVEPVSVIEADALHRG------D
Gh_D03G0180          HSPENTRKRKHLEIIPSSETTFCRDIVLVGD-NESPLQVEPVSVIEADALHRG------N
Gh_A02G1539          HSPENKRKRKHLEIIQSSETTFCRDIVLVGD-NESPLQVEPVSVIEADALHRG------N
Gh_D13G1910          ------------------------------------------------------------
Gh_A13G2297          ------------------------------------------------------------
Gh_A09G0878          ------------------------------------------------------------
Gh_D09G0901          ------------------------------------------------------------
Gh_D05G0849          ------------------------------------------------------------
Gh_A05G0702          ------------------------------------------------------------
```

```
Gh_D01G2032          ------------------------------------------------------------
Gh_D11G3254          ------------------------------------------------------------
Gh_A11G2870          ------------------------------------------------------------
Gh_D05G1175          ------------------------------------------------------------
Gh_A05G3867          ------------------------------------------------------------
Gh_A05G2341          ------------------------------------------------------------
Gh_D05G2606          ------------------------------------------------------------
Gh_A12G0454          ------------------------------------------------------------
Gh_D12G0457          ------------------------------------------------------------
Gh_A03G1535          ------------------------------------------------------------
Gh_D01G1781          ------------------------------------------------------------
Gh_A01G1532          ------------------------------------------------------------
Gh_A10G0992          ------------------------------------------------------------
Gh_D10G2570          ------------------------------------------------------------
Gh_A06G0337          ------------------------------------------------------------
Gh_D06G0368          ------------------------------------------------------------
Gh_D05G3801          ------------------------------------------------------------
Gh_A05G1995          ------------------------------------------------------------
Gh_D07G0542          ------------------------------------------------------------
Gh_A07G0477          ------------------------------------------------------------
Gh_A05G2985          ------------------------------------------------------------
Gh_D04G0776          ------------------------------------------------------------
Gh_D05G0663          ------------------------------------------------------------
Gh_A05G3915          ------------------------------------------------------------
Gh_D07G0016          ------------------------------------------------------------
Gh_A07G0010          ------------------------------------------------------------
Gh_D12G2446          VTNR--STIKFWRPVSRQE---------------S-------------------------
Gh_A12G2312          VTNR--STIKFWRPVSRHE---------------S-------------------------
Gh_D08G1069          VANR--STIKFWRPVSRQE---------------R-------------------------
Gh_A08G2455          VANR--STIKFWRPVSRQE---------------R-------------------------
Gh_D07G2319          ------------------------------------------------------------
Gh_A07G2108          ------------------------------------------------------------
Gh_A05G2549          TGGDEVITCSNLPMQEKQP---------------TPVMMEACSEIQQD------------
Gh_D05G2825          TGGDEVITCSDLPIQEKQP---------------TPVMMEACSEIQQD------------
Gh_A07G2281          DSPDDNSSLRQPRRIPRSK---------------QPEILEREDAISD-------------
Gh_D07G0545          DSPDDNSSLRQPRRIPRSK---------------QPEILEREDAISD-------------
Gh_D09G2376          DSPDDS-SLQQLTRILRSK---------------HAKILEKEDAVSD-------------
Gh_A09G2168          DSPDDS-SLQQLRRILRSK---------------HAKILEKEGAVSD-------------

Gh_A06G0249          -----EQPYGP-----KSSQCLRQIWK------LLTKFENY------------------R
Gh_D02G2008          ------------------------------------------------------------
Gh_A03G1559          ------------------------------------------------------------
Gh_A01G1676          -------------MGTPEFPDLGRHCS--VQHCKQIDFLPF-TCDRCDLS------YCLE
Gh_D01G1926          -------------MGTPEFPDLGRHCS--VQHCKQIDFLPF-TCDRCDLS------YCLE
Gh_A09G1153          -------------MGTPQFPDLGKHCS--VEDCKQIDFLPF-TCDRCHLV------YCLE
Gh_D09G1159          -------------MGTPQFPDLGKHCS--VEDCKQIDFLPF-TCDRCHLV------YCLE
Gh_D11G2046          ------------------------------------------------------------
Gh_A11G1928          ------------------------------------------------------------
Gh_A06G0175          ------------------------------------------------------------
Gh_D06G0169          ------------------------------------------------------------
Gh_D12G1804          NSILEAMVSGQKDKKNVSFKSEDMV--------SPTGFHVKDESDEGEFI------NYGN
Gh_Sca005786G01      NSILEAMVSGQKDKKSVYFKSEDMV--------SPTGFHVKDESDEGEFM------NYGN
Gh_A03G0820          ---------------------------------ELCGFEKV---EGEEFL------FLSR
Gh_D02G2408          ---------------------------------ELCGFEKV---EGEEFL------FLSR
Gh_D07G1883          QYVLKPLAFGASESGLVE---EGNY--------MYYKFS--------------------I
Gh_A07G1674          QYVLKPLAFGASESGLVE---EGNY--------MYYKFS--------------------I
Gh_D03G0179          SLIEKPEHSGKDHDGTELMHCIGLY--------SNNGFDPCQESPKRHSL------YCDK
Gh_A02G1540          SLIEKPEHSGKDHDGTELMHCIGLY--------SNNGFDPCQESPKRHSL------YCDK
Gh_D03G0180          SLIEKPEHSGKDHDSTELLHCIGLY--------SNNGFDPCQESPKRHSL------YCDK
Gh_A02G1539          SLIEKPEHSGKDHDSTELLHCIGLY--------SNNGFDPCQESPKRHSL------YCDK
Gh_D13G1910          ------------------------------------------------------------
Gh_A13G2297          ------------------------------------------------------------
```

```
Gh_A09G0878          ------------------------------------------------------------
Gh_D09G0901          ------------------------------------------------------------
Gh_D05G0849          ------------------------------------------------------------
Gh_A05G0702          ------------------------------------------------------------
Gh_D01G2032          ------------------------------------------------------------
Gh_D11G3254          ------------------------------------------------------------
Gh_A11G2870          ------------------------------------------------------------
Gh_D05G1175          ------------------------------------------------------------
Gh_A05G3867          ------------------------------------------------------------
Gh_A05G2341          ------------------------------------------------------------
Gh_D05G2606          ------------------------------------------------------------
Gh_A12G0454          ------------------------------------------------------------
Gh_D12G0457          ------------------------------------------------------------
Gh_A03G1535          ------------------------------------------------------------
Gh_D01G1781          ------------------------------------------------------------
Gh_A01G1532          ------------------------------------------------------------
Gh_A10G0992          ------------------------------------------------------------
Gh_D10G2570          ------------------------------------------------------------
Gh_A06G0337          ------------------------------------------------------------
Gh_D06G0368          ------------------------------------------------------------
Gh_D05G3801          ------------------------------------------------------------
Gh_A05G1995          ------------------------------------------------------------
Gh_D07G0542          ------------------------------------------------------------
Gh_A07G0477          ------------------------------------------------------------
Gh_A05G2985          ------------------------------------------------------------
Gh_D04G0776          ------------------------------------------------------------
Gh_D05G0663          ------------------------------------------------------------
Gh_A05G3915          ------------------------------------------------------------
Gh_D07G0016          ------------------------------------------------------------
Gh_A07G0010          ------------------------------------------------------------
Gh_D12G2446          ------------------------------------------------------------
Gh_A12G2312          ------------------------------------------------------------
Gh_D08G1069          ------------------------------------------------------------
Gh_A08G2455          ------------------------------------------------------------
Gh_D07G2319          ------------------------------------------------------------
Gh_A07G2108          ------------------------------------------------------------
Gh_A05G2549          -------------SSSSKKPCVGATADADSHENDPNRYEKNEESASCCR---TPINQTTI
Gh_D05G2825          -------------SSSSKKLCVGATADADSHENDPNRYEKNEESASCCH---TPINQTTI
Gh_A07G2281          ----------------------------DSLDGSH-------------------------
Gh_D07G0545          ----------------------------DSLDGSH-------------------------
Gh_D09G2376          ----------------------------DSDDTSPQQLRKTPRSRQGKSIESEDVVSYDS
Gh_A09G2168          ----------------------------DSDDTSPQQFRKTPRSRQGKSIESEDVVSYDS

Gh_A06G0249          VTHSWRETNKA-------ADH------------LSRMVLRENDV----------------
Gh_D02G2008          ------RKHLA-------------------------------------------------
Gh_A03G1559          ------RKHLA-------------------------------------------------
Gh_A01G1676          HRS-YIKHQC------------------------SKAGNNDVTVVICPLCA---------
Gh_D01G1926          HRS-YIKHQC------------------------SKAGNNDVTVVICPLCA---------
Gh_A09G1153          HRS-YIKHHC------------------------PKADKKDVTVVICPLCA---------
Gh_D09G1159          HRS-YIKHHC------------------------PKADKKDVTVVICPLCA---------
Gh_D11G2046          ------------------------------------------------------------
Gh_A11G1928          ------------------------------------------------------------
Gh_A06G0175          ---------------------------------------------MNSLCNSLKS-----
Gh_D06G0169          ---------------------------------------------MNSLCNSLKS-----
Gh_D12G1804          RKN--RRRNRT---------G------------KKEKGKKNSDDAMKTDVN--ETKSKN-
Gh_Sca005786G01      RKN--RRRNRT---------G------------KKEKGKKNSDDAMKIDVN--ETKSKN-
Gh_A03G0820          NKVDMQVLNSA---------G------------SE----------LN-------------
Gh_D02G2408          NKVDMQVLNSA---------G------------SE----------LN-------------
Gh_D07G1883          DDDTWEK-----------------------------LVSDDVKIEVKIDAEANGG-----
Gh_A07G1674          DDDTWEK-----------------------------LVSDDVKIEVKIDAEANGG-----
Gh_D03G0179          HLPSWLKRARN---------G------------KSRIVSREVFVDLLKDCDSLEQKLHLH
Gh_A02G1540          HLPSWLKRARN---------G------------KSRIVSREVFVDLLKDCDSLEQKLHLH
```

```
Gh_D03G0180        HLPSWLKRARN---------G------------KSRIVSREVFVDLLKDCDSLEQKFHLH
Gh_A02G1539        HLPSWLKHARN---------G------------KSRIVSREVFVDLLKDCDSLVQKLHLH
Gh_D13G1910        ------------------------------------------------------------
Gh_A13G2297        ------------------------------------------------------------
Gh_A09G0878        ------------------------------------------------------------
Gh_D09G0901        ------------------------------------------------------------
Gh_D05G0849        ------------------------------------------------------------
Gh_A05G0702        ------------------------------------------------------------
Gh_D01G2032        ------------------------------------------------------------
Gh_D11G3254        ------------------------------------------------------------
Gh_A11G2870        ------------------------------------------------------------
Gh_D05G1175        ------------------------------------------------------------
Gh_A05G3867        ------------------------------------------------------------
Gh_A05G2341        ------------------------------------------------------------
Gh_D05G2606        ------------------------------------------------------------
Gh_A12G0454        ------------------------------------------------------------
Gh_D12G0457        ------------------------------------------------------------
Gh_A03G1535        ------------------------------------------------------------
Gh_D01G1781        ------------------------------------------------------------
Gh_A01G1532        ------------------------------------------------------------
Gh_A10G0992        ------------------------------------------------------------
Gh_D10G2570        ------------------------------------------------------------
Gh_A06G0337        ------------------------------------------------------------
Gh_D06G0368        ------------------------------------------------------------
Gh_D05G3801        ------------------------------------------------------------
Gh_A05G1995        ------------------------------------------------------------
Gh_D07G0542        ------------------------------------------------------------
Gh_A07G0477        ------------------------------------------------------------
Gh_A05G2985        ------------------------------------------------------------
Gh_D04G0776        ------------------------------------------------------------
Gh_D05G0663        ------------------------------------------------------------
Gh_A05G3915        ------------------------------------------------------------
Gh_D07G0016        ------------------------------------------------------------
Gh_A07G0010        ------------------------------------------------------------
Gh_D12G2446        -----------------------------KSSLPVQNGASESEVQVIAEKDGART-----
Gh_A12G2312        -----------------------------KSSSPVQNGASESEVQVIAEKDGART-----
Gh_D08G1069        -----------------------------KSSLPVQNGIKEV--EVIAENDGVQI-----
Gh_A08G2455        -----------------------------KSSLPVQNGIRDVEVEVIAENDGVQI-----
Gh_D07G2319        --------------------------------------------MAMV------------
Gh_A07G2108        --------------------------------------------MAMV------------
Gh_A05G2549        PIQKYSRAHRDTYATVNVNDGTGVCSS-------VENGDLE---SAMVNCKSNAMGRKRK
Gh_D05G2825        PIQNYSRAHRETCATVNVNDGTDVCSS-------VENGDLE---SAMVNCKSNAMGRKRK
Gh_A07G2281        ------QSNRSL--RNRKKKGPTLCQMKQETAQNVKQVKHRSTEQVISQQVKQETPQNWN
Gh_D07G0545        ------QSNRSL--RNRKKKGPTLCQMKRETAQNVKQVKRRSTEQVISQHVKQETPQNQN
Gh_D09G2376        SDENYGQPSSSL--RSRKKKAPTPRQTKQEAPKNVKQGKRRTTKQVISQQSKQDTPRNRT
Gh_A09G2168        SDENYGQPNSSF--RSRKKKAPTPRQTKQKAPKNVKQGKRRTTKQVISQQSKQDTRRNRN

Gh_A06G0249        ---VLWPADFPDTLNSIIK----------DDAQGKTYLRR--------------------
Gh_D02G2008        ------------K----------------LNSLGRGQLA---------------------
Gh_A03G1559        ------------N---IQK----------LNNLGEGQLA---------------------
Gh_A01G1676        ----K---------GVRLIPNEDPNISWETHV----------------------------
Gh_D01G1926        ----K---------GVRLIPNEDPNISWENHV----------------------------
Gh_A09G1153        ----K---------GVRLVPDEDPNITWEMHV----------------------------
Gh_D09G1159        ----K---------GVRLIPDEDPNITWEMHV----------------------------
Gh_D11G2046        -----MLHLFPSI-----------------------------------------------
Gh_A11G1928        -----MLHLFPSI-----------------------------------------------
Gh_A06G0175        --LNVNPTFFPTK-----------------------------------------------
Gh_D06G0169        --LHVNPTFFPTK-----------------------------------------------
Gh_D12G1804        ----------------------------------------EEASVSDRT--------LH-
Gh_Sca005786G01    ----------------------------------------KEVSVSDTT--------LH-
Gh_A03G0820        -------------------------------------------SAINNP--------FF-
Gh_D02G2408        -------------------------------------------SAINNP--------FF-
```

```
Gh_D07G1883          -DTDLYVSKHP------LIFPNRHQHEWSSHDVGSK----TLILSSNDR--------NL-
Gh_A07G1674          -DTDLYVSKHP------LIFPNRHQHEWSSHDVGSK----TLILSSKDR--------NL-
Gh_D03G0179          QACELFYKLFKSILSLRNPVPVDVQLQWALSEASKDFRVGELLMKL-----------VY-
Gh_A02G1540          QACELFYKLFKSILSLRNPVPVDVQLQWALSEASKDYIVGEFLMKL-----------VY-
Gh_D03G0180          QACELFYKLFKSIFSLR-TVPVDVQLQWALSEASKDYRVGEFLMKL-----------VY-
Gh_A02G1539          QACELFYKLFKSIFSLRNPVPVDVQLQWALSEASKDYRVGEFLMKL-----------VY-
Gh_D13G1910          ------------------------------------------------------------
Gh_A13G2297          ------------------------------------------------------------
Gh_A09G0878          ------------------------------------------------------------
Gh_D09G0901          ------------------------------------------------------------
Gh_D05G0849          ------------------------------------------------------------
Gh_A05G0702          ------------------------------------------------------------
Gh_D01G2032          ------------------------------------------------------------
Gh_D11G3254          ------------------------------------------------------------
Gh_A11G2870          ------------------------------------------------------------
Gh_D05G1175          ------------------------------------------------------------
Gh_A05G3867          ------------------------------------------------------------
Gh_A05G2341          ------------------------------------------------------------
Gh_D05G2606          ------------------------------------------------------------
Gh_A12G0454          ------------------------------------------------------------
Gh_D12G0457          ------------------------------------------------------------
Gh_A03G1535          ------------------------------------------------------------
Gh_D01G1781          ------------------------------------------------------------
Gh_A01G1532          ------------------------------------------------------------
Gh_A10G0992          ------------------------------------------------------------
Gh_D10G2570          ------------------------------------------------------------
Gh_A06G0337          ------------------------------------------------------------
Gh_D06G0368          ------------------------------------------------------------
Gh_D05G3801          ------------------------------------------------------------
Gh_A05G1995          ------------------------------------------------------------
Gh_D07G0542          ------------------------------------------------------------
Gh_A07G0477          ------------------------------------------------------------
Gh_A05G2985          ------------------------------------------------------------
Gh_D04G0776          ------------------------------------------------------------
Gh_D05G0663          ------------------------------------------------------------
Gh_A05G3915          ------------------------------------------------------------
Gh_D07G0016          ------------------------------------------------------------
Gh_A07G0010          ------------------------------------------------------------
Gh_D12G2446          -SSNETCLR-------------------SCSTDGIDGVVSMDLISTLKESVQPESSQFDS
Gh_A12G2312          -SCNETCLR-------------------SCSTDGIDGVVSMDLISTLKERVQPESLQFDS
Gh_D08G1069          -SSNESYRR-------------------SSAVDGSDGVVRMNLSSTLKESVQPGGLQFDG
Gh_A08G2455          -SSNESYRR-------------------SSAVDGSDGVLRMNLSSTLKESVQPGGLQFDS
Gh_D07G2319          ----VDYKH------------------DSS-------FP--YWTSIRRR-FA-PDSPFFS
Gh_A07G2108          ----VDYKH------------------DSS-------FP--YWTSIRRR-FA-PDSPFFS
Gh_A05G2549          RELEET---------------------CQKV--GSDGFIRSPCEGLRPRAWKDATSSFDA
Gh_D05G2825          RELEET---------------------CQKV--GSDGFIRSPCEGLRPRAWKDATSSFDA
Gh_A07G2281          TKIKQTARH------------------CSSSSDEDEIEEGGPSTRLRKRTRKPPKRPETK
Gh_D07G0545          TKTKQTARH------------------CSSSSDEDEI-EGGPSTRLRKRTRKPLKQPETK
Gh_D09G2376          TKIEQSARQ------------------DNSY-D-DDEIEGGPSTRLRKRAQKPSKEPETK
Gh_A09G2168          TKIEQSARQ------------------DNSY-D-EDEIEGGPSTRLRKRARKPSKEPETK


Gh_A06G0249          ------------------------------------------------------------
Gh_D02G2008          ------------------------------------------------------------
Gh_A03G1559          ------------------------------------------------------------
Gh_A01G1676          -----------------NTECDPSNYDKVIKKMKCPV-------SRCKEVLTFSNTIKCK
Gh_D01G1926          -----------------NTECDPSNYDKVTKKKKCPV-------RRCKEVLTFSNTIKCK
Gh_A09G1153          -----------------NIECDPSNYDKVTKKKKCPV-------PGCREVLTFSNTIKCR
Gh_D09G1159          -----------------NTECDPSNYDKVTKKKKCPV-------PGCREVLTFSNTIKCR
Gh_D11G2046          ------NNSVQSIRFKTLN--PC--I--S-------LS-----RLCHFKAKRGFD-----
Gh_A11G1928          ------SNSVQSIIFKTLN--PC--I--S-------LS-----RLCHFKAKRGFD-----
Gh_A06G0175          ------PELSFSFSFSPISAPPFKSK--S-------SR-----TRCRLTVKADANAVEID
Gh_D06G0169          ------PELPFSFSLSPISAPPFKSK--S-------SP-----TRCRLTVKAEANAVEID
```

```
Gh_D12G1804          ---GEEKQLVEDEGSSGEKDDKLGNG---DKVSKQPTD-----RKGNTK--KETNT----
Gh_Sca005786G01      ---GEEKQLVEDEGSSGEKDDKLGNG---DKVSKQPVD-----RKGNTK--KETNT----
Gh_A03G0820          ---GVL------------------------------------------------------
Gh_D02G2408          ---GVL------------------------------------------------------
Gh_D07G1883          ---GTGTYSIAVYGFKGTTKYQVSVH--VQENSKHKVG-----QQATHSSSMEVDTVECR
Gh_A07G1674          ---GTGTYSLAVYGFKGTTKYQVSVH--VQENSKHKVG-----QQATHSSSMDVDTVECR
Gh_D03G0179          ---SEKERLQSLWGFTGDKGTPSSSF--MEGPVPLPL--------AINDSFDDDKTIKCK
Gh_A02G1540          ---SEKERLQSLWGFTGDKGTPSSSF--VEEPVPLPL--------AINDSFDDDKTIKCK
Gh_D03G0180          ---SEKERLQSLWGFTGDKGTPLSSF--VEEPVPLPL--------AINDSFDDDKTIKCK
Gh_A02G1539          ---SEKERLQSLWGFTGDKGTPLSSF--VEEPVPLPL--------AINDSFDDDKTIKCK
Gh_D13G1910          ------------------------------------------------------------
Gh_A13G2297          ------------------------------------------------------------
Gh_A09G0878          ------------------------------------------------------------
Gh_D09G0901          ------------------------------------------------------------
Gh_D05G0849          ------------------------------------------------------------
Gh_A05G0702          ------------------------------------------------------------
Gh_D01G2032          ------------------------------------------------------------
Gh_D11G3254          ------------------------------------------------------------
Gh_A11G2870          ------------------------------------------------------------
Gh_D05G1175          --------------------------------------------------------CGAT
Gh_A05G3867          --------------------------------------------------------CGAT
Gh_A05G2341          --------------------------------------------------------CGAS
Gh_D05G2606          --------------------------------------------------------CGAS
Gh_A12G0454          ------------------------------------------------------------
Gh_D12G0457          ------------------------------------------------------------
Gh_A03G1535          --------------------------------------------------------VAAY
Gh_D01G1781          ------------------------------------------------------------
Gh_A01G1532          ------------------------------------------------------------
Gh_A10G0992          ------------------------------------------------------------
Gh_D10G2570          ------------------------------------------------------------
Gh_A06G0337          ----------------------------------------------DVDLFLFYVLCECD
Gh_D06G0368          ----------------------------------------------DVDLFLFYVLCECD
Gh_D05G3801          ----------------------------------------------DVDLFLFYVLCECD
Gh_A05G1995          ----------------------------------------------DVDLFLFYVLCECD
Gh_D07G0542          ----------------------------------------------ARRLHELFLRQFCD
Gh_A07G0477          ----------------------------------------------ARRLHELFLRQFCD
Gh_A05G2985          ----------------------------------------------SGRLHEFFLRQFCD
Gh_D04G0776          ----------------------------------------------TGRLHEFFLRQFCD
Gh_D05G0663          ------------------------------------------------------------
Gh_A05G3915          ------------------------------------------------------------
Gh_D07G0016          ------------------------------------------------------------
Gh_A07G0010          ------------------------------------------------------------
Gh_D12G2446          --HTAKAILAQRWKEALAGEHVTL----VLTPNPDPPGCLEIEADSSKKLVVKARAFEAS
Gh_A12G2312          --HTAKAILAQRWKEALAGEHVTL----VLTPNLDPPGCSEIEADSSEKLVVKARAFEAS
Gh_D08G1069          --NAAKAFLAERWKEAVAGEHVTL----VLSSNPKPPGCSGVEIDSSEKWMVKAGACEAS
Gh_A08G2455          --NAAKAFLAERWKEAVAGEHVTL----VLSSNPKPPGCSGVEIDSSEKWMVKAGACEAS
Gh_D07G2319          SGNIERELLAK-------------------------------------------------
Gh_A07G2108          SGNIERELLAK-------------------------------------------------
Gh_A05G2549          -DKASSEGLQTK-----ETRKP--------------ST------HAHRKIIIKKGSHGCD
Gh_D05G2825          -DKASSEGLQTK-----ETRKP--------------ST------HAHRKIIIKKGSHGCD
Gh_A07G2281          -PKEKKQAGKKKVKNALNSKTL--------------SG------QNSAKVRDEEVEYQCD
Gh_D07G0545          -PKEKKQAGKKKVKNALNSKTL--------------SG------QNSAKVRDEEVEYQCD
Gh_D09G2376          -PKVKKQALKNKAKTA-----------------------------SNAKVRDEEAEYQCD
Gh_A09G2168          -PKVKKQALKNKAKSA-----------------------------SNAKVRDEGAEYQCD


Gh_A06G0249          ------------------------------------------------------------
Gh_D02G2008          ------------------------------------------------------------
Gh_A03G1559          ------------------------------------------------------------
Gh_A01G1676          D--CSVDHC--------LKHRFGLDHDCPGP-KKPA------------------------
Gh_D01G1926          D--CSVDHC--------LKHRFGLDHDCPGP-KKPA------------------------
Gh_A09G1153          D--CTLDHC--------LKHRFGPDHKCPGP-KKSDPGFPFMGL----LSRSRKES----
Gh_D09G1159          D--CTLDHC--------LKHRFGPDHKCPGP-KKSDPGFPFMGL----LSRSRKES----
```

```
Gh_D11G2046           ------SLNQTSSVQNSVAVFWDLDNK--PP-NAF-PPFEAV------------------
Gh_A11G1928           ------SLNQTSSVQNSVAVFWDLDNK--PP-NAF-PPFETV------------------
Gh_A06G0175           M--VRNKQGVYAPRQKKVVVLWDLDNK--PP-RG--PPYEAA------------------
Gh_D06G0169           M--VRNKQGVYAPKQKKVVVLWDLDNK--PP-RG--PPYEAA------------------
Gh_D12G1804           -----------------KSNKSSKGKKAKAT-AKH-SSNVCETCGEEFQSKNKLHKHLGD
Gh_Sca005786G01       -----------------KSNKSSKGKKAKAT-AKH-SSNVCETCGEEFQSKNKLHKHLGD
Gh_A03G0820           ------------------------------------------------------------
Gh_D02G2408           ------------------------------------------------------------
Gh_D07G1883           N--CK--HF--I-PSRSIALH---------------EAY----CS----RHNVVCPHAGC
Gh_A07G1674           N--CK--HF--I-PTRSIALH---------------EAY----CS----RHNVVCPHAGC
Gh_D03G0179           M--CS--VE-FL-DDQQLGTHWMENHKKEAQ-RLF-RGYACAICLDSFINKKVLESHVQE
Gh_A02G1540           M--CS--VE-FL-DDQQLGTHWMENHKKEAQ-RLF-RGYACAICLDSFINKKVLESHVQE
Gh_D03G0180           M--CS--VE-FL-DDQQLGTHWMENHKKEAQ-RLF-EGYACAICLDSFTNKKVLESHVQE
Gh_A02G1539           M--CS--VE-FL-DDQQLGTHWMENHKKEAQ-RLF-EGYACAICLDSFTNKKVLESHVQE
Gh_D13G1910           --------------------------------------------------DSDDE-----
Gh_A13G2297           --------------------------------------------------DSDDE-----
Gh_A09G0878           --------------------------------------------------EGGLESHKKA
Gh_D09G0901           --------------------------------------------------EGGLESHKKA
Gh_D05G0849           --------------------------------------------------EGGLESHKKA
Gh_A05G0702           --------------------------------------------------EGGLESHKKA
Gh_D01G2032           --------------------------------------------------ENALQSHSKA
Gh_D11G3254           --------------------------------------------------EMALQSHSKA
Gh_A11G2870           --------------------------------------------------ENALQSHSKA
Gh_D05G1175           F-------R---------KPAHLKQHLQSHSLERP-FVCLVDDCHASYRRKD----HLN-
Gh_A05G3867           F-------R---------KPAHLKQHLQSHSLERP-FVCLVDDCHASYRRKD----HLN-
Gh_A05G2341           F-------K---------KPAYLKQHLQSHSLERP-FVCSVEDCHASYRRKD----HLN-
Gh_D05G2606           F-------K---------KPAYLKQHLQSHSLERP-FVCLVEDCHASYRRKD----HLT-
Gh_A12G0454           -------------------------------------------------------MG---
Gh_D12G0457           -------------------------------------------------------MG---
Gh_A03G1535           LQSCFGLHD-AP--LTMDNDGDIAQHIYQMAPEIEGAIQKGDDQRQTEREKEVNLLGMD-
Gh_D01G1781           ---------------------------------------------LNDKEKEPEAPEP--
Gh_A01G1532           ---------------------------------------------LNDKEKEPEAPEP--
Gh_A10G0992           ---------------------------------------------ANMREAVPQLNHAR-
Gh_D10G2570           ---------------------------------------------ANMREAVPLLNHAR-
Gh_A06G0337           DRGCHMVG-YFS--KEKHSE--ESYNLAC---ILTLPPYQRKGYGKFLIAFSYELSKKE-
Gh_D06G0368           DRGCHMVG-YFS--KEKHSE--ESYNLAC---ILTLPPYQRKGYGKFLIAFSYELSKKE-
Gh_D05G3801           DRGCHMVG-YFS--KEKHSE--ESYNLAC---ILTLPPYQRKGYGKFLIAFSYELSKKE-
Gh_A05G1995           DRGCHMVG-YFS--KEKHSE--ESYNLAC---ILTLPPYQRKGYGKFLIAFSYELSKKE-
Gh_D07G0542           AHTCSGKAKPFP--RGGKKQTNLLYMATSILLMMLLPLF-------YLLYYLYQRDMKQE
Gh_A07G0477           AHTCSGKAKPFP--RGGKKQTNLLYMATSILLMMLLPLF-------YLLYYLYQRDMKQE
Gh_A05G2985           AHTCSGGPKPFP--KGQKNRTSMLYISLSVLTLMLLPLF-------YFFIYLYQRGIKSG
Gh_D04G0776           AHTCSGGPKPFP--KGQKKRTSMLYISLSILTLMLLPLF-------YFFIYLYQRGIKSG
Gh_D05G0663           ------------------------------------------------------------
Gh_A05G3915           ------------------------------------------------------------
Gh_D07G0016           ------------------------------------------------------------
Gh_A07G0010           ------------------------------------------------------------
Gh_D12G2446           TAGPA---------------------------RGKFRRKPEKGAKIK-----YIPKQRS-
Gh_A12G2312           TAGPA---------------------------RGKFRRNPEKGAKIK-----YIPKQRS-
Gh_D08G1069           TVGAA---------------------------IAKYRKKPERGAKTK-----YIPKQRS-
Gh_A08G2455           TVGAA---------------------------IAKYRTKPEKGAKTK-----YIPKQRS-
Gh_D07G2319           --QVALDFT-----EDERNQ--LEK---MIDTDARGVFCPIVGCGARLTLLEDFEDHYN-
Gh_A07G2108           --QVALDLT-----EDERNQ--LEK---MIDNDARGIFCPIVGCDARLTLLEDFEDHYN-
Gh_A05G2549           LEGCHMSFK-----TKEE------------LRLHKRNRCPYEGCGKKFRSHKYAVLHQR-
Gh_D05G2825           LEGCHMSFK-----TKEE------------LRLHKRNRCPYEGCGKKFRSHKYAVLHQR-
Gh_A07G2281           MEGCSMSFG-----SKQA------------LLLHKRNICPVKGCGKKFFSHKYLVQHRR-
Gh_D07G0545           MEGCSMSFG-----SKQA------------LILHKRNICPVKGCGKKFFSHKYLVQHRR-
Gh_D09G2376           MEGCTMSFG-----LKQE------------LVLHKKNICPVKGCGKKFFSHKYLVQHRR-
Gh_A09G2168           MEGCTMSFG-----LKQE------------LVLHKKNICPVKGCGKKFFSHKYLVQHRR-


Gh_A06G0249           ------------------------------------------------------------
Gh_D02G2008           ------------------------------------------------------------
Gh_A03G1559           ------------------------------------------------------------
```

```
Gh_A01G1676         -------------------------TTSSSFWATSLLNVASSFRE---------------
Gh_D01G1926         -------------------------TTSSSFWATSLLNVASSFRE---------------
Gh_A09G1153         -------------------KTNQAPATSSSKWATSFLNAASTVRASAEAG--MTKLGSEI
Gh_D09G1159         -------------------KTNQSPATSSSKWATSFLNAASTVRASAEAG--MTKLGSEI
Gh_D11G2046         -------------VKL--KTAASSFGVVRSM---VAYANQ-------HSFSYVPKAVREQ
Gh_A11G1928         -------------VKL--KTAASSFGVVRSM---VAYANQ-------HSFSYVPKAVREQ
Gh_A06G0175         -------------LAL--KRVAEKFGEVVDM---SAYANR-------HAFVHLPQWVLQE
Gh_D06G0169         -------------LAL--KRVAEKFGEVVDM---SAYANR-------HAFVHLPQWVLQE
Gh_D12G1804         SGHAT-LKFR--------------------------------------------------
Gh_Sca005786G01     SGHAT-LKFR--------------------------------------------------
Gh_A03G0820         ------------------------------------------------------------
Gh_D02G2408         ------------------------------------------------------------
Gh_D07G1883         GIVLR-IEETKNHVHC--DKCGQAFHLGEMEKHMKVF--------------H-------E
Gh_A07G1674         GIVLR-IEEAKNHVHC--DKCGQAFHLGEMEKHMKVF--------------H-------E
Gh_D03G0179         RHHVQ-FVEQCMLLRC--ISCGSHFGNTEELWLHVLSTHPVDFRLSKIALEHNPSAGEEP
Gh_A02G1540         RHHVQ-FVEQCMLLRC--ISCGSHFGNTEELWLHVLSTHPVDFRLSKIAQQHNPSASEEP
Gh_D03G0180         RHHVQ-FVEQCMLLRC--ISCGSHFGNTEELWLDVLSAHPVEFRLSKITQQHNLSAGEEP
Gh_A02G1539         RHHVQ-FVEQCMLIQC--ISCGSHFGNTEELWLDVLSAHPVEFRLSKIAQQHNLSAGEEP
Gh_D13G1910         ------------------------------------------------------------
Gh_A13G2297         ------------------------------------------------------------
Gh_A09G0878         KHGGK-------------------------------------------------------
Gh_D09G0901         KHGGK-------------------------------------------------------
Gh_D05G0849         KHGGK-------------------------------------------------------
Gh_A05G0702         KHGGK-------------------------------------------------------
Gh_D01G2032         KHGTQ-A-----------------------------------------------------
Gh_D11G3254         KHGTA-V-----------------------------------------------------
Gh_A11G2870         KHGTA-V-----------------------------------------------------
Gh_D05G1175         ---RHLLQHQGKLFSCPIETCNSKFAFQGNMKRHVKEFHDEDSSSSDAAS----------
Gh_A05G3867         ---RHLLQHQGKLFSCPIENCNSKFAFQGNMKRHVKEFHDEGSSSSDAAS----------
Gh_A05G2341         ---RHLLQHQGKLFKCLIENCNREFAFQGNMKRHIKEFHDEESSDLDAGS----------
Gh_D05G2606         ---RHLLQHQGKLFKCLIENCNREFAFQGNMKRHLKEFHDEESSDLDAGS----------
Gh_A12G0454         ----KKKKRVPSKVWC--YYCDREFDDEKILVQHQK------------------------
Gh_D12G0457         ----KKKKRVPSKVWC--YYCDREFDDEKILVQHQK------------------------
Gh_A03G1535         ---NLAEVQIHDLFEC--HKCNMTFDEKDAYLQHLLSFHQRTT-----------------
Gh_D01G1781         ----EPEPTTEVLFLCSYEGCGKTFIDAGALRKHSHIHGER-------------------
Gh_A01G1532         ----EPEPTTEVLFLCSYEGCGKTFIDAGALRKHSHIHGER-------------------
Gh_A10G0992         ---REQPGSAEELIAC--RICDHIFTSSRALFDHMELHL---------------------
Gh_D10G2570         ---REQPGSAEELIAC--RICDHIFTSSRALFDHMELHL---------------------
Gh_A06G0337         -------GKVGTPER--------PLSDLGLLSYRGYWTRVLLDILKKHKG----------
Gh_D06G0368         -------GKVGTPER--------PLSDLGLLSYRGYWTRVLLDILKKHKG----------
Gh_D05G3801         -------GKVGTPER--------PLSDLGLLSYRGYWTRVLLDILKKHKG----------
Gh_A05G1995         -------GKVGTPER--------PLSDLGLLSYRGYWTRVLLDILKKHKG----------
Gh_D07G0542         TQVLRRVSQVGRKAK--------PS-----------------------------------
Gh_A07G0477         TQVLRRVSQVGRKAK--------PS-----------------------------------
Gh_A05G2985         SQELKRISLSGRKKK--------SF-----------------------------------
Gh_D04G0776         SQELKRISLSGRKKK--------SF-----------------------------------
Gh_D05G0663         ------------------------------------------------------------
Gh_A05G3915         ------------------------------------------------------------
Gh_D07G0016         ------------------------------------------------------------
Gh_A07G0010         ------------------------------------------------------------
Gh_D12G2446         ------AT----------------------------------------------------
Gh_A12G2312         ------AI----------------------------------------------------
Gh_D08G1069         ------AT----------------------------------------------------
Gh_A08G2455         ------AT----------------------------------------------------
Gh_D07G2319         ------TRHT---ASC--SVCSRVYPTSRLLSIHISEAHDSFFQAKVARG----------
Gh_A07G2108         ------ARHT---ASC--SVCSRVYPTSRLLSIHVSEAHDSFFQAKVARG----------
Gh_A05G2549         ------VHDDDRPLKCPWKGCSMSFK----------WAWARTEHIRVHTG----------
Gh_D05G2825         ------VHDDDRPLKCPWKGCSMSFK----------WAWARTEHIRVHTG----------
Gh_A07G2281         ------VHLDDRPLKCPWKGCKMTFK----------WAWARTEHIRVHTG----------
Gh_D07G0545         ------VHLDDRPLKCPWKGCKMTFK----------WAWARTEHIRVHTG----------
Gh_D09G2376         ------VHMDDRPLKCPWKGCKMTFK----------WAWARTEHIRVHTG----------
Gh_A09G2168         ------VHMDDRPLKCPWKGCKMTFK----------WAWARTEHIRVHTG----------
```

```
Gh_A06G0249        ------------------------------------------------------------
Gh_D02G2008        ------------------------------------------------------------
Gh_A03G1559        ------------------------------------------------------------
Gh_A01G1676        --NRQVARDGAGLRR-----SSSGSAGQMEEC---PQCRVKFSSVTALVEHVKKVHERNN
Gh_D01G1926        --NRQVARDGAGLRR-----SSSGSAGQMEEC---PQCRVKFSSVTALVEHVKKVHERNN
Gh_A09G1153        SQKLQIARDGVGLSSSSGSGSSNGNAGQVEEC---PQCGAKFASVTTLVDHVEKVHERNN
Gh_D09G1159        SQKLQIARDGVGLSSSG--GGSNGNAGQVEEC---PQCGAKFSSVTTLVDHVEKVHERNN
Gh_D11G2046        ------RRERKLL--NQLENKGVIKSIDPYVC---KVCGRRFYTNEKLVNHFKQIHEREH
Gh_A11G1928        ------RRERKLL--NQLENKGVIKSIDPYVC---KVCGRRFYTNEKLVNHFKQIHEREH
Gh_A06G0175        ------RRERRSL--DILERKGIVTPDEPYIC---GVCGRKCKTNLDLKKHFKQLHERER
Gh_D06G0169        ------RRERRSL--DILERKGIVKPDEPYIC---GVCGRKCKTNLDLKKHFKQLHERER
Gh_D12G1804        ------------------------------------------------------------
Gh_Sca005786G01    ------------------------------------------------------------
Gh_A03G0820        ------------------------------------------------------------
Gh_D02G2408        ------------------------------------------------------------
Gh_D07G1883        PLRCPCGVVLEKE--DMVQHQTSDCPLRLITC---RFCGDMVQAGSSA------------
Gh_A07G1674        PLRCPCGVVLEKE--DMVQHQASDCPLRLIAC---RFCGDMVQAGSSA------------
Gh_D03G0179        PLKLELGNSA-----SLENNSENVGSVQKFIC---RFCGLKFDLLPDLGRHHQAAHMGPS
Gh_A02G1540        PPKLELGNSA-----SLENNSENVGSVQKFIC---RFCGLKFDLLPDLGRHHQAAHMGPS
Gh_D03G0180        PPKLEFGNS-------VENNSENVDSFQKFTC---KYCGLKFDLLPDLGRHHQAAHMGPS
Gh_A02G1539        PPKLQFGNS-------VENNSENVDSFQKFTC---KYCGLKFDLLPDLGRHHQAAHMGPS
Gh_D13G1910        ------------------------------------------------------------
Gh_A13G2297        ------------------------------------------------------------
Gh_A09G0878        ------------------------------------------------------------
Gh_D09G0901        ------------------------------------------------------------
Gh_D05G0849        ------------------------------------------------------------
Gh_A05G0702        ------------------------------------------------------------
Gh_D01G2032        ------------------------------------------------------------
Gh_D11G3254        ------------------------------------------------------------
Gh_A11G2870        ------------------------------------------------------------
Gh_D05G1175        --------------------------QKQYVCQ-EVGCGKVFKFSSKLKKHEDSHVKLDS
Gh_A05G3867        --------------------------QKQYVCQ-EVGCGKVFKFASKLRKHEDSHVKLDS
Gh_A05G2341        --------------------------QKQYVCQ-EVGCGKVFEFASKLKKHEDSHVKLDS
Gh_D05G2606        --------------------------QKQYVCQ-EVGCGKVFEFKSKLKKHEDSHVKLDS
Gh_A12G0454        --------------------------------------------AKHFKCHVCHK-KLST
Gh_D12G0457        --------------------------------------------AKHFKCHVCHK-KLST
Gh_A03G1535        ---------------------------RRYRLGSSVGDG-VILRDGKFECQFCHK-VFHE
Gh_D01G1781        ----------------------------QYVCHW-EGCGKKFLDSSKLKRHFLIH-T-GE
Gh_A01G1532        ----------------------------QYVCHW-EGCGKKFLDSSKLKRHFLIH-T-GE
Gh_A10G0992        -----------------------------------------LLDEANAKRQLLLS-H---
Gh_D10G2570        -----------------------------------------LLDEANAKRQLLLS-H---
Gh_A06G0337        --------------------------NISIKEL------------SDMTAIKA--EDILT
Gh_D06G0368        --------------------------NISIKEL------------SDMTAIKA--EDILT
Gh_D05G3801        --------------------------NISIKEL------------SDMTAIKA--EDILT
Gh_A05G1995        --------------------------NISIKEL------------SDMTAIKA--EDILT
Gh_D07G0542        ------------------------------------------------------------
Gh_A07G0477        ------------------------------------------------------------
Gh_A05G2985        ------------------------------------------------------------
Gh_D04G0776        ------------------------------------------------------------
Gh_D05G0663        ------------------------------------------------------------
Gh_A05G3915        ------------------------------------------------------------
Gh_D07G0016        ------------------------------------------------------------
Gh_A07G0010        ------------------------------------------------------------
Gh_D12G2446        ------------------------------------------------------------
Gh_A12G2312        ------------------------------------------------------------
Gh_D08G1069        ------------------------------------------------------------
Gh_A08G2455        ------------------------------------------------------------
Gh_D07G2319        --------------------------YAMYECL-VEGCDLKFKNYKGRQQHLVDKHKFPT
Gh_A07G2108        --------------------------YAMYECL-VEGCDLKFKNYKGRQQHLVDKHKFPT
Gh_A05G2549        --------------------------ERPYKCK-VEGCSLSFRFVSDFSRHRRKTG----
Gh_D05G2825        --------------------------ERPYKCK-VEGCGLSFRFVSDFSRHRRKTG----
Gh_A07G2281        --------------------------ARPYVCG-EEGCGQTFRFVSDFSRHKRKTG----
```

```
Gh_D07G0545          --------------------------ARPYVCG-EEGCGQTFRFVSDFSRHKRKTG----
Gh_D09G2376          --------------------------ARPYVCA-EEGCGQTFRFVSDFSRHKRKTG----
Gh_A09G2168          --------------------------ARPYVCA-EEGCGQTFRFVSDFSRHKRKTG----


Gh_A06G0249          ------------------------------------------------------------
Gh_D02G2008          ------------------------------------------------------------
Gh_A03G1559          ------------------------------------------------------------
Gh_A01G1676          QSRVLKM--SIDV--------------------------CPKCGRGFRDPVSL-----VE
Gh_D01G1926          QSRVLKM--SIDV--------------------------CPKCGRGFRDPVSL-----VE
Gh_A09G1153          QSRVFKM--SIDV--------------------------CPKCSKGFRDPVAL-----VE
Gh_D09G1159          QSRVFKM--SIDV--------------------------CPKCSKGFRDPVAL-----VE
Gh_D11G2046          QKRVNQI--ESARGSRRVKLVGKYSMKMEKYKNAAREVLTPKVGYGLADELKRAGFW-VG
Gh_A11G1928          QKRVNQI--ESARGSRRVKLVGKYSMKMEKYKNAAREVLTPKVGYGLADELKRAGFW-VG
Gh_A06G0175          QKKLNRM--KSLKGKKRQRFKERFISGNHKYNEAARSIIKPKIGYGLASELRRAGVY-VK
Gh_D06G0169          QKKLNRM--KSLKGKKRQRFKERFISGNHKYNEAARSIIKPKIGYGLASELRRAGVY-VK
Gh_D12G1804          ------------------------------------------------------------
Gh_Sca005786G01      ------------------------------------------------------------
Gh_A03G0820          ------------------------------------------------------------
Gh_D02G2408          ------------------------------------------------------------
Gh_D07G1883          -MDVR----DRLRGLS-----------EHE--SICGSRTAPCDSCGR------------S
Gh_A07G1674          -MDVR----DRLRGLS-----------EHE--SICGSRTAPCDSCGR------------S
Gh_D03G0179          LASSR----PPKKGVR-----------YYAYKLKSGRLSHPRFKKGLGAVSYRIRNRATA
Gh_A02G1540          LASSR----PPKKGVR-----------YYAYKLKSGRLSHPRFKKGLGAVSYRIRNRATA
Gh_D03G0180          LASSR----PPKKGVR-----------YYAYKLKSGRLSHPRFKKGLGAVSYRIRNRATA
Gh_A02G1539          LASSR----PPKKGVR-----------YYAYKLKSGRLSHPRFKKGLGAVSYRIRNRATA
Gh_D13G1910          ------------------------------------------------------------
Gh_A13G2297          ------------------------------------------------------------
Gh_A09G0878          ------------------------------------------------------------
Gh_D09G0901          ------------------------------------------------------------
Gh_D05G0849          ------------------------------------------------------------
Gh_A05G0702          ------------------------------------------------------------
Gh_D01G2032          ------------------------------------------------------------
Gh_D11G3254          ------------------------------------------------------------
Gh_A11G2870          ------------------------------------------------------------
Gh_D05G1175          VEAFCSEPSCLKYF-TNEQ-------CLKA-HVQSCHQYINCQICGAKQLKKNIKRHLRS
Gh_A05G3867          VEAFCSEPSCMKYF-TNEQ-------CLKA-HVQSCHQYINCQICGAKQLKKNIKRHLRS
Gh_A05G2341          LEAFCSEPGCMKYF-TNEQ-------CLKA-HVQSSHAYINCQICGAKQLKKNIKRHLRS
Gh_D05G2606          VEAFCSEPGCMKYF-TNEQ-------CLKA-HVLSSHAYINCQICGAKQLKKNIKRHLRS
Gh_A12G0454          AGG----MAIHVLQVHKES-------VTKVPNAKTGRESTDIEIYGMQGIP---------
Gh_D12G0457          AGG----MAIHVLQVHKES-------VTKVPNAKPGRESTDIEIYGMQGIP---------
Gh_A03G1535          RRRYNGHVGIHVRNFVR----------------------------GIED-S---------
Gh_D01G1781          RDFICPHEGCGKA-----------------------------------------------
Gh_A01G1532          RDFICPHEGCGKA-----------------------------------------------
Gh_A10G0992          -MVVAPQSGVSANHLIQ----------------------------NHPP-P---------
Gh_D10G2570          -MVVAPQSGVSANHLIQ----------------------------NHPP-P---------
Gh_A06G0337          TLQSLELIQYRKGQHVICA-------DPKVLDRH-----------------------LKA
Gh_D06G0368          TLQSLELIQYRKGQHVICA-------DPKVLDRH-----------------------LKA
Gh_D05G3801          TLQSLELIQYRKGQHVICA-------DPKVLDRH-----------------------LKA
Gh_A05G1995          TLQSLELIQYRKGQHVICA-------DPKVLDRH-----------------------LKA
Gh_D07G0542          ------------------------------------------------------------
Gh_A07G0477          ------------------------------------------------------------
Gh_A05G2985          ------------------------------------------------------------
Gh_D04G0776          ------------------------------------------------------------
Gh_D05G0663          ------------------------------------------------------------
Gh_A05G3915          ------------------------------------------------------------
Gh_D07G0016          ------------------------------------------------------------
Gh_A07G0010          ------------------------------------------------------------
Gh_D12G2446          ------------------------------------------------------------
Gh_A12G2312          ------------------------------------------------------------
Gh_D08G1069          ------------------------------------------------------------
Gh_A08G2455          ------------------------------------------------------------
Gh_D07G2319          SFEFFKKAHPSKKQRLKNQ-------RKRAVNKKD------------EEVP--SKMEVEN
```

```
Gh_A07G2108        SFEFFKKAHPSKKQRLKNQ-------QKRAVNKKD------------EEAS--SKMEVEN
Gh_A05G2549        --------HYIDS-----------------------------------------------
Gh_D05G2825        --------HYIDS-----------------------------------------------
Gh_A07G2281        --------HSGKKGS---------------------------------------------
Gh_D07G0545        --------HCGKKGR---------------------------------------------
Gh_D09G2376        --------HSVKKGK-IKH-------ESRKL-----------------------------
Gh_A09G2168        --------HSLKKGKEIKH-------ESRKL-----------------------------


Gh_A06G0249        ------------------------------------------------------------
Gh_D02G2008        ------------------------------------------------------------
Gh_A03G1559        ------------------------------------------------------------
Gh_A01G1676        HVEK---------DHGGTSK--A-------------------------------------
Gh_D01G1926        HVEK---------DHGGTSK--A-------------------------------------
Gh_A09G1153        HVER---------DHGGTSK--A-------------------------------------
Gh_D09G1159        HVER---------DHGGTSK--A-------------------------------------
Gh_D11G2046        TVSN---------RPQAA---DVA----L--RDHMVDVMDKRKAECL------M----LV
Gh_A11G1928        TVSN---------RPQAA---DFA----L--RDHIVDVMDKRKAECL------M----LV
Gh_A06G0175        TVED---------KPQAA---DWA----L--KRQMQHSMS-RGIDWL------F----LV
Gh_D06G0169        TVED---------KPQAA---DWA----L--KRQMQHSMS-RGIDWL------F----LV
Gh_D12G1804        ------------------------------------------------------------
Gh_Sca005786G01    ------------------------------------------------------------
Gh_A03G0820        ------------------------------------------------------------
Gh_D02G2408        ------------------------------------------------------------
Gh_D07G1883        VMLK---------D--------MD----I--HQIAVHQ---KN-----------------
Gh_A07G1674        VMLK---------D--------MD----I--HQIAVHQ---KN-----------------
Gh_D03G0179        TMKK---------RLQASKLIDAE----I--ISAEPHVMENSNLGRLAEPQCSALAKILF
Gh_A02G1540        TMKK---------RLQASKLIDAE----I--ISAEPHVMENSNLGRLAEPQCSALAKILF
Gh_D03G0180        TMKK---------RLQASKLIDAE----I--ISAEPHVMETSNLGRLAEPQCSALAKILF
Gh_A02G1539        TMKK---------RLQASKLIDAE----V--ISAEPHVMETSNLVRLAEPQCSALAKILF
Gh_D13G1910        ------------------------------------------------------------
Gh_A13G2297        ------------------------------------------------------------
Gh_A09G0878        ------------------------------------------------------------
Gh_D09G0901        ------------------------------------------------------------
Gh_D05G0849        ------------------------------------------------------------
Gh_A05G0702        ------------------------------------------------------------
Gh_D01G2032        ------------------------------------------------------------
Gh_D11G3254        ------------------------------------------------------------
Gh_A11G2870        ------------------------------------------------------------
Gh_D05G1175        HES-GVASERIKCDFGGC----------------LHTFSTKSNLRQH----VKAVHE---
Gh_A05G3867        HES-GVASERIKCDFGGC----------------LHTFSTKSNLRQH----VKAVHE---
Gh_A05G2341        HEPGGVESERIKCDFEGC----------------LHTFSTKSNLRQH----IKAVHE---
Gh_D05G2606        HEPGGVESERIKCNFEGC----------------LHTFSNKSNLSLH----VKAVHE---
Gh_A12G0454        -------PDVLAAHYGE---EEEEAPSKAAKL--------------------DIPSTQLV
Gh_D12G0457        -------PDVLAAHYGE---EEEEAPSKAAKL--------------------DIPSTQLV
Gh_A03G1535        -------PGLLTLPRRTEVATKQELPARISKMDALIEIAQNSILETS----TAVPRYELN
Gh_D01G1781        ----------FSLDF---------------------------NLRSH----MKTH-----
Gh_A01G1532        ----------FSLDF---------------------------NLRSH----MKTH-----
Gh_A10G0992        -------LPALQQDS---------------------------QQQPR----LVVP-----
Gh_D10G2570        -------PPALQQDS---------------------------QQQPR----LVVP-----
Gh_A06G0337        AGRGGLEVDVSK------------------------------------------------
Gh_D06G0368        AGRGGLEVDVSK------------------------------------------------
Gh_D05G3801        AGRGGLEVDVSK------------------------------------------------
Gh_A05G1995        AGRGGLEVDVSK------------------------------------------------
Gh_D07G0542        ------------------------------------------------------------
Gh_A07G0477        ------------------------------------------------------------
Gh_A05G2985        ------------------------------------------------------------
Gh_D04G0776        ------------------------------------------------------------
Gh_D05G0663        ------------------------------------------------------------
Gh_A05G3915        ------------------------------------------------------------
Gh_D07G0016        ------------------------------------------------------------
Gh_A07G0010        ------------------------------------------------------------
Gh_D12G2446        ------------------------------------------------------------
```

```
Gh_A12G2312          ------------------------------------------------------------
Gh_D08G1069          ------------------------------------------------------------
Gh_A08G2455          ------------------------------------------------------------
Gh_D07G2319          EMMEGLVSAVSKLST-------------------------------S----DSSPSTVSF
Gh_A07G2108          EMMEGLVSAVSKLST-------------------------------S----DSSPSTVSF
Gh_A05G2549          ------------------------------------------------------------
Gh_D05G2825          ------------------------------------------------------------
Gh_A07G2281          ------------------------------------------------------------
Gh_D07G0545          ------------------------------------------------------------
Gh_D09G2376          ------------------------------------------------------------
Gh_A09G2168          ------------------------------------------------------------

Gh_A06G0249          ------------------------------------------------------------
Gh_D02G2008          ------------------------------------------------------------
Gh_A03G1559          ------------------------------------------------------------
Gh_A01G1676          ------------------------------------------------------------
Gh_D01G1926          ------------------------------------------------------------
Gh_A09G1153          ------------------------------------------------------------
Gh_D09G1159          ------------------------------------------------------------
Gh_D11G2046          S------DDSDFVGVLKEAKLRCLKTVVVGDADD----------------GALKRVADAG
Gh_A11G1928          S------DDSDFVGVLKEAKLRCLKTVVVGDADD----------------GALKRVADAG
Gh_A06G0175          S------DDKDFVEMLRRAREADLGTVVVGDWDR----------------G-LGRHADLW
Gh_D06G0169          S------DDKDFVEMLRRAREADLGTVVVGDWDR----------------G-LGRHADLW
Gh_D12G1804          ------------------------------------------------------------
Gh_Sca005786G01      ------------------------------------------------------------
Gh_A03G0820          ------------------------------------------------------------
Gh_D02G2408          ------------------------------------------------------------
Gh_D07G1883          ------------------------------------------------------------
Gh_A07G1674          ------------------------------------------------------------
Gh_D03G0179          SRTHKTKPRPNNLDILSIARSSCCKVSLKASLEEKYG---MLPECLYLKAAKLCSEHNVQ
Gh_A02G1540          SRTHKTKPRPNNLDILSIARSSCCKVSLKASLEEKYS---MLPECLYLKAAKLCSEHNVQ
Gh_D03G0180          SRTHKTKPRPNNLDILSIARSSCCKVSLKASLEEKYG---MLPECLYLKAAKLCSEHNVQ
Gh_A02G1539          SRTHKTKPQPNNLDILSIARFSCCKVSLKASLEGKYG---MLPECLYLKAAKLCSEHNIQ
Gh_D13G1910          ------------------------------------------------------------
Gh_A13G2297          ------------------------------------------------------------
Gh_A09G0878          ------------------------------------------------------------
Gh_D09G0901          ------------------------------------------------------------
Gh_D05G0849          ------------------------------------------------------------
Gh_A05G0702          ------------------------------------------------------------
Gh_D01G2032          ------------------------------------------------------------
Gh_D11G3254          ------------------------------------------------------------
Gh_A11G2870          ------------------------------------------------------------
Gh_D05G1175          ---------------------------------ELKPFACSFSGCGMRFS-------YKH
Gh_A05G3867          ---------------------------------ELKPFACSFSGCGMRFS-------YKH
Gh_A05G2341          ---------------------------------ELKPFACSFFGCGMRFS-------YKH
Gh_D05G2606          ---------------------------------ELKPFACSFFGCGMRFS-------YKH
Gh_A12G0454          GGL-----MPGPLGVGYPPQST------LGAVQPMYNAAVPVPPVGWAVP-------PRP
Gh_D12G0457          GGL-----MPGPLGVGYPPQST------LGAVQPMYNAAVPIPPVGWAVP-------PRP
Gh_A03G1535          DGS-----------------------------SPDKLNAVPIPDIPASTS-------D--
Gh_D01G1781          --------------------------------SQENYHICPYPDCGKRYA-------H--
Gh_A01G1532          --------------------------------SQENYHICPYPDCGKRYA-------H--
Gh_A10G0992          --------------------------------RQRFVPILPYPPLPSAI--------H--
Gh_D10G2570          --------------------------------RQRFVRILPYPPLPSAI--------H--
Gh_A06G0337          --------------LIWTPYKE------QS------------------------------
Gh_D06G0368          --------------LIWTPYKE------QS------------------------------
Gh_D05G3801          --------------LIWTPYKE------QS------------------------------
Gh_A05G1995          --------------LIWTPYKE------QS------------------------------
Gh_D07G0542          ------------------------------------------------------------
Gh_A07G0477          ------------------------------------------------------------
Gh_A05G2985          ------------------------------------------------------------
Gh_D04G0776          ------------------------------------------------------------
Gh_D05G0663          ------------------------------------------------------------
```

```
Gh_A05G3915          ------------------------------------------------------------
Gh_D07G0016          ------------------------------------------------------------
Gh_A07G0010          ------------------------------------------------------------
Gh_D12G2446          ------------------------------------------------------------
Gh_A12G2312          ------------------------------------------------------------
Gh_D08G1069          ------------------------------------------------------------
Gh_A08G2455          ------------------------------------------------------------
Gh_D07G2319          GR-------RNARGLTFVPRSI------QPPLQKK-------------------------
Gh_A07G2108          GR-------HNARGLTFVPRSV------QPPLQKK-------------------------
Gh_A05G2549          ------------------------------------------------------------
Gh_D05G2825          ------------------------------------------------------------
Gh_A07G2281          ------------------------------------------------------------
Gh_D07G0545          ------------------------------------------------------------
Gh_D09G2376          ------------------------------------------------------------
Gh_A09G2168          ------------------------------------------------------------


Gh_A06G0249          ------------------------------------------------------------
Gh_D02G2008          ------------------------------------------------------------
Gh_A03G1559          ------------------------------------------------------------
Gh_A01G1676          ------------------------------------------------------------
Gh_D01G1926          ------------------------------------------------------------
Gh_A09G1153          ------------------------------------------------------------
Gh_D09G1159          ------------------------------------------------------------
Gh_D11G2046          FSWTEI-----------LKGKAKKEAVSVVGKWK--DRDI--------------------
Gh_A11G1928          FSWTEI-----------LKGKAKKEAVSVVGKWK--DRDI--------------------
Gh_A06G0175          VSWVEV-----------ENGEVLEKDLVPKRKRMSSDDGL--------------------
Gh_D06G0169          VSWVEV-----------ENGEVSEKDLVPKRKRMSSDDGL--------------------
Gh_D12G1804          ------------------------------------------------------------
Gh_Sca005786G01      ------------------------------------------------------------
Gh_A03G0820          ------------------------------------------------------------
Gh_D02G2408          ------------------------------------------------------------
Gh_D07G1883          ------------------------------------------------------------
Gh_A07G1674          ------------------------------------------------------------
Gh_D03G0179          VEWHQE------------------KFVCINGCKPAKDPDF--------------------
Gh_A02G1540          VEWHQE------------------KFVCINGCKPAKDPDF--------------------
Gh_D03G0180          VEWHQE------------------KFVCINGCKPAKDSDF--------------------
Gh_A02G1539          VEWHQE------------------KFVCINGCKPAKDSDF--------------------
Gh_D13G1910          ------------------------------------------------------------
Gh_A13G2297          ------------------------------------------------------------
Gh_A09G0878          ------------------------------------------------------------
Gh_D09G0901          ------------------------------------------------------------
Gh_D05G0849          ------------------------------------------------------------
Gh_A05G0702          ------------------------------------------------------------
Gh_D01G2032          ------------------------------------------------------------
Gh_D11G3254          ------------------------------------------------------------
Gh_A11G2870          ------------------------------------------------------------
Gh_D05G1175          V--RDNHEKSALHVYVPGDFIESDEQFQ---SRPR-----------------GGRKRTCP
Gh_A05G3867          V--RDNHEKLALHVYVPGDFIESDEQFQ---SRPR-----------------GGRKRTCP
Gh_A05G2341          V--RDNHEKSGCHVYTPGNFLETDEQFC---SKPR-----------------GGRKRTCP
Gh_D05G2606          V--RDNHEKSGCHVYTPGNFLETDEQFR---SKPR-----------------GGLKRTCP
Gh_A12G0454          QPWLPLHPAASVPLSAPMGYVQQP-LFPVQGVRPPLAVPSTSPSLQPSQIAPPGLPVSTP
Gh_D12G0457          QPWLPQHPAASVPLSAPMGYVQQP-LFPVQGVRPPLAVPSTSPSLQPSQIAPPGLPVSTP
Gh_A03G1535          -----------------------------------------------------HEMNS--
Gh_D01G1781          -EYK---------LK--------------------------------NHIASHHEKNTTP
Gh_A01G1532          -EYK---------LK--------------------------------NHIASHHEKNTTP
Gh_A10G0992          -PFL---------IE--------------------------------AG--APQQWKIRP
Gh_D10G2570          -PFL---------IG--------------------------------AG--APQQWTIRP
Gh_A06G0337          ------------------------------------------------------------
Gh_D06G0368          ------------------------------------------------------------
Gh_D05G3801          ------------------------------------------------------------
Gh_A05G1995          ------------------------------------------------------------
Gh_D07G0542          ------------------------------------------------------------
```

```
Gh_A07G0477          ------------------------------------------------------------
Gh_A05G2985          ------------------------------------------------------------
Gh_D04G0776          ------------------------------------------------------------
Gh_D05G0663          ------------------------------------------------------------
Gh_A05G3915          ------------------------------------------------------------
Gh_D07G0016          ------------------------------------------------------------
Gh_A07G0010          ------------------------------------------------------------
Gh_D12G2446          ------------------------------------------------------------
Gh_A12G2312          ------------------------------------------------------------
Gh_D08G1069          ------------------------------------------------------------
Gh_A08G2455          ------------------------------------------------------------
Gh_D07G2319          ------------------------------------------------------------
Gh_A07G2108          ------------------------------------------------------------
Gh_A05G2549          ------------------------------------------------------------
Gh_D05G2825          ------------------------------------------------------------
Gh_A07G2281          ------------------------------------------------------------
Gh_D07G0545          ------------------------------------------------------------
Gh_D09G2376          ------------------------------------------------------------
Gh_A09G2168          ------------------------------------------------------------
```

Subgroup 7

```
Gh_A07G1366          HAHNCSQHTPSNGAAS-----AIPNSVLDPNGAAAAMLPA--------------------
Gh_D07G2456          HGHNGSQHTPSNGAAS-----AIPNSVLDPNGAAAAMLPS--------------------
Gh_D07G0695          AAVNSVRMCTICNVACNSH-EVFVKHLSGRRHAAQAGL----------------------
Gh_D12G1787          ------FHSGVNNVACNSH-DAFVKHLSGRRHVAQVRL----------------------
Gh_D12G1778          --------------NPNFSPRR----------------SE--------------------
Gh_A09G1165          --------TASELASVLSSSSSFSEMRD--K-------DG--------------------
Gh_D09G1170          --------TASELASVLSSSSSFSEMRD--K-------DG--------------------
Gh_A02G1056          --------SASELASVLSSSSEITDKDGDGD-------DS--------------------
Gh_D03G0634          --------SASELASFLSSSSEITDKDGDGD-------DS--------------------
Gh_D01G1184          --------SASELASVLSASSEMTEQDD--------------------------------
Gh_A01G1108          --------SASELASVLSASSVMTEQDD--------------------------------
Gh_D08G0325          --------SSDLKSRASSPSKDMTADKLQRN--PVSAGAA--------------------
Gh_A08G0236          --------SSDLKSRASSPSKDLTADKLQWN--PVTAGAA--------------------
Gh_A13G2132          --------VETSSSNSND------------------------------------------
Gh_D13G0718          --------VETSSSNSND------------------------------------------
Gh_D06G0302          --------SNGSNTNSSK----------------ENI-----------------------
Gh_A06G0282          --------SNDSNTNSSK----------------ENI-----------------------
Gh_A11G1476          --------SSDYGFSPSVDL---TDNCGVNG----NV-AA--------------------
Gh_D11G1633          --------SSDYGFSPSVDL---TDNCGVNG----NV-AA--------------------
Gh_D08G0579          --------ISDLGFGGLSPSAHVTDRLGGN--------GS--------------------
Gh_A08G0493          --------ISDLGFGGLSPSAHVTDRLGGN--------GS--------------------
Gh_D13G2257          --------SSCSNDLMMVSSRFSPQEVTDDK-EDHTIVAS--------------------
Gh_A13G1869          --------SSCSNDLMMVSSRFSPQEVTDDK-EDHTIVAS--------------------
Gh_A09G0899          --------STGDDGDNNNNYSVAENKVFKEE--EEPMKKS--------------------
Gh_D09G0924          --------STGDDGDNNNNYSVAENKVFKEE--EEPMKKS--------------------
Gh_A07G0996          ------------------------------------------------------------
Gh_D07G1074          --------SAAAAAEGDDNIPVVDHKILKEE---EPM-KK--------------------
Gh_D11G0559          --------------------PVL--------------NPT--------------------
Gh_A11G0481          --------------------PVL--------------NPT--------------------
Gh_D11G0560          --------------------RNS--------------NPT--------------------
Gh_A11G0482          --------------------RNS--------------NPT--------------------
Gh_A02G0617          V-------SSNSGE-QQNH--VMEEHEM---------KDE--------------------
Gh_D02G0671          V-------SSNSGE-QQNH--VMEEHEM---------KDE--------------------
Gh_A09G1098          I-------TSNNGNEQNNH--IVEEHEL---------KDE--------------------
Gh_D09G1104          I-------TSNNGNEQSNH--IIEEHEL---------KDE--------------------
Gh_A09G0835          --------------HNNQSLQVGNADKLKERKELGEMVQR--------------------
Gh_D09G0856          --------------HNNRSLQVGNADKLKERKELGEMVQR--------------------
Gh_D05G0854          H-------NNNNGNNRNRSLQVSNDDKL-ERKEFVELPRR--------------------
Gh_A05G0718          R-------NNTNGNNRNRSLQVSNDDKL-ERKEFVEFPRR--------------------
Gh_D12G1536          -------DLLSLTPLD--------NGKRKRK---PPGTP---------------------
Gh_A05G2664          -------SSDPLSPLEN---GVT--QKRKRK---PAGTP---------------------
```

```
Gh_D05G2965          -------SSDPLSPLEN---GDT--QKRKRK---PAGTP---------------------
Gh_D02G1382          -------PSDPLSSFQN---GVS--HKRKRK---PAGTP---------------------
Gh_A03G0994          -------PSDPLSSFQN---GVS--HKRKRK---PAGTP---------------------
Gh_D01G1332          -------SSDPLTPLEN---GAT--HKRKRK---PAGTP---------------------
Gh_Sca004794G01      -------SSDPLTPLEN---GAT--HKRKRK---PAGTP---------------------
Gh_A12G2459          -------SSKPIPCFEN---GDNNNNKRKRR---PAGTP---------------------
Gh_D12G2587          -------SSKPIPCFEN---GDNNNNKRKRR---PAGTP---------------------
Gh_A11G0018          -------LY-----LEN---GST-NNKRKRR---PAGTP---------------------
Gh_D11G0016          -------LY-----LEN---GST-NNKRKRR---PAGTP---------------------
Gh_A03G0782          -------SSEPFSCLEN---GTTNINKRKRR---PAGTP---------------------
Gh_D02G1197          -------SSEPFSCLEN---GTTNINKRKRR---PAGTP---------------------
Gh_D09G1639          -------SLEPFTCIDN---GNN-NNKRKRR---PAGTP---------------------
Gh_A09G1568          -------SLEPFTCIDN---GNN-NNKRKRR---PAGTP---------------------
Gh_D02G1400          ---QSSTATPTSSTAPT----TAPQK-RKRN---HPGTS---------------------
Gh_A12G1411          ---QSS-AAPTSSTAPT----TAPHSKKKRN---QPGTPSKYLF----------------
Gh_D12G1529          ------------------------------------------------------------
Gh_A12G1412          ---LQQHHTPTSSTGPV---DPSPPPKKKRN---QPGTPS--------------------
Gh_D12G1530          ---Q-QQHTPTSSTGPV---DPSPPPKKKRN---QPGTPSKYPI----------------
Gh_D01G1353          ---LQQHQL--QLWGQL---H--------RR---LRGKR---------------------
Gh_A01G2114          -----------MRFGFL---S--------EC-------P---------------------
Gh_A09G0997          -----------PNPNPT---LSVNPSKKKRN---LPGTPGKSVN----------------
Gh_D09G1017          ------------------------------------------------------------
Gh_D09G1416          -----------ANPNPK---PNANPAKKKRN---LPGTP---------------------
Gh_A09G1411          -----------ANPNPK---PNANPAKKKRN---LPGTP---------------------
Gh_A04G0633          --------------------------MADET---DFKDM---------------------
Gh_D04G1096          -----------NNTNPN---PKSNPVKKKRN---LPGNP---------------------
Gh_D02G1798          ----S--SNESPNPIEH---PITTSSKKKRN---LPGNPGKHAF----------------
Gh_A03G1358          ----S--SNESPNPIEH---PITTSSKKKRN---LPGNPGKHAF----------------
Gh_A12G0073          ---GASNTNGSSSSSSQ---QPLQGAKRKRN---LP------------------------
Gh_D12G0089          ---GASNTNGSSSSSSQ---QPLQGAKRKRN---LP------------------------
Gh_D08G0786          ---GSGPSAASNSNN-----GSQQAVKKKRN---LPGTPGNYIYNSFPKSI---------
Gh_A08G0678          ---GSGPSAASNSNN-----GSQQAVKKKRN---LPGTPGNYIYNSFPKSI---------
Gh_D11G1217          -------ALVSNTNS-----SSSQHP------------------QPQPPYI---------
Gh_A11G1062          -------ASVSNINS-----SSSQQP---QP-----------QPQPQPPYI---------
Gh_D02G1806          -----------NPVPGS---NNPPVAKRKRN---LPGTPGK-------------------
Gh_A03G1366          -----------DPVPGS---NNPPVAKRKRN---LPGTPGK-------------------
Gh_A12G1286          -----------NPIPGS---N-PPVAKKKRN---LPGTPGKCLLFPPISNFSGDSLIVIF
Gh_D12G1408          ------------------------------------------------------------
Gh_A10G0519          ---Q------YFSNP-P---AQAQPVKRKRN---QPGNP---------------------
Gh_D10G0557          ---Q------YFSNP-P---AQAQPVKRKRN---QPGNP---------------------
Gh_D05G1287          ---Q------YFTTSQQ---PQTQPVKRKRN---LPGNPGLVWVQKALYL-NTILFCYLF
Gh_A05G1117          ---Q------YFTTSQQ---PQTQPVKRKRN---LPGNPGLVWVQKALYL-NTILFCYLF
Gh_D11G1304          -------VVTHQ--------QLPQKMKKKRS---LPGNPGTTFVLSLPVF----LF----
Gh_A11G1150          -------IVTHQ--------QLPQKIKKKRS---LPGNPGTTFFLSLPVF----LF----
Gh_A12G0016          -------IVSTISPQQQ---PQ--NIKKKRS---LPGNP---------------------
Gh_D12G0018          -------IVSTISPQQQ---PQ--NIKKKRS---LPGNP---------------------
Gh_A08G0586          -------IVSTISPHQS---PLQKVVKKKRN---LPGNP---------------------
Gh_D08G0679          -------IVSTISPHQP---PQQNVVKKKRN---LPGNP---------------------
Gh_D11G2734          ---NS--LLSTNSNGGS---TAPPPAKKKRN---LPGTP---------------------
Gh_A11G2417          ---NS--LLSTNSNGGS---TAPPPAKKKRN---LPGTP---------------------
Gh_D06G0903          ---QS-------FASTN---QAPAAAKKKRN---LPGNP---------------------
Gh_A06G0780          ---QS-------FASTN---QAPAAAKKKRN---LPGNP---------------------
Gh_A05G0322          ----------------N---QE-PLSKKKRN---LPGNP---------------------
Gh_D05G0428          ----------------N---QE-PLSKKKRT---LPGNP---------------------
Gh_D11G1873          --------------------LPKSTAKKKRN---LPGMP---------------------
Gh_A11G1715          --------------------LPKSTAKKKRN---LPGMP---------------------
Gh_A02G1568          --------------------PPKSTVKKKRN---LPGMP---------------------
Gh_D03G0157          --------------------PPKSAVKKKRN---LPGMP---------------------
Gh_A02G1566          --------------------PPKSTVKKKRN---LPGMP---------------------
Gh_D03G0158          --------------------PPKSTVKKKRN---LPGMP---------------------
Gh_A06G0089          ---Q------------Q---TDGVQSKKKRN---LPGMP---------------------
Gh_D06G0062          ---Q------------Q---TDGVQSKKKRN---LPGMP---------------------
Gh_D05G1861          ------------------------------------------------------------
```

```
Gh_A05G1677           ---Q------------L---TGGGPPKKKRN---LPGMP---------------------


Gh_A07G1366           --------ASVPGHAT--------LPAQV-----------PAAPLRPP--PRMAWCELCR
Gh_D07G2456           --------ASVPGHAT--------LPAQV-----------PAAPLRPP--PRMAWCELCR
Gh_D07G0695           --------IAVDGVGP--------YLAAVRANDQF-WNKGKKTS--KV--VQSSWCEVCE
Gh_D12G1787           --------IAIDGIGP--------YLAAIQANDQF-WNKGKKITKNKI--SQPTWCEVCQ
Gh_D12G1778           -----------KEQLT--------SLNTL-NASQTKILQLESSSKNAPQQLIHFYCEVCQ
Gh_A09G1165           -------------DEY--------PVNTL-NKGQY-WIPTPSQILIGP---TQFSCPVCC
Gh_D09G1170           -------------DEY--------PVNTL-SKGQY-WIPTPSQILIGP---TQFSCPVCC
Gh_A02G1056           -------------SGC--------AVNRL-NKGQY-WIPTPSQILIGP---TQFSCPVCC
Gh_D03G0634           -------------SGC--------AVNRL-NKGQY-WIPTPSQILIGP---TQFSCPVCC
Gh_D01G1184           --------------EY--------PINRL-NKRQY-WIPTPSQILIGP---SQFSCPVCC
Gh_A01G1108           --------------EY--------PITRL-NKRQY-WIPTPSQILIGP---SQFSCPVCC
Gh_D08G0325           -------------SGN--------STTPL-SKGQY-WIPTPSQILVGP---TQFSCPLCC
Gh_A08G0236           -------------SGN--------STTPL-SKGQY-WIPTPSQILVGP---TQFSCPLCC
Gh_A13G2132           ------------------------IGNSL-VEGQY-WIPSPAQILVGP---TQFSCSVCN
Gh_D13G0718           ------------------------IGNSL-VEGQY-WIPSPAQILVGP---TQFSCSVCN
Gh_D06G0302           --------------------------ANG-GGKQF-WIPTPEQILIGF---TNFSCHVCF
Gh_A06G0282           --------------------------ANG-GGKQY-WIPTPEQILIGF---TNFSCHVCF
Gh_A11G1476           -------------SGY--------PLSCT-SKGQY-WIPTPTQILVGP---TQFSCPVCC
Gh_D11G1633           -------------SGY--------PLSCS-SKGQY-WIPTPTQILVGP---TQFSCPVCC
Gh_D08G0579           -------------A-V--------PGYPL-TKGQY-WIPTPTQILVGP---TQFSCHVCC
Gh_A08G0493           -------------A-V--------PGYPL-TKGQY-WIPTPTQILVGP---TQFSCHVCC
Gh_D13G2257           -------------EYL--------S-NTL-NKGQY-WIPTPSQILIGP---TQFSCPLCF
Gh_A13G1869           -------------EYL--------S-NTL-NKGQY-WIPTPSQILIGP---TQFSCPLCF
Gh_A09G0899           -------------CFH--------GCSFN-TESRF-WIPTPAQILVGP---MQFACSICS
Gh_D09G0924           -------------CFH--------GCSFN-TESRF-WIPTPAQILVGP---MQFACSICS
Gh_A07G0996           ------------------------------------------------------------
Gh_D07G1074           -------------TFH--------GCSFN-TMSRF-WIPTPAQILVGP---MQFACSICS
Gh_D11G0559           -----------PETKP--------EFEDY-NGGESEVIELDAVELLSE---HLHFCEICG
Gh_A11G0481           -----------PETKP--------EFEDY-DGGESEVIELDAVELLSE---HLHFCEICG
Gh_D11G0560           -----------PELKP--------EFEDS-DEGEPDVIEVDAVELLSE---HLHFCEICR
Gh_A11G0482           -----------PELKP--------EFEDS-DKGESNVIEVDAVELLSE---HLHFCEICG
Gh_A02G0617           -----------EDAEE--------LGENL-FPGTYEILQLEKEEILAP---HTHFCTICG
Gh_D02G0671           -----------EDAEE--------LGENL-FPGTYEILQLEKEEILAP---HTHFCTICG
Gh_A09G1098           -----------EDAE---------EGENL-LPGTYEILQLEKEEILAP---HTHFCTICG
Gh_D09G1104           -----------EDAE---------EGENL-LPGTSEILQLEKEEILAA---HTHFCTICG
Gh_A09G0835           -----------SEISE--------GSQGD-ASTNCDIVELDAEDLLAK---YTHYCQVCG
Gh_D09G0856           -----------SEISE--------GSQGD-ASTNCDIVELDAEDLLAK---YTHYCQVCG
Gh_D05G0854           -----------SEISG--------GCEAD-SAMNYDIIELDAEDLLAK---YTHYCQVCG
Gh_A05G0718           -----------SEISG--------GCEAV-PAMNYDIIELDAEDLLAK---YTHYCQVCG
Gh_D12G1536           -------------------------------DPEAEVVSLSPQTLLES---DRYVCEICN
Gh_A05G2664           -------------------------------DPDAEVVSLSPKTLLES---DRYVCEICS
Gh_D05G2965           -------------------------------DPDAEVVSLSPKTLLES---DRYVCEICS
Gh_D02G1382           -------------------------------DPDAEVVSLSPKTLLES---DRYVCEICN
Gh_A03G0994           -------------------------------DPDAEVVSLSPKTLLES---DRYVCEICN
Gh_D01G1332           -------------------------------DPDAEVVSLSPKTLLES---DRYVCEICN
Gh_Sca004794G01       -------------------------------DPNAEVVSLSPKTLLES---DRYVCEICN
Gh_A12G2459           -------------------------------DPDAEVVSLSPKTLLES---DRYVCEICN
Gh_D12G2587           -------------------------------DPDAEVVSLSPKTLLES---DRYVCEICN
Gh_A11G0018           -------------------------------DPDAEVVSLSPKTLLES---DRYVCEICN
Gh_D11G0016           -------------------------------DPDAEVVSLSPKTLLES---DRYVCEICN
Gh_A03G0782           -------------------------------DPDAEVVSLSPKTLLES---DRYVCEICN
Gh_D02G1197           -------------------------------DPDAEVVSLSPKTLLES---DRYVCEICN
Gh_D09G1639           -------------------------------DPDAEVVSLSPKTLLES---DRYVCEICN
Gh_A09G1568           -------------------------------DPDAEVVSLSPKTLLES---DRYVCEICN
Gh_D02G1400           ---------------------------------------------------CKFICEVCN
Gh_A12G1411           ---------------------T-----YISLYPDVEVIALSPKSLMAT---NRFICEVCN
Gh_D12G1529           ---------------------------------------------MAT---NRFICEVCN
Gh_A12G1412           ----------------------------------------------------KFICEVCN
Gh_D12G1530           ---------------------FFFILFLLHIYPDAEVVALSPKTLMAT---NRFICEVCN
Gh_D01G1353           -----------------------ETNQSYQANPDVEVIALSPKSLMAT---NRFICEVCN
```

```
Gh_A01G2114      -----------------------MGERVMVANPDVEVIALSPKSLMAT---NRFICEVCN
Gh_A09G0997      ---------------------------FHSSDPDAEVIALSPKSLMAT---NRFLCEICN
Gh_D09G1017      ---------------------------------------------MAT---NRFLCEICN
Gh_D09G1416      -------------------------------DPDAEVIALSPKTLMAT---NRFICEICK
Gh_A09G1411      -------------------------------DPDAEVIALSPKTLMAT---NRFICEICK
Gh_A04G0633      -------------------------------DPDAEVIALSPKTLMAT---NRFICEICN
Gh_D04G1096      -------------------------------DPDAEVIALSPKTLMAT---NRFICEICN
Gh_D02G1798      -------------------------FFGKFSDPDAEVVALSPRTLMAT---NRYICEVCH
Gh_A03G1358      -------------------------FFGKFSDPDAEVVALSPRTLMAT---NRYICEVCH
Gh_A12G0073      ----------------------------GTPDPNAEVIALSPTTLMAT---NRFVCEICK
Gh_D12G0089      ----------------------------GTPDPNAEVIALSPTTLMAK---NRFVCEICK
Gh_D08G0786      -----------------------LIMLSSIINPNAEVIALSPTTLMAT---NRFVCEICN
Gh_A08G0678      -----------------------LIMLSSIINPNAEVIALSPTTLMAT---NRFVCEICN
Gh_D11G1217      -----------------------IVVLSMKPDPNAEVIALSPTTLMAT---NRFVCEICN
Gh_A11G1062      -----------------------IVVLSMKPDPNAEVIALSPTTLMAT---NRFVCEICN
Gh_D02G1806      -----------------------------------------------------FLCEICG
Gh_A03G1366      -----------------------------------------------------FLCEICG
Gh_A12G1286      VNL------------MTKCCLRFPHMIWVIADPEAEVIALSPKTLMAT---NRFLCEICG
Gh_D12G1408      -------------------------MIWVIADPEAEVIALSPKTLMAT---NRFLCEICG
Gh_A10G0519      -------------------------------DPDAEVIALSPKTLMAT---NRFVCEICN
Gh_D10G0557      -------------------------------DPDAEVIALSPKTLMTT---NRFVCEICN
Gh_D05G1287      CSFPFLLSLSLSGTQFLMHFLGGFFIFYFESYPDAEVIALSPKTLMAT---NRFVCEICN
Gh_A05G1117      CSFPYLLSLSLSETQFLMHFLGGFFVFYFESDPDAEVIALSPKTLMAT---NRFVCEICN
Gh_D11G1304      ----------------RL-HVLVFNIFYSITDPDAEVIALSPKTLLAT---NRFICEICN
Gh_A11G1150      ----------------RL-HVLVFNSFYSITDPDAEVIALSPKTLLAT---NRFICEICN
Gh_A12G0016      -------------------------------DPDAEVIALSPKTLLAT---NRFVCEICN
Gh_D12G0018      -------------------------------DPDAEVIALSPRTLLAT---NRFVCEICN
Gh_A08G0586      -------------------------------DPDSEVIALSPKTLLST---NRFVCEVCN
Gh_D08G0679      -------------------------------DPDSEVIALSPKTLLST---NRFVCEVCN
Gh_D11G2734      -------------------------------DPNAEVIALSPKTLLAT---NRFVCEICN
Gh_A11G2417      -------------------------------DPNAEVIALSPKTLLAT---NRFVCEICN
Gh_D06G0903      -------------------------------DPDAEVIALSPKTLLAT---NRFMCEICN
Gh_A06G0780      -------------------------------DPDAEVIALSPKTLLAT---NRFMCEICN
Gh_A05G0322      -------------------------------DPETEVIALSPKTLLAT---NRFICEICN
Gh_D05G0428      -------------------------------DPEAEVIALSPKTLLAT---NRFICEICN
Gh_D11G1873      -------------------------------DPDAEVIALSPRTLLAT---NRFVCEICN
Gh_A11G1715      -------------------------------DPDAEVIALSPSTLLAT---NRFVCEICN
Gh_A02G1568      -------------------------------DPDAEVIALSPTTLLAT---NRFVCEICN
Gh_D03G0157      -------------------------------DPDAEVIALSPTTLLAT---NRFVCEICN
Gh_A02G1566      -------------------------------DPDAEVIALSPTTLLAT---NRFVCEICN
Gh_D03G0158      -------------------------------DPDAEVIALSPTTLLAT---NRFVCEICN
Gh_A06G0089      -------------------------------DPEAEVISLSPKSLLAT---NRFICEICN
Gh_D06G0062      -------------------------------DPEAEVISLSPKSLLAT---NRFICEICN
Gh_D05G1861      -----------------------------------------MSLKQXT---NRFVCEICN
Gh_A05G1677      -------------------------------DPDAEVIALSPKTLLAT---NRFVCEICN

Gh_A07G1366      VDCNRPEIL-EQHKNGKRH--KKNLQVREELQKRNGVITGQ-QSVQVPNLGSEIVQLVKV
Gh_D07G2456      VDCNRPEIL-EQHKNGKRH--KKNLQVREELQKRNGVITGQ-QSVQVPNLGSEIVQLEKV
Gh_D07G0695      INCNSGDAY-AQHLSGKKH--LKKLENLEKSKKGTSD-----PSMGAPAEMNQMIKPVEN
Gh_D12G1787      INCNSSNVY-AKHLSGKKH--LKNLQNLEKSKNSTCY---S-SSIDTPIAANLLIGAVEN
Gh_D12G1778      VPCSCSLNY-KNHLNEKHK--VKLQELKFGRKD-GD----EDCEMEN------PKPRCE-
Gh_A09G1165      KTFNRYNNMQQMHMWGHGSEYRKGPESLKGSQP-T----------------GMLRLPCY-
Gh_D09G1170      KTFNRYNNM-QMHMWGHGSEYRKGPESLKGSQP-T----------------GMLRLPCY-
Gh_A02G1056      KTFNRYNNM-QMHMWGHGSQYRKGPESLRGTQP-T----------------GMLRLPCY-
Gh_D03G0634      KTFNRYNNM-QMHMWGHGSQYRKGPESLRGTQP-T----------------GMLRLPCY-
Gh_D01G1184      KTFNRYNNM-QMHMWAHGSQYRKGTESLRGTQP-T----------------AMLRLPCY-
Gh_A01G1108      KTFNRYNNM-QMHMWAHGSQYRKGPESLRGTQP-T----------------AMLRLPCY-
Gh_D08G0325      KTFNRYNNL-QMHMWGHGSQYRKGPDSLKGAQP-T----------------AMLRLPCY-
Gh_A08G0236      KTFNRYNNL-QMHMWGHGSQYRKGPDSLKGTQP-T----------------AMLRLPCY-
Gh_A13G2132      KTFNRYNNM-QMHMWGHGSQYRKGPESLRGTKP-A-------------S--SMLRLPCY-
Gh_D13G0718      KTFNRYNNM-QMHMWGHGSQYRKGPESLRGTKP-A-------------S--SMLRLPCY-
Gh_D06G0302      KTFNRYNNL-QMHMWGHGSQYRKGPESLKGTQP-P----------------AMLGIPCY-
```

```
Gh_A06G0282        KTFNRYNNL-QMHMWGHGSQYRKGPESLKGTQP-R----------------AMLGIPCY-
Gh_A11G1476        KTFNRYNNL-QMHMWGHGSQYRKGPDSLRGSQP-T----------------GMLRLPCY-
Gh_D11G1633        KTFNRYNNL-QMHMWGHGSQYRKGPDSLRGSQP-T----------------GMLRLPCY-
Gh_D08G0579        KTFNRYNNL-QMHMWGHGSQYRKGPDSLRGSQP-T----------------AMLRLPCY-
Gh_A08G0493        KTFNRYNNL-QMHMWGHGSQYRKGPDSLRGSQP-T----------------AMLRLPCY-
Gh_D13G2257        KTFNRYNNM-QMHMWGHGSQYRKGPESLRGTQP-T----------------AMLRLPCY-
Gh_A13G1869        KTFNRYNNM-QMHMWGHGSQYRKGPESLRGTQP-T----------------AMLRLPCY-
Gh_A09G0899        KTFNRYNNM-QMHMWGHGSEYRKGPDSLKGTQP-T----------------AMLRLPCY-
Gh_D09G0924        KTFNRYNNM-QMHMWGHGSEYRKGPDSLKGTQP-T----------------AMLRLPCY-
Gh_A07G0996        -----------MHMWGHGSEYRKGPDSLKGSQP-A----------------AMLRLPCY-
Gh_D07G1074        KTFNRYNNM-QMHMWGHGSEYRKGPDSLKGSQP-A----------------AMLRLPCY-
Gh_D11G0559        KGFKRDANL-RMHMRAHGDQYKTPEALAKKPE-----V---------KN--TVRKAQFS-
Gh_A11G0481        KGFKRDANL-RMHMRAHGDQYKTPEALAKKPE-----V---------KN--TVRKTQFS-
Gh_D11G0560        KSFKRDANL-RMHMRAHGDQYKTPEALAKKSEN-NSDV----------MN--PGRKTRFS-
Gh_A11G0482        KSFKRDANL-RMHMRAHGDQYKTPEALAKISEN-NSDV----------KT--PGRKTRFS-
Gh_A02G0617        KGFKRDANL-RMHMRGHGDEYKTPAALAKPNKE-SS-----------SE--PALIKRYS-
Gh_D02G0671        KGFKRDANL-RMHMRGHGDEYKTPAALAKPNKE-SS-----------SE--PALIKRYS-
Gh_A09G1098        KGFKRDANL-RMHMRGHGDEYKTPGALAKPTKE-SS-----------SE--PTIIKRYS-
Gh_D09G1104        KGFKRDANL-RMHMRGHGDEYKTPGALAKPTKE-SS-----------SE--PTIIKRYS-
Gh_A09G0835        KGFKRDANL-RMHMRAHGDEYKTNAALVNPMKS-QE---GSNNGMGNCS--MKFPKRYS-
Gh_D09G0856        KGFKRDANL-RMHMRAHGDEYKTNAALVNPMKS-HE---GSSNGMGNCS--TKCPKRYS-
Gh_D05G0854        KGFKRDANL-RMHMRAHGDEYKSSAALINPMKN-PREGSSSSSAMGNCS--TKMNKKYS-
Gh_A05G0718        KGFKRDANL-RMHMRAHGDEYKSSAALINPMKN-PREGSSSSSALGNCS--TKMNKKYS-
Gh_D12G1536        QGFQRDQNL-QMHRRRHKVPWKLV--------KSET-----------Q----EVVKKKT-
Gh_A05G2664        QGFQRDQNL-QMHRRRHKVPWKLL--------KREN-----------Q----EVKKRVFV
Gh_D05G2965        QGFQRDQNL-QMHRRRHKVPWKLL--------KREN-----------Q----EVKKRVFV
Gh_D02G1382        QGFQRDQNL-QMHRRRHKVPWKLL--------KRET-----------Q----EVKKRVFV
Gh_A03G0994        QGFQRDQNL-QMHRRRHKVPWKLL--------KRET-----------Q----EVKKRVFV
Gh_D01G1332        QGFQRDQNL-QMHRRRHKVPWKLL--------KRES-----------Q----EVKKRVFV
Gh_Sca004794G01    QGFQRDQNL-QMHRRRHKVPWKLL--------KRES-----------Q----EVKKRVFV
Gh_A12G2459        QGFQRDQNL-QMHRRRHKVPWKLL--------KRET-----------P----AVKKRVFV
Gh_D12G2587        QGFQRDQNL-QMHRRRHKVPWKLL--------KRET-----------P----AVKKRVFV
Gh_A11G0018        QGFQRDQNL-QMHRRRHKVPWKLL--------KRET-----------P----VVRKRVFV
Gh_D11G0016        QGFQRDQNL-QMHRRRHKVPWKLL--------KRET-----------P----VVRKRVFV
Gh_A03G0782        QGFQRDQNL-QMHRRRHKVPWKLL--------KRET-----------P----VVRKRVFV
Gh_D02G1197        QGFQRDQNL-QMHRRRHKVPWKLL--------KRET-----------P----VVRKRVFV
Gh_D09G1639        QGFQRDQNL-QMHRRRHKVPWKLL--------KRET-----------P----VVRKRVFV
Gh_A09G1568        QGFQRDQNL-QMHRRRHKVPWKLL--------KRET-----------P----VVRKRVFV
Gh_D02G1400        KGFQREQNL-QLHRRGHNLPWKLR--------QKTT-----------K----EVKRKVYL
Gh_A12G1411        KGFQREQNL-QLHRRGHNLPWKLK--------QKST-----------K----EVKKKVYL
Gh_D12G1529        KGFQREQNL-QLHRRGHNLPWKLK--------QKST-----------K----EVKKKVYL
Gh_A12G1412        KGFQREQNL-QLHRRGHNLPWKLK--------QKTT-----------K----EVKKKVYL
Gh_D12G1530        KGFQREQNL-QLHRRGHNLPWKLK--------QKTT-----------K----EVKKKVYL
Gh_D01G1353        KGFQREQNL-QLHRRGHNLPWKLR--------QKTT-----------K----EVKRKVYL
Gh_A01G2114        KGFQREQNL-QLHRRGHNLPWKLR--------QKTT-----------K----EVKRKVYL
Gh_A09G0997        KGFQRDQNL-QLHRRGHNLPWKLK--------QRTN-----------K---E-VRKKVYI
Gh_D09G1017        KGFQRDQNL-QLHRRGHNLPWKLK--------QRTN-----------K---E-VRKKVYI
Gh_D09G1416        KGFQRDQNL-QLHRRGHNLPWKLR--------QRTN-----------K---E-VRKKVYI
Gh_A09G1411        KGFQRDQNL-QLHRRGHNLPWKLR--------QRTN-----------K---E-VRKKVYI
Gh_A04G0633        KGFQRDQNL-QLHRRGHNLPWKLK--------QRNN-----------K---EPVKKKVYI
Gh_D04G1096        KGFQRDQNL-QLHRRGHNLPWKLK--------QRNN-----------K---EPVKKKVYI
Gh_D02G1798        KGFQRDQNL-QLHRRGHNLPWKLK--------QRTN-----------T----EVKKRVYV
Gh_A03G1358        KGFQRDQNL-QLHRRGHNLPWKLK--------QRTN-----------T----EVKKRVYV
Gh_A12G0073        KGFQRDQNL-QLHRRGHNLPWKLK--------QRAT-----------T----EVKKRVYV
Gh_D12G0089        KGFQRDQNL-QLHRRGHNLPWKLK--------QRAT-----------T----EVKKRVYV
Gh_D08G0786        KGFQRDQNL-QLHRRGHNLPWKLK--------QRTT-----------T----EVKKRVYI
Gh_A08G0678        KGFQRDQNL-QLHRRGHNLPWKLK--------QRTT-----------T----EVKKRVYI
Gh_D11G1217        KGFQRDQNL-QLHRRGHNLPWKLR--------QRTT-----------T----EVKKRVYI
Gh_A11G1062        KGFQRDQNL-QLHRRGHNLPWKLR--------QRTT-----------T----EVKKRVYI
Gh_D02G1806        KGFQRDQNL-QLHRRGHNLPWKLK--------QRTT-----------K----EVRKRVYV
Gh_A03G1366        KGFQRDQNL-QLHRRGHNLPWKLK--------QRTT-----------K----EVRKRVYV
Gh_A12G1286        KGFQRDQNL-QLHRRGHNLPWKLK--------QRST-----------K----EVRKRVYV
Gh_D12G1408        KGFQRDQNL-QLHRRGHNLPWKLK--------QRST-----------K----EVRKRVYV
```

```
Gh_A10G0519          KGFQRDQNL-QLHRRGHNLPWKLK--------QRTS-----------K----EVRKKVYV
Gh_D10G0557          KGFQRDQNL-QLHRRGHNLPWKLK--------QRTS-----------K----EVRKKVYV
Gh_D05G1287          KGFQRDQNL-QLHRRGHNLPWKLK--------QRTN-----------K----EVRKKVYV
Gh_A05G1117          KGFQRDQNL-QLHRRGHNLPWKLK--------QRTN-----------K----EVRKKVYV
Gh_D11G1304          KGFQRDQNL-QLHRRGHNLPWKLK--------QRNS-----------K----EVKKKAYV
Gh_A11G1150          KGFQRDQNL-QLHRRGHNLPWKLK--------QRNS-----------K----EVKKKAYV
Gh_A12G0016          KGFQRDQNL-QLHRRGHNLPWKLK--------QRNN-----------K----EIKKRAYV
Gh_D12G0018          KGFQRDQNL-QLHRRGHNLPWKLK--------QRNN-----------K----EIKKRAYV
Gh_A08G0586          KGFQRDQNL-QLHRRGHNLPWKLK--------QRS------------K----EMKKKVYV
Gh_D08G0679          KGFQRDQNL-QLHRRGHNLPWKLK--------QRS------------K----EMKKKVYV
Gh_D11G2734          KGFQRDQNL-QLHRRGHNLPWKLK--------QRTS-----------T----EIRKRVYV
Gh_A11G2417          KGFQRDQNL-QLHRRGHNLPWKLK--------QRTS-----------T----EIRKRVYV
Gh_D06G0903          KGFQRDQNL-QLHRRGHNLPWKLK--------QRTN-----------K---EVVRKKVYV
Gh_A06G0780          KGFQRDQNL-QLHRRGHNLPWKLK--------QRTN-----------K---EVVRKKVYV
Gh_A05G0322          KGFQRDQNL-QLHRRGHNLPWKLK--------QRTN-----------K---EVVRKKVYV
Gh_D05G0428          KGFQRDQNL-QLHRRGHNLPWKLK--------QRTN-----------K---EVVRKKVYV
Gh_D11G1873          KGFQRDQNL-QLHRRGHNLPWKLR--------QRSS-----------K----EVKKRVYV
Gh_A11G1715          KGFQRDQNL-QLHRRGHNLPWKLK--------QRSS-----------K----EVKKRVYV
Gh_A02G1568          KGFQRDQNL-QLHRRGHNLPWKLR--------QRSS-----------K----EVKKRVYV
Gh_D03G0157          KGFQRDQNL-QLHRRGHNLPWKLR--------QRSS-----------K----EVKKRVYV
Gh_A02G1566          KGFQRDQNL-QLHRRGHNLPWKLR--------QRSS-----------K----EVKKKVYV
Gh_D03G0158          KGFQRDQNL-QLHRRGHNLPWKLR--------QRSS-----------K----EVKKKVYV
Gh_A06G0089          KGFQREQNL-QLHRRGHNLPWKLR--------QRTD-----------GN--ETRKKRVYV
Gh_D06G0062          KGFQRDQNL-QLHRRGHNLPWKLR--------QRTD-----------GN--ETRKKRVYV
Gh_D05G1861          KGFQRDQNL-QLHRRGHNLPWKLR--------QRTS-----------K----EIRKRVYV
Gh_A05G1677          KGFQRDQNL-QLHRRGHNLPWKLR--------QRTS-----------K----EIRKRVYV
                                 *   :

Gh_A07G1366          EGSEEKQHQQMVPSLAATTDNKKEIEQQQDIVNKPEASTTGPAEAKRNLRNPSEARGRGL
Gh_D07G2456          EGSEEKQHQQMVPSLAATTDNKKENEQQQDIVNKPEASTTGPAEAKGNLRNPSEARGRGL
Gh_D07G0695          --------------PAASSSDGGVS-VQNPVAAQPEASK-------EDLE-------TKK
Gh_D12G1787          --------------PAADGCSGADAQKSKKMIARSEAAK-------EDLE-------TKK
Gh_D12G1778          --------------LCNIWCV----------------------------------D-ANA
Gh_A09G1165          --------------CCAPGCR-------------------------NNIDHP---R-AKP
Gh_D09G1170          --------------CCAPGCR-------------------------NNIDHP---R-AKP
Gh_A02G1056          --------------CCAPGCR-------------------------NNIDHP---R-AKP
Gh_D03G0634          --------------CCAPGCR-------------------------NNIDHP---R-AKP
Gh_D01G1184          --------------CCAPGCR-------------------------NNIDHP---R-PKP
Gh_A01G1108          --------------CCAPGCR-------------------------NNIDHP---R-PKP
Gh_D08G0325          --------------CCSPGCK-------------------------HNIDNP---R-AKP
Gh_A08G0236          --------------CCSPGCK-------------------------HNIDNP---R-AKP
Gh_A13G2132          --------------CCAEGCK-------------------------NNIDHP---R-SRP
Gh_D13G0718          --------------CCAEGCK-------------------------NNIDHP---R-SRP
Gh_D06G0302          --------------CCAEGCK-------------------------NNIEHP---R-AKP
Gh_A06G0282          --------------CCAEGCK-------------------------NNIEHP---R-AKP
Gh_A11G1476          --------------CCAPGCK-------------------------HNIDHP---R-ARA
Gh_D11G1633          --------------CCAPGCK-------------------------HNIDHP---R-ARA
Gh_D08G0579          --------------CCAPGCK-------------------------HNIEHP---R-ARP
Gh_A08G0493          --------------CCAPGCK-------------------------HNIEHP---R-ARP
Gh_D13G2257          --------------CCAPGCR-------------------------NNIDHP---R-SKP
Gh_A13G1869          --------------CCAPGCR-------------------------NNIDHP---R-SKP
Gh_A09G0899          --------------CCAHGCK-------------------------NNINHP---R-AKP
Gh_D09G0924          --------------CCAHGCK-------------------------NNINHP---R-AKP
Gh_A07G0996          --------------CCAQGCK-------------------------NNINHP---R-AKP
Gh_D07G1074          --------------CCAQGCK-------------------------NNINHP---R-AKP
Gh_D11G0559          --------------CPYEGCN-------------------------RNKKHK---K-FRP
Gh_A11G0481          --------------CPYEGCN-------------------------RNKKHK---K-FRP
Gh_D11G0560          --------------CPYEGCN-------------------------RNKKHH---K-FRS
Gh_A11G0482          --------------CPYEGCN-------------------------RNKKHH---K-FRS
Gh_A02G0617          --------------CPFAGCK-------------------------RNKDHK---K-FQP
Gh_D02G0671          --------------CPFAGCK-------------------------RNKDHK---K-FQP
Gh_A09G1098          --------------CPYAGCK-------------------------RNKDHK---K-FQP
Gh_D09G1104          --------------CPYAGCK-------------------------RNKDHK---K-FQP
```

```
Gh_A09G0835         --------------CPYEGCR-------------------------WNQKHA---K-FQP
Gh_D09G0856         --------------CPYDGCR-------------------------WNQKHA---N-FQP
Gh_D05G0854         --------------CPQEGCR-------------------------WNQKHA---K-FQP
Gh_A05G0718         --------------CPQEGCR-------------------------WNQKHA---K-FQP
Gh_D12G1536         ------------------------------------------------HKDP--------
Gh_A05G2664         --------------CPEPSCL---------------------------HHDP-----CHA
Gh_D05G2965         --------------CPEPSCL---------------------------HHDP-----CHA
Gh_D02G1382         --------------CPEPSCL---------------------------HHHP-----SHA
Gh_A03G0994         --------------CPEPSCL---------------------------HHQP-----SHA
Gh_D01G1332         --------------CPEPTCL---------------------------HHDP-----CHA
Gh_Sca004794G01     --------------CPEPTCL---------------------------HHDP-----CHA
Gh_A12G2459         --------------CPEPSCL---------------------------HHQP-----CHA
Gh_D12G2587         --------------CPDPSCL---------------------------HHQP-----CHA
Gh_A11G0018         --------------CPEPTCL---------------------------HHDP-----CHA
Gh_D11G0016         --------------CPEPTCL---------------------------HHDP-----CHA
Gh_A03G0782         --------------CPEPTCL---------------------------HHDP-----CHA
Gh_D02G1197         --------------CPEPTCL---------------------------HHDP-----CHA
Gh_D09G1639         --------------CPEPSCL---------------------------HHDP-----CHA
Gh_A09G1568         --------------CPEPSCL---------------------------HHDP-----CHA
Gh_D02G1400         --------------CPEPTCV---------------------------HHDP-----SKA
Gh_A12G1411         --------------CPEPTCV---------------------------HHEP-----SRA
Gh_D12G1529         --------------CPEPTCV---------------------------HHEP-----SRA
Gh_A12G1412         --------------CPEPTCV---------------------------HHDP-----SRA
Gh_D12G1530         --------------CPEPTCV---------------------------HHDP-----SRA
Gh_D01G1353         --------------CPEPTCV---------------------------HHDP-----SRA
Gh_A01G2114         --------------CPEPTCV---------------------------HHDP-----SRA
Gh_A09G0997         --------------CPEKTCV---------------------------HHDP-----SRA
Gh_D09G1017         --------------CPEKTCV---------------------------HHDP-----SRA
Gh_D09G1416         --------------CPEKTCV---------------------------HHDP-----SRA
Gh_A09G1411         --------------CPEKTCV---------------------------HHDP-----SRA
Gh_A04G0633         --------------CPEKTCV---------------------------HHDP-----SRA
Gh_D04G1096         --------------CPEKTCV---------------------------HHDP-----SRA
Gh_D02G1798         --------------CPEPNCV---------------------------HHHP-----SRA
Gh_A03G1358         --------------CPEPNCV---------------------------HHHP-----SRA
Gh_A12G0073         --------------CPEPSCV---------------------------HHNA-----ARA
Gh_D12G0089         --------------CPEPSCV---------------------------HHNA-----ARA
Gh_D08G0786         --------------CPEPTCV---------------------------HHNP-----ARA
Gh_A08G0678         --------------CPEPTCV---------------------------HHNP-----ARA
Gh_D11G1217         --------------CPEPTCV---------------------------HHNP-----ARA
Gh_A11G1062         --------------CPEPTCV---------------------------HHNP-----ARA
Gh_D02G1806         --------------CPEKTCV---------------------------HHHP-----SRA
Gh_A03G1366         --------------CPEKTCV---------------------------HHHP-----SRA
Gh_A12G1286         --------------CPEKTCV---------------------------HHHP-----SRA
Gh_D12G1408         --------------CPEKTCV---------------------------HHHP-----SRA
Gh_A10G0519         --------------CPEVSCV---------------------------HHDP-----SRA
Gh_D10G0557         --------------CPEVSCV---------------------------HHDP-----SRA
Gh_D05G1287         --------------CPETNCV---------------------------HHDP-----SRA
Gh_A05G1117         --------------CPETNCV---------------------------HHDP-----SRA
Gh_D11G1304         --------------CPEPTCV---------------------------HHHP-----SRA
Gh_A11G1150         --------------CPEPTCV---------------------------HHHP-----SRA
Gh_A12G0016         --------------CPEPTCV---------------------------HHHP-----SRA
Gh_D12G0018         --------------CPEPTCV---------------------------HHHP-----SRA
Gh_A08G0586         --------------CPEPRCV---------------------------HHHP-----SRA
Gh_D08G0679         --------------CPEPRCV---------------------------HHHP-----SRA
Gh_D11G2734         --------------CPEPSCV---------------------------HHNP-----ARA
Gh_A11G2417         --------------CPEPSCV---------------------------HHNP-----ARA
Gh_D06G0903         --------------CPETTCV---------------------------HHDP-----ARA
Gh_A06G0780         --------------CPETTCV---------------------------HHDP-----ARA
Gh_A05G0322         --------------CPETTCV---------------------------HHHP-----SRA
Gh_D05G0428         --------------CPETTCV---------------------------HHHP-----SRA
Gh_D11G1873         --------------CPEITCV---------------------------HHHP-----SRA
Gh_A11G1715         --------------CPEITCV---------------------------HHHP-----SRA
Gh_A02G1568         --------------CPEPTCV---------------------------HHDP-----SRA
```

```
Gh_D03G0157          --------------CPEPTCV---------------------------HHDP-----SRA
Gh_A02G1566          --------------CPEPTCV---------------------------HHDP-----SRA
Gh_D03G0158          --------------CPEPTCV---------------------------HHDP-----SRA
Gh_A06G0089          --------------CPETSCV---------------------------HHNP-----ARA
Gh_D06G0062          --------------CPETSCV---------------------------HHNA-----ARA
Gh_D05G1861          --------------CPEPSCV---------------------------HHNP-----ARA
Gh_A05G1677          --------------CPEPSCV---------------------------HHNP-----ARA


Gh_A07G1366          KRKMRGGRGGKYVKRNEGSRRPSEPPKPKG-GIPFMCELCNVKCETQVVFNCHLAGKKHI
Gh_D07G2456          KRKMRGGRGGKYVKRNEGSRRPSEPPKPKG-GIPFMCELCNVKCETQVVFNCHLAGKKHI
Gh_D07G0695          LKVMEGGTA--------------------A-ADVRVCTICNVVCNSEKVFKYHLTGQKHA
Gh_D12G1787          QKVMEGGAA--------------------A-TAIRVCVICNVVCNSQTVFNYHLTGQKHA
Gh_D12G1778          LKQHLAGKK--------------H--------------------KKM--QGKVATVEGDI
Gh_A09G1165          LKDFRTLQT--------------HYKRKHG-IKPFMCGKCG-KAFAV--RGDWRTHEKNC
Gh_D09G1170          LKDFRTLQT--------------HYKRKHG-IKPFMCRKCG-KTFAV--RGDWRTHEKNC
Gh_A02G1056          LKDFRTLQT--------------HYKRKHG-IKPFMCRKCG-KAFAV--RGDWRTHEKNC
Gh_D03G0634          LKDFRTLQT--------------HYKRKHG-IKPFMCRKCG-KAFAV--RGDWRTHEKNC
Gh_D01G1184          LKDFRTLQT--------------HYKRKHG-IKPFMCRKCG-KAFAV--RGDWRTHEKNC
Gh_A01G1108          LKDFRTLQT--------------HYKRKHG-IKPFMCRKCG-KAFAV--RGDWRTHEKNC
Gh_D08G0325          LKDFKTLQT--------------HYKRKHG-IKPFVCRKCE-KAFAV--KGDWRTHEKNC
Gh_A08G0236          LKDFKTLQT--------------HYKRKHG-IKPFVCRKCE-KAFAV--KGDWRTHEKNC
Gh_A13G2132          LKDFRTLQT--------------HYKRKHG-AKPFECRKCG-KPFAV--RGDWRTHEKNC
Gh_D13G0718          LKDFRTLQT--------------HYKRKHG-AKPFECRKCG-KPFAV--RGDWRTHEKNC
Gh_D06G0302          LKDFRTLQT--------------HYKRKHG-LKPFMCRKCG-KFLAV--KGDWRTHEKNC
Gh_A06G0282          LKDFRTLQT--------------HYKRKHG-LKPFMCRKCG-KFLAV--KGDWRTHEKNC
Gh_A11G1476          LKDFRTLQT--------------HYKRKHG-IKPFTCRKCE-KAFAV--KGDWRTHEKNC
Gh_D11G1633          LKDFRTLQT--------------HYKRKHG-IKPFNCRKCE-KAFAV--KGDWRTHEKNC
Gh_D08G0579          LKDFRTLQT--------------HYKRKHG-VKPFTCRKCD-KAFAV--KGDWRTHEKNC
Gh_A08G0493          LKDFRTLQT--------------HYKRKHG-VKPFTCRKCD-KAFAV--KGDWRTHEKNC
Gh_D13G2257          LKDFRTLQT--------------HYKRKHG-IKAFMCRKCG-KSFAV--RGDWRTHEKNC
Gh_A13G1869          LKDFRTLQT--------------HYKRKHG-IKAFMCRKCG-KSFAV--RGDWRTHEKNC
Gh_A09G0899          LKDFRTLQT--------------HYKRKHG-AKPFMCRKCG-KPFAV--KGDWRTHEKNC
Gh_D09G0924          LKDFRTLQT--------------HYKRKHG-AKPFMCRKCG-KPFAV--KGDWRTHEKNC
Gh_A07G0996          LKDFRTLQT--------------HYKRKHG-AKPFMCRKCG-KTFAV--KGDWRTHEKNC
Gh_D07G1074          LKDFRTLQT--------------HYKRKHG-AKPFMCRKCG-KTFAV--KGDWRTHEKNC
Gh_D11G0559          LKSVICVRN--------------HFKRSHC-PKMYCCNRCHKKSFSV--LADLKAHLKNC
Gh_A11G0481          LKSVICVRN--------------HFKRSHC-PKMYCCNRCHKKSFSV--LADLKAHLKNC
Gh_D11G0560          LKSVICVRN--------------HFKRSHC-PKMYRCNRCHKKSFSI--LVDLKVHLKHC
Gh_A11G0482          LKSVICVRN--------------HFKRSHC-PKMYRCNRCHKKSFSI--LVDLKAHLKNC
Gh_A02G0617          LKTILCVKN--------------HYKRSHC-DKSYICSRCHTKKFSV--IADLKTHEKHC
Gh_D02G0671          LKTILCVKN--------------HYKRSHC-DKSYICSRCHTKKFSV--IADLKTHEKHC
Gh_A09G1098          LKTILCVKN--------------HYKRTHC-DKSYICSRCNTKKFSV--IADLKTHEKHC
Gh_D09G1104          LKTILCVKN--------------HYKRTHC-DKSYICSRCNTKKFSV--IADLKTHEKHC
Gh_A09G0835          LKSMICVKN--------------HYKRSHC-PKMYVCKRCNRKQFSV--LSDLRTHEKHC
Gh_D09G0856          LKSMICVKN--------------HYKRSHC-PKMYVCKRCNRKQFSL--LSDLRTHEKHC
Gh_D05G0854          LKSMICVKN--------------HYKRSHC-PKMYVCKRCNCKQFSV--LSDLRTHEKHC
Gh_A05G0718          LKSMICVKN--------------HYKRSHC-PKMYVCKRCNCKQFSV--LSDLRTHEKHC
Gh_D12G1536          ------------------------------------------------------------
Gh_A05G2664          LGDLVGIKK--------------HFRRKHSKQKQWVCDKCC-KGYAV--QSDFKAHLKIC
Gh_D05G2965          LGDLVGIKK--------------HFRRKHSKQKQWVCDKCR-KGYAV--QSDFKAHLKTC
Gh_D02G1382          LGDLVGIKK--------------HFRRKHSNQRKWVCDKCS-KGYAV--QSDYKAHLKIC
Gh_A03G0994          LGDLVGIKK--------------HFRRKHSNQRKWVCEKCS-KGYAV--QSDYKAHLKIC
Gh_D01G1332          LGDLVGIKK--------------HFRRKHSNQKQWVCEKCS-KGYAV--QSDYKAHLKTC
Gh_Sca004794G01      LGDLVGIKK--------------HFRRKHSNQKQWVCEKCS-KGYAV--QSDYKAHLKTC
Gh_A12G2459          LGDLVGIKK--------------HFRRKHSNHKQWVCEKCS-KGYAV--QSDYKAHLKTC
Gh_D12G2587          LGDLVGIKK--------------HFRRKHSNHKQWVCEKCS-KGYAV--QSDYKAHLKTC
Gh_A11G0018          LGDLVGIKK--------------HFRRKHSNHKQWVCQKCS-KGYAV--QSDYKAHLKTC
Gh_D11G0016          LGDLVGIKK--------------HFRRKHSNHKQWVCQKCS-KGYAV--QSDYKAHLKTC
Gh_A03G0782          LGDLVGIKK--------------HFRRKHSNHKQWVCDKCS-KAYAV--QSDYKAHLKTC
Gh_D02G1197          LGDLVGIKK--------------HFRRKHSNHKQWVCDKCS-KAYAV--QSDYKAHLKTC
Gh_D09G1639          LGDLVGIKK--------------HFRRKHSNHKQWVCQKCS-KGYAV--QSDYKAHLKTC
Gh_A09G1568          LGDLVGIKK--------------HFRRKHSNHKQWVCQKCS-KGYAV--QSDYKAHLKTC
```

```
Gh_D02G1400          LGDLTGIKK--------------HYSRKHG-EKKWKCEKCS-KRYAV--QSDWKAHSKTC
Gh_A12G1411          LGDLTGIKK--------------HYSRKHG-EKKWKCEKCS-KRYAV--QSDWKAHSKTC
Gh_D12G1529          LGDLTGIKK--------------HYSRKHG-EKKWKCEKCS-KRYAV--QSDWKAHSKTC
Gh_A12G1412          LGDLTGIKK--------------HYSRKHG-EKKWKCEKCS-KRYAV--QSDWKAHSKTC
Gh_D12G1530          LGDLTGIKK--------------HYSRKHG-EKKWKCEKCS-KRYAV--QSDWKAHSKTC
Gh_D01G1353          LGDLTGIKK--------------HYSRKHG-EKKWKCEKCS-KRYAV--QSDWKAHSKTC
Gh_A01G2114          LGDLTGIKK--------------HYSRKHG-EKKWKCEKCS-KRYAV--QSDWKAHSKTC
Gh_A09G0997          LGDLTGIKK--------------HFSRKHG-EKKWKCEKCS-KKYAV--QSDWKAHSKIC
Gh_D09G1017          LGDLTGIKK--------------HFSRKHG-EKKWKCEKCS-KKYAV--QSDWKAHSKIC
Gh_D09G1416          LGDLTGIKK--------------HFSRKHG-EKKWKCEKCS-KKYAV--QSDWKAHSKTC
Gh_A09G1411          LGDLTGIKK--------------HFSRKHG-EKKWKCEKCS-KKYAV--QSDWKAHSKTC
Gh_A04G0633          LGDLTGIKK--------------HFSRKHG-EKKWKCEKCS-KKYAV--QSDWKAHSKTC
Gh_D04G1096          LGDLTGIKK--------------HFSRKHG-EKKWKCEKCS-KKYAV--QSDWKAHSKTC
Gh_D02G1798          LGDLTGIKK--------------HFCRKHG-EKKWKCDKCS-KRYAV--QSDWKAHAKIC
Gh_A03G1358          LGDLTGIKK--------------HFCRKHG-EKKWKCDKCS-KRYAV--QSDWKAHAKIC
Gh_A12G0073          LGDLTGIKK--------------HFSRKHG-EKKWKCDKCS-KKYAV--QSDWKAHQKTC
Gh_D12G0089          LGDLTGIKK--------------HFSRKHG-EKKWKCDKCS-KKYAV--QSDWKAHQKTC
Gh_D08G0786          LGDLTGIKK--------------HFSRKHG-EKKWKCDKCS-KKYAV--QSDWKAHQKTC
Gh_A08G0678          LGDLTGIKK--------------HFSRKHG-EKKWKCDKCS-KKYAV--QSDWKAHQKTC
Gh_D11G1217          LGDLTGIKK--------------HFSRKHG-EKKWKCDKCS-KKYAV--QSDWKAHQKTC
Gh_A11G1062          LGDLTGIKK--------------HFSRKHG-EKKWKCDKCS-KKYAV--QSDWKAHQKTC
Gh_D02G1806          LGDLTGIKK--------------HFYRKHG-EKKWKCEKCS-KRYAV--QSDWKAHSKTC
Gh_A03G1366          LGDLTGIKK--------------HFYRKHG-EKKWKCEKCS-KRYAV--QSDWKAHSKTC
Gh_A12G1286          LGDLTGIKK--------------HFCRKHG-EKKWKCDKCS-KRYAV--QSDWKAHSKTC
Gh_D12G1408          LGDLTGIKK--------------HFCRKHG-EKKWKCDKCS-KQYAV--QSDWKAHSKTC
Gh_A10G0519          LGDLTGIKK--------------HFYRKHG-EKKWKCDKCS-KRYAV--QSDWKAHSKTC
Gh_D10G0557          LGDLTGIKK--------------HFYRKHG-EKKWKCDKCS-KRYAV--QSDWKAHSKTC
Gh_D05G1287          LGDLTGIKK--------------HFSRKHG-EKKWKCEKCS-KKYAV--QSDWKAHSKTC
Gh_A05G1117          LGDLTGIKK--------------HFSRKHG-EKKWKCEKCS-KKYAV--QSDWKAHSKTC
Gh_D11G1304          LGDLTGIKK--------------HYCRKHG-EKKWKCEKCS-KIYAV--QSDWKAHSKTC
Gh_A11G1150          LGDLTGIKK--------------HYCRKHG-EKKWKCEKCS-KIYAV--QSDWKAHSKTC
Gh_A12G0016          LGDLTGIKK--------------HYCRKHG-EKKWKCEKCS-KIYAV--QSDWKAHTKTC
Gh_D12G0018          LGDLTGIKK--------------HYCRKHG-EKKWKCEKCS-KIYAV--QSDWKAHTKTC
Gh_A08G0586          LGDLTGIKK--------------HFCRKHG-EKKWKCEKCS-KIYAV--QSDWKAHSKIC
Gh_D08G0679          LGDLTGIKK--------------HFCRKHG-EKKWKCEKCS-KIYAV--QSDWKAHSKIC
Gh_D11G2734          LGDLTGIKK--------------HFCRKHG-EKKWKCDKCS-KKYAV--QSDWKAHSKTC
Gh_A11G2417          LGDLTGIKK--------------HFCRKHG-EKKWKCDKCS-KKYAV--QSDWKAHSKTC
Gh_D06G0903          LGDLTGIKK--------------HFCRKHG-EKKWKCEKCS-KTYAV--QSDWKAHSKIC
Gh_A06G0780          LGDLTGIKK--------------HFCRKHG-EKKWKCEKCS-KTYAV--QSDWKAHSKIC
Gh_A05G0322          LGDLTGIKK--------------HFCRKHG-EKKWKCEKCS-KRYAV--QSDWKSHSKIC
Gh_D05G0428          LGDLTGIKK--------------HFCRKHG-EKKWKCEKCS-KRYAV--QSDWKSHSKIC
Gh_D11G1873          LGDLTGIKK--------------HFCRKHG-EKKWKCDKCS-KKYAV--QSDWKAHSKIC
Gh_A11G1715          LGDLTGIKK--------------HFCRKHG-EKKWKCDKCS-KKYAV--QSDWKAHSKIC
Gh_A02G1568          LGDLTGIKK--------------HFCRKHG-EKKWKCDKCS-KKYAV--QSDWKAHSKIC
Gh_D03G0157          LGDLTGIKK--------------HFCRKHG-EKKWKCDKCS-KKYAV--QSDWKAHSKIC
Gh_A02G1566          LGDLTGIKK--------------HFCRKHG-EKKWKCDKCS-KKYAV--QSDWKAHSKIC
Gh_D03G0158          LGDLTGIKK--------------HFCRKHG-EKKWKCDKCS-KKYAV--QSDWKAHSKIC
Gh_A06G0089          LGDLTGIKK--------------HYCRKHC-EKKWRCERCS-KTYAV--KSDWTAHIKSC
Gh_D06G0062          LGDLTGIKK--------------HYCRKHC-EKKWRCERCS-KTYAV--KSDWTAHLKSC
Gh_D05G1861          LGDLTGIKK--------------HFCRKHG-ERKWKCERCS-KKYAV--QSDWKAHMKTC
Gh_A05G1677          LGDLTGIKK--------------HFCRKHG-EKKWKCERCS-KKYAV--QSDWKAHMKTC


Gh_A07G1366          AN-------------------------MKRFHGHRALYGEAGVQALYPPNISAPPPP-LV
Gh_D07G2456          AN-------------------------MKRFHGHRALYGEAGVQALYPPNISAPPPP-LV
Gh_D07G0695          AM-------------------------VKKQAATTS------------------------
Gh_D12G1787          TM-------------------------VKKLANGRIPTVT--------------------
Gh_D12G1778          GE----ELKCELCGIGCP--------SKDLLHLHFN---GKKHQEQLRK-----------
Gh_A09G1165          GK-----LWYCTCGSDFK--------HKRSLKDHIKAFGKG-HAAYNI-TIS-----GLE
Gh_D09G1170          GK-----LWYCTCGSDFK--------HKRSLKDHIKAFGNG-HAAYDI-TIS-----GLE
Gh_A02G1056          GK-----LWYCICGSDFK--------HKRSLKDHIKAFGNG-HAAYGI-D-------GME
Gh_D03G0634          GK-----LWYCICGSDFK--------HKRSLKDHIKAFGNG-HAAYGI-D-------GLE
Gh_D01G1184          GK-----LWYCICGSDFK--------HKRSLKDHIKAFGDG-HQVYAT-D-------DLE
```

```
Gh_A01G1108          GK-----LWYCICGSDFK--------HKRSLKDHIKAFGDG-HQVYAA-D-------DLE
Gh_D08G0325          GK-----VWYCICGSDFK--------HKRSLKDHTKAFGPTGHGA-----VG-----GVD
Gh_A08G0236          GK-----VWYCICGSDFK--------HKRSLKDHTKAFGPTGHGA-----VG-----GVD
Gh_A13G2132          GK-----LWFCICGSDFK--------HKRSLKDHVRAFGDG-HAPHTV-EYC-----EVE
Gh_D13G0718          GK-----LWFCICGSDFK--------HKRSLKDHVRAFGDG-HAPHTV-EYC-----EVE
Gh_D06G0302          GK-----RWLCVCGSDFK--------HKRSLKDHIKAFGSG-HGPFPP-SFD-----GVE
Gh_A06G0282          GK-----RWLCVCGSDFK--------HKRSLKDHIKAFGSG-HGPFPP-SFD-----GVE
Gh_A11G1476          GK-----IWYCICGSDFK--------HKRSLKDHIKAFGPG-HGSVG-----------IA
Gh_D11G1633          GK-----IWYCICGSDFK--------HKRSLKDHIKAFGAG-HGSVG-----------IA
Gh_D08G0579          GK-----IWYCICGSDFK--------HKRSLKDHIKAFGPG-HAAVG-----------ID
Gh_A08G0493          GK-----IWYCICGSDFK--------HKRSLKDHIKAFGPG-HAAVG-----------ID
Gh_D13G2257          GK-----LWYCSCGSDFK--------HKRSLKDHIRAFGNG-HSAYG-----------ID
Gh_A13G1869          GK-----LWYCSCGSDFK--------HKRSLKDHIRAFGNG-HSAYG-----------ID
Gh_A09G0899          GK-----LWYCTCGSDFK--------HKRSLKDHIRSFGNG-HSPHPS--LE-----GFE
Gh_D09G0924          GK-----LWYCTCGSDFK--------HKRSLKDHIRSFGNG-HSPHPS--LE-----GFE
Gh_A07G0996          GK-----LWYCTCGSDFK--------HKRSLKDHIRSFGKG-HSPHPS--LE-----RFE
Gh_D07G1074          GK-----LWYCTCGSDFK--------HKRSLKDHIRSFGKG-HSPHPS--LE-----GFE
Gh_D11G0559          GVGEESKKWKCSCGNGFS--------RKDKLFGHISLFEG--HMPAVAAEEDKKLKGVVA
Gh_A11G0481          GVGEERKKWKCSCGNGFS--------RKDKLFGHISLFEG--HMPAVAAEEDQKLKGVVA
Gh_D11G0560          GGKEEIKKWKCSCGNGFS--------RKDKLFRHISLFEG--HMPAMVAEEDRKLKGIVA
Gh_A11G0482          GGGEEIKKWKCSCGNGFS--------RKDKLFRHISLFEG--HMPAMVAEEDQKLKGIVV
Gh_A02G0617          GK----DKWLCSCGTTFS--------RKDKLFGHIALFQG--HTPAIPLDETKGPTAPSD
Gh_D02G0671          GK----DKWLCSCGTTFS--------RKDKLFGHIALFQG--HTPAIPLDETKGPIAPSD
Gh_A09G1098          GK----DKWLCSCGTTFS--------RKDKLFGHITLFQG--HTPAIPSDEIKVAPAGT-
Gh_D09G1104          GK----DKWLCSCGTTFS--------RKDKLFGHITLFQG--HTPAIPSDEIKAAPAGT-
Gh_A09G0835          GD----LRWQCSCGNTFS--------RKDKLMGHVALFVG--HRPVVAHSLH--------
Gh_D09G0856          GD----LRWQCSCGNTFS--------RKDKLMGHVALFVG--HRPVVAHSLH--------
Gh_D05G0854          GD----LKWQCSCGTTFS--------RKDKLMGHVALFVG--HTPALVTNPKANPG----
Gh_A05G0718          GD----LKWQCSCGTTFS--------RKDKLMGHVALFVG--HTPALATNPMANPG----
Gh_D12G1536          ------------------------RPQVESFIEHQDICSVRGVQHQFQALQ-------YS
Gh_A05G2664          GT----RGHSCDCGRVFS--------RVESFIEHQDSCSARRIQPELLTAV--LPSC-SS
Gh_D05G2965          GT----RGHSCDCGRVFS--------RVESFIEHQDSCSARPIQPELLTAV--MPSC-SS
Gh_D02G1382          GT----RGHSCDCGRVFS--------RVESFIEHQDACSVRGVQPELQAV---HPGC-SS
Gh_A03G0994          GT----RGHSCDCGRVFS--------RVESFIEHQDACSVRGVQPELQAV---HPGC-SS
Gh_D01G1332          GT----RGHSCDCGRVFS--------RVESFIEHQDACTIRRVQPELQAQLQPAAAC-SS
Gh_Sca004794G01      GT----RGHSCDCGRVFS--------RVESFIEHQDACTTRRVQPELQAQLQPAAAC-SS
Gh_A12G2459          GT----RGHSCDCGRVFS--------RVESFIEHQDACRMGQSQKVQ------QPPS-LS
Gh_D12G2587          GT----RGHSCDCGRVFS--------RVESFIEHQDACRMGQSQKVQ------QPPS-LS
Gh_A11G0018          GT----RGHSCDCGRVFSRHFLLVFDRVESFIEHQDACHMGRV-GQEPQAL--QPAC-LS
Gh_D11G0016          GT----RGHSCDCGRVFSRHFLLVFDRVESFIEHQDACHMGRV-SQEPQAL--QPAC-LS
Gh_A03G0782          GT----RGHSCDCGRVFS--------RVESFIEHQDACHMGRVIRPESQGV--QLAC-LL
Gh_D02G1197          GT----RGHSCDCGRVFS--------RVESFIEHQDACHMGRVIRPESQGV--QLAC-LS
Gh_D09G1639          GT----RGHSCDCGRVFS--------RVESFIEHQDACHMSRS-RPQSQAM--QPVC-LS
Gh_A09G1568          GT----RGHSCDCGRVFS--------RVESFIEHQDACHMSRS-RPQSQAM--QPIC-LS
Gh_D02G1400          GT----REYRCDCGTLFS--------RRDSFITHRAFCDALAQESARHPTN-LSTMG-SH
Gh_A12G1411          GT----REYRCDCGTLFS--------RRDSFITHRAFCDALAQETARHPTNNLTPIG-SH
Gh_D12G1529          GT----REYRCDCGTLFS--------RRDSFITHRAFCDALAQETARHPTNNLTPIG-SH
Gh_A12G1412          GT----REYRCDCGTLFS--------RRDSFITHRAFCDALAQETARHPPP-LNSIG-T-
Gh_D12G1530          GT----REYRCDCGTLFS--------RRDSFITHRAFCDALAQETARHPPP-LNSIG-T-
Gh_D01G1353          GT----REYRCDCGTLFS--------RRDSFITHRAFCDALAQENARQPPS-FNSIG-N-
Gh_A01G2114          GT----REYRCDCGTLFS--------RRDSFITHRAFCDALAQENARQPPS-FNSIG-N-
Gh_A09G0997          GT----REYRCDCGTLFS--------RKDSFITHRAFCDALAEESGRFTSVSTTTMN-PS
Gh_D09G1017          GT----REYRCDCGTLFS--------RKDSFITHRAFCDALAEESGRFTSVSTTTMN-PS
Gh_D09G1416          GT----REYKCDCGTLFS--------RKDSFITHRAFCDALAEESARLTSVA--ATS-L-
Gh_A09G1411          GT----REYKCDCGTLFS--------RKDSFITHRAFCDALAEESARLTSVA--ATS-L-
Gh_A04G0633          GT----REYKCDCGTLFS--------RKDSFITHRAFCDALAEESARFSSVAGAATN-L-
Gh_D04G1096          GT----REYKCDCGTLFS--------RKDSFITHRAFCDALAEESARFSSVAGAATN-L-
Gh_D02G1798          GT----REYRCDCGTIFS--------RKDSFVTHRAFCDALTEENYKVNRNHGAIGG-SI
Gh_A03G1358          GT----REYRCDCGTIFS--------RKDSFVTHRAFCDALTEENYKVNRNHGAIGG-SI
Gh_A12G0073          GT----KEYKCDCGTIFA--------RRDSFITHRAFCDALAEENNKVNNNHGALMN-NM
Gh_D12G0089          GT----KEYKCDCGTIFA--------RRDSFITHRAFCDALAEENNKVNNNHGALMN-NM
Gh_D08G0786          GT----REYKCDCGTIFS--------RRDSFITHRAFCDALAEENNKVNQ---GIMN-NM
Gh_A08G0678          GT----REYKCDCGTIFS--------RRDSFITHRAFCDALAEENNKVNQ---GIMN-NM
```

```
Gh_D11G1217           GT----KEYKCDCGTIFS--------RRDSFITHRAFCDAIAEENIKVNQ---GLMN-NA
Gh_A11G1062           GT----KEYKCDCGTIFS--------RRDSFITHRAFCDAIAEENIKVNQ---GLMN-NA
Gh_D02G1806           GT----REYKCDCGTLFS--------RRDSFITHRAFCDALAEETARVNAASSMHSL-AT
Gh_A03G1366           GT----REYKCDCGTLFS--------RRDSFITHRAFCDALAEETARVNAASSMHSL-AT
Gh_A12G1286           GT----REYKCDCGTLFS-------SRRDSFITHRAFCDALAEETARVNAASSMHSN---
Gh_D12G1408           GT----REYKCDCGTLFS-------SRRDNFITHRAFCDALAEETARVNAASSMHSN---
Gh_A10G0519           GS----KEYRCDCGTLFS--------RRDSFITHRAFCDALAEESSRAAIMGANTF----
Gh_D10G0557           GT----KEYRCDCGTLFS--------RRDSFITHRAFCDALAEESSRAAIMGANTF----
Gh_D05G1287           GT----REYRCDCGTLFS--------RRDSFITHRAFCDALAVESARAINPLLSPH----
Gh_A05G1117           GT----REYRCDCGTLFS--------RRDSFITHRAFCDALAVESARAINPLLSPH----
Gh_D11G1304           GT----REYRCDCGTLFS--------RKDSFITHRAFCDALAEESARLSANQLAAAA---
Gh_A11G1150           GT----REYRCDCGTLFS--------RKDSFITHRAFCDALAEESARLSANQLAAAA---
Gh_A12G0016           GT----REYRCDCGTLFS--------RKDSFITHRAFCDALAEESARLSANQLAVAA-AT
Gh_D12G0018           GT----REYRCDCGTLFS--------RKDSFITHRAFCDALAEESARLSANQLAVAA-AT
Gh_A08G0586           GT----REYRCDCGTLFS--------RKDSFITHRAFCDALAEESARFSANNLPSAA-AA
Gh_D08G0679           GT----REYRCDCGTLFS--------RKDSFITHRAFCDALAEESARFSANHLPSGA-AA
Gh_D11G2734           GT----KEYKCDCGTIFS--------RRDSFITHRAFCDALTEENNKASRGLINVGS-NL
Gh_A11G2417           GT----KEYKCDCGTIFS--------RRDSFITHRAFCDALTEENNKASRGLINVGS-NL
Gh_D06G0903           GT----REYRCDCGTLFS--------RRDSFITHRAFCDALAEESARAITGPAQQ----V
Gh_A06G0780           GT----REYRCDCGTLFS--------RRDSFITHRAFCDALAEESARAITGPAQQ----V
Gh_A05G0322           GT----REYRCDCGNLFS--------RRDSFITHRAFCDALAEETARAITAANQP-----
Gh_D05G0428           GT----REYRCDCGNLFS--------RRDSFITHRAFCDALAEETARAITAANQP-----
Gh_D11G1873           GT----REYKCDCGTVFS--------RRDSFITHRAFCDVLAEERAKAQTQPQPQ----N
Gh_A11G1715           GT----REYKCDCGTVFS--------RRDSFITHRAFCDVLAEERAKAQTQPQPQ----N
Gh_A02G1568           GT----REYKCDCGTVFS--------RRDSFITHRAFCDALAVESAKTQTNPSSE----S
Gh_D03G0157           GT----REYKCDCGTVFS--------RRDSFITHRAFCDALAVESAKTQTNPSSE----S
Gh_A02G1566           GT----REYKCDCGTVFS--------RRDSFITRRAFCDALAVESAKTQTNPSSE----P
Gh_D03G0158           GT----REYKCDCGTVFS--------RRDSFVTHRAFCDALAVESAKTQTNPSSE----P
Gh_A06G0089           GT----KEYKCNCGTVFS--------RRDSFYTHRTFCDALAEENARAQA----------
Gh_D06G0062           GT----KEYKCNCGTVFS--------RRDSFYTHRAFCDALAEENARAQA----------
Gh_D05G1861           GT----REYKCDCGTIFS--------RRDSFITHRAFCDALAEESARTQTLGVAN----P
Gh_A05G1677           GT----REYKCDCGTIFS--------RRDSFITHRAFCDALAEESVRTQTIGVAN----P
```
